# Exogenous creatine supplementation promotes tumor metastasis via megakaryocyte creatine kinase B-STAT5B signaling

**DOI:** 10.1038/s41467-026-74639-z

**Published:** 2026-07-15

**Authors:** Sisi Xie, Ruibo Chen, Xiaoting Sun, Xiaolei Ni, Yuting Wu, Linli Cai, Yangyi Chen, Shuangming Lin, Mei Rao, Liling Chen, Yintao Li, Yanyong Xu, Minfeng Chen, Dongmei Zhang, Shun Zhu, Ling Yang, Wen Liu, Ji Zuo, Yunlong Yang

**Affiliations:** 1 Department of Cardiology, Basic Scientific Research Center, Longyan First Hospital Affiliated to Fujian Medical University, Longyan, China; 2 Department of Cellular and Genetic Medicine, School of Basic Medical Sciences, Fudan University, Shanghai, China; 3 School of Pharmaceutical Science, Wenzhou Medical University, Wenzhou, China; 4 Department of Gastroenterology and Anorectal Surgery, Longyan First Hospital, Fujian Medical University, Longyan, China; 5 Department of Respiratory Oncology, Shandong Cancer Hospital and Institute, Shandong First Medical University and Shandong Academy of Medical Sciences, Jinan, China; 6 Key Laboratory of Metabolism and Molecular Medicine of the Ministry of Education, Department of Biochemistry and Molecular Biology of School of Basic Medical Sciences, Fudan University, Shanghai, China; 7 State Key Laboratory of Bioactive Molecules and Druggability Assessment, Jinan University, Guangzhou, China; 8 Guangdong Basic Research Center of Excellence for Natural Bioactive Molecules and Discovery of Innovative Drugs, College of Pharmacy, Jinan University, Guangzhou, China

**Keywords:** Metastasis, Phosphorylation, Platelets

## Abstract

Creatine supplementation is widely used in sports and increasingly popular among exercising individuals. Although the physiological role of creatine has been extensively studied, the creatine biology in pathological conditions remains poorly understood. Here we report that exogenous creatine supplementation promotes tumor metastasis via platelet activation mechanism in various mouse models and humans. Mechanistically, creatine supplementation increases megakaryocyte creatine levels and upregulates creatine kinase B (CKB). Unbiased phosphoproteomics reveals that a CKB-downstream, non-canonical STAT5B phosphorylation instigates various platelet functional genes, leading to hyperactive, metastasis-promoting platelets. Megakaryocyte-specific knockout of the creatine transporter *Slc6a8* or *Stat5b*, as well as pharmacological inhibition of STAT5, ablates the creatine-augmented platelet hyperactivity and prevents consequent metastasis in mice. Importantly, creatine supplementation in healthy volunteers results in hyperactive peripheral platelets that increase metastasis risks. Together, our study sheds mechanistic insights into the creatine-induced metastasis and provides an anti-metastatic therapeutic paradigm by targeting megakaryocyte creatine metabolism.

## Abstract (web summary)

How diet regulates cancer progression and therapeutic response is not completely understood. Here, the authors show that creatine supplementation promotes tumor metastasis by inducing hyperactive platelets in cancer models.

## Introduction

Creatine is abundant in human body that can be both endogenously de novo synthesized from arginine and glycine^1^, and exogenously obtained from meat and, more recently, from dietary supplements^2^. As one of the most accessible and popular dietary supplements for athletes and exercising individuals, the safety and efficacy of exogenous creatine in sports or exercise have been extensively studied^3^. However, the creatine biology in pathological conditions remains poorly understood. Recent advances have revealed the regulatory roles of creatine in various pathophysiological scenarios, including (1) brown or beige adipocyte thermogenesis against obesity^4^, (2) macrophage polarization in antibacterial host defense^5^, (3) T cell capacity in antitumor immunity^6^, and importantly, (4) tumor cell dissemination^7^. Thus, regulation of creatine metabolism may hold therapeutic potential for various diseases, and understanding the regulatory mechanism underlying different diseases is crucial for therapy development. Although exogenous creatine has been tested in cancer patients^8^, its impact on tumors remains controversial. Recent studies suggest that exogenous creatine promotes cancer metastasis via direct upregulation of tumor cell motility^7,9,10^. However, the role of exogenous creatine in various genetically healthy host cells, which are deeply involved in the cancer metastasis cascade, has been largely overlooked.

The cancer metastasis cascade is a multi-step process involving invasion, intravasation, circulation, extravasation, and colonization. Once cancer cells enter the bloodstream, platelets support circulating tumor cells (CTCs) by overcoming the blood shear forces, shielding against the immune cell surveillance, preventing anoikis, and facilitating CTC extravasation into the distal organs^11–14^. Clinical evidence shows that thrombocytosis is inversely associated with poor prognosis of cancer, whereas antithrombotic drugs improve survival in patients with cancer^15,16^. Platelets are derived from megakaryocytes (MKs), which are morphologically distinct (30–100 μm in diameter), polyploid, and hypermetabolic to meet the constant demand for platelet production^17^. Although perturbations in metabolic pathways of MKs are thought to predetermine platelet function, the MK metabolic paradigms and their pathophysiological consequences are far from clear. Our group has revealed the roles of glucose metabolism and ketone body metabolism in MKs and in platelet function^18,19^. However, to the best of our knowledge, no study has explored the role of creatine in platelets or MKs.

The signal transducer and activator of transcription (STAT) pathways are critical for many cellular functions. Among its family members 1–6, STAT5, including two paralogs STAT5A and STAT5B, is linked to hematopoiesis, particularly lymphocyte development, where Janus kinase (JAK) phosphorylates STAT5A/B and induces a conformational change to form STAT5A/B dimers for initiating downstream gene transcription^20,21^. Recent studies reveal that STAT5B, although structurally similar to STAT5A, is not functionally redundant. STAT5B is modified by post-translational modifications, including phosphorylation or SUMOylation at additional sites^20^. Clinically, mutations of *STAT5B* are often associated with immunodeficiency^22^. Notably, most research has focused on the role of STAT5B in lymphocytes and leukemias^23^. Although STAT5B is expressed in MKs and even at higher levels than STAT5A^20^, its role in MK and platelet function remains poorly understood.

In this study, we discovered that exogenous creatine markedly induced hyperactive platelets, which consequently supported CTCs for metastasis. Creatine supplementation increased MK creatine levels and activated creatine kinase B (CKB). CKB phosphorylated STAT5B at a noncanonical site for nuclear trafficking and downstream transcription of various platelet function-related genes. Conversely, MK-specific knockout of the creatine transporter, MK-specific knockout of *Stat5b*, or pharmacological inhibition of STAT5 ablated creatine-augmented hyperactive platelets and prevented creatine-induced metastasis. In human studies, we showed that creatine supplementation in healthy volunteers results in hyperactive peripheral platelets that are sufficient to promote tumor metastasis in an adoptive platelet transfer model in immunocompromised mice. Our findings reveal the role of creatine in driving platelet hyperactivity, which may establish an anti-metastatic therapeutic paradigm via targeting megakaryocyte metabolism.

## Results

### Exogenous creatine promotes platelet-dependent tumor metastasis in mice

**Fig. 1:**
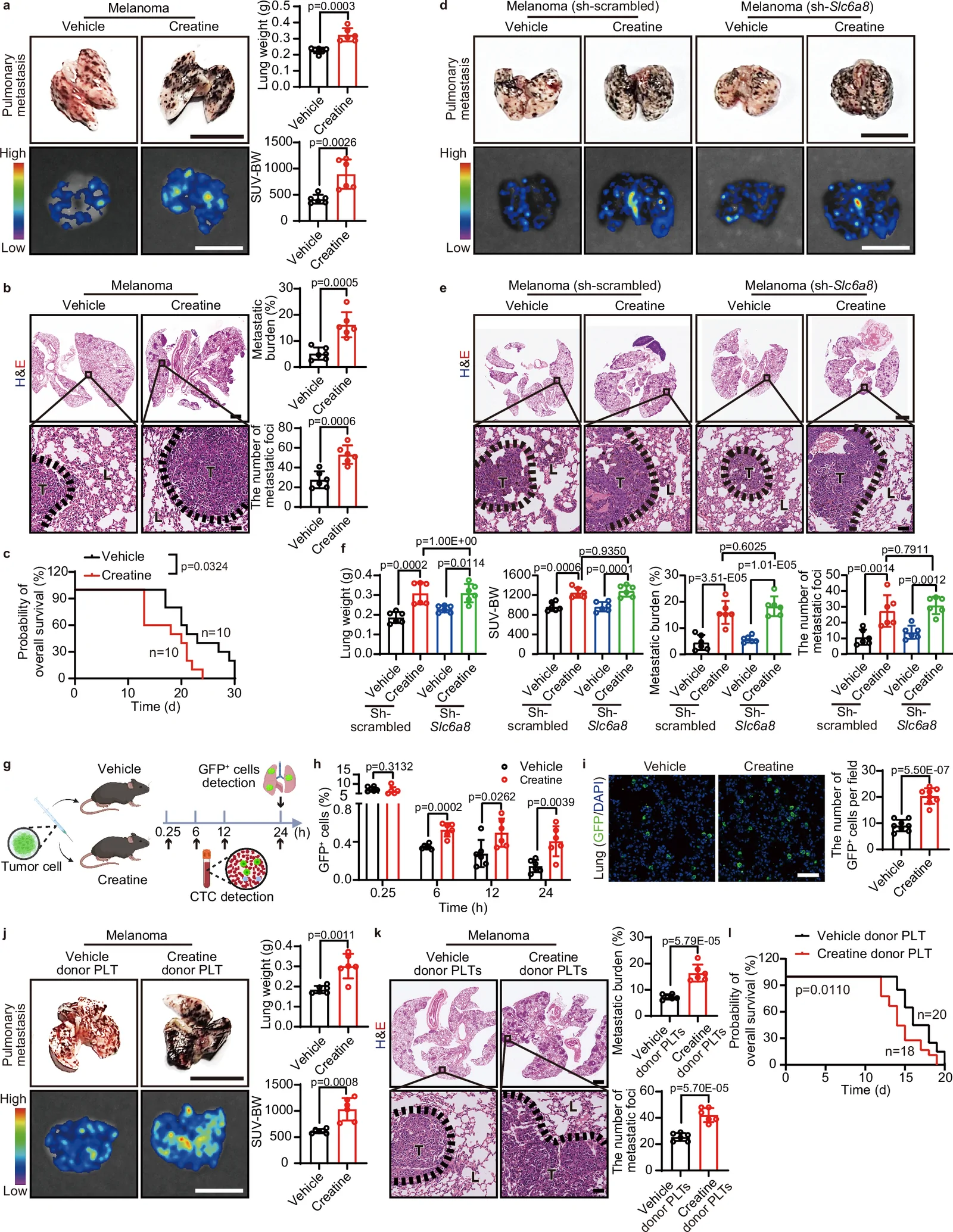
Exogenous creatine promotes platelet-dependent tumor metastasis in mice. **a**–**f** Mice were treated with vehicle or creatine for 7 days and were then injected i.v. with B16-F10-GFP melanoma cells (**a**–**c**). Mice were treated with vehicle or creatine for 7 days and were then injected i.v. with melanoma cells transfected with sh-scrambled or sh-*Slc6a8* (**d**–**f**). Representative lung pictures with visible metastatic nodules. Representative fluorescent micrographs of lungs. Quantifications of lung weight and fluorescent signals (*n* = 6 mice per group). Scale bar in upper and lower panels, 1 cm. Hematoxylin and eosin (H&E) histological analysis of lung metastasis. Dashed lines mark the borders between tumor (T) and lung (L) tissues. Scale bar in upper panels, 2 mm. Scale bar in lower panels, 50 μm. Quantifications of metastatic burden and the number of metastatic foci per lung (*n* = 6 mice per group) (**a**, **b**,** e**,** f**). Overall survival of vehicle- or creatine-treated mice injected with melanoma tumor cells (*n* = 10 mice per group) (**c**). **g**–**i** Schematic model (created in BioRender. Ruibo, C. (2026) https://BioRender.com/6d8e3v9) and quantification of GFP^+^ tumor cells from blood of vehicle- or creatine-treated mice at various timepoints (*n* = 6 mice per group) (**g**, **h**), and from lung after 24 h of i.v. injection (*n* = 8 random fields per group). Scale bar, 50 μm (**i**). **j–l** Platelets were isolated from vehicle- or creatine-treated donor mice and were co-injected with melanoma cells into healthy recipient mice. Representative lung pictures, fluorescent micrographs, and quantifications of lung weight and fluorescent signals (*n* = 6 mice per group). Scale bar in upper and lower panels, 1 cm. H&E histological analysis and quantifications of lung metastasis (*n* = 6 mice per group). Scale bar in upper panels, 2 mm. Scale bar in lower panels, 50 μm. Overall survival of two recipient groups (*n* = 18 or 20 mice per group). Statistical significance in (**a**, **b**, **h**–**k**) was determined by unpaired two-tailed Student’s *t* test. Statistical significance in (**c**, **l**) was determined by log-rank test. Statistical significance in (**f**) was determined by one-way ANOVA. Source data are provided as a Source data file. Data presented as mean ± s.d.

To elucidate the role of creatine in cancer metastasis, we focused on CTCs, which are important for “seed” dissemination in the metastasis cascade. Murine metastatic melanoma cells were stably transfected with enhanced GFP for metastasis foci visualization. To mimic the creatine supplementation in humans, exogenous creatine was dissolved in drinking water and was orally administrated to healthy C57BL/6 male mice at a clinically relevant dose^7^. After 1 week of administration, overall calorie and water intake were unaffected, and the mouse bodyweight showed an insignificant trend towards an increase in the creatine-treated group (Supplementary Fig. 1a–c). On day 7, circulating creatine in the peripheral blood from creatine-treated mice showed a 3-fold increase to approximately 200 μM (Supplementary Fig. 1d), similar to previously published results in mice and humans^6,24^. Tumor cells were then injected into the tail vein to mimic CTCs. Interestingly, ex vivo visualization and histological examination revealed a significant increase in the number and size of pulmonary metastatic foci (Fig. 1a, b). To generalize these findings, murine colorectal cancer (CRC) cells or Lewis lung carcinoma cells were i.v. injected in the same settings, and the results suggested that exogenous creatine promotes pulmonary metastasis (Supplementary Fig. 1e, f). Similarly, creatine promoted breast cancer metastasis in female BALB/c mice (Supplementary Fig. 1g), indicating no sex difference in creatine-induced metastasis. In addition to oral administration of creatine, we also applied a creatine-rich diet and its control diet designed to provide the same calorie intake in mice^7^. A week of dietary uptake of creatine resulted in circulating creatine levels of more than 200 μM, and significantly promoted melanoma pulmonary metastasis (Supplementary Fig. 1h, i). In a spontaneous metastasis model, creatine was administered to tumor-bearing mice once tumors reached 0.5 cm^3^. Again, creatine administration was found to significantly promote melanoma pulmonary metastasis (Supplementary Fig. 1j, k). To study the consequence of the creatine-elevated metastasis rate, we performed a survival analysis in this model. Consistently, the survival of creatine-treated melanoma-bearing mice was markedly shortened (Fig. 1c). These results indicate that exogenous creatine promotes metastasis of infused tumor cells in various mouse models and in both sexes.

Exogenous creatine was taken up and transported into target cells via the creatine transporter SLC6A8. By stable knockdown of *Slc6a8* in tumor cells using shRNA (Supplementary Fig. 2a), we surprisingly found that loss-of-SLC6A8 in tumor cells did not block creatine-elevated metastasis (Fig. 1d). Detailed gross and histological detection showed that *Slc6a8* knockdown in tumor cells did not alter the number of visible and microscopic pulmonary metastatic nodules (Fig. 1e, f). To understand the creatine-induced SLC6A8-independent metastasis, we performed a detailed time-point analysis of infused tumor cells. Similar to previously published studies^25^, the proportion of infused tumor cells decreased rapidly after entering the circulation. At 15 min after injection, GFP^+^ tumor cells accounted for approximately 6% of circulating nucleated cells. By 24 h, this had decreased to less than 0.1% (Fig. 1g, h, and Supplementary Fig. 2b). Interestingly, in creatine-treated mice, GFP^+^ tumor cells were cleared at a significantly slower rate, resulting in approximately 0.4% by 24 h, 4-fold higher than that in the control group (Fig. 1g, h, and Supplementary Fig. 2b). Consistently, histological analysis revealed that high levels of CTCs led to greater pulmonary accumulation of GFP^+^ tumor cells by 24 h (Fig. 1i). These results suggest that creatine slows the clearance of tumor cells in the circulation.

The infused tumor cells face relentless immune surveillance and shear force stress, while platelets are one of the few factors in the circulation that protect these tumor cells^11,12^. To test whether creatine promotes metastasis via platelets, we used adoptive cell transfer by transplanting freshly isolated platelets from vehicle- or creatine-treated mice into non-treated recipient mice, and recipient mice were simultaneously injected with non-treated tumor cells (Fig. 1j). Surprisingly, platelets from creatine-treated mice markedly promoted metastasis in recipient mice (Fig. 1k). Consequently, adoptive transfer of platelets from creatine-treated mice reduced survival in this model (Fig. 1l). These results suggest that creatine promotes metastasis via platelets.

To study whether creatine-instigated metastasis requires platelet activation, we applied aspirin at a dose of 30 mg/kg to inhibit platelet cyclooxygenase-1 (COX-1)-thromboxane A_2_ (TXA_2_) pathway^26^. We transplanted platelets from the vehicle group, the creatine group, or the creatine + aspirin group into non-treated recipient mice (Supplementary Fig. 2c). Compared with creatine-treated platelets, the same amount of activation-restricted creatine-treated platelets significantly abolished creatine-induced metastasis (Supplementary Fig. 2d), suggesting a platelet activation-dependent mechanism. Together, we show that exogenous creatine promotes metastasis via a platelet-dependent mechanism.

### Creatine induces hyperactive platelets via upregulation of key genes in MKs

**Fig. 2:**
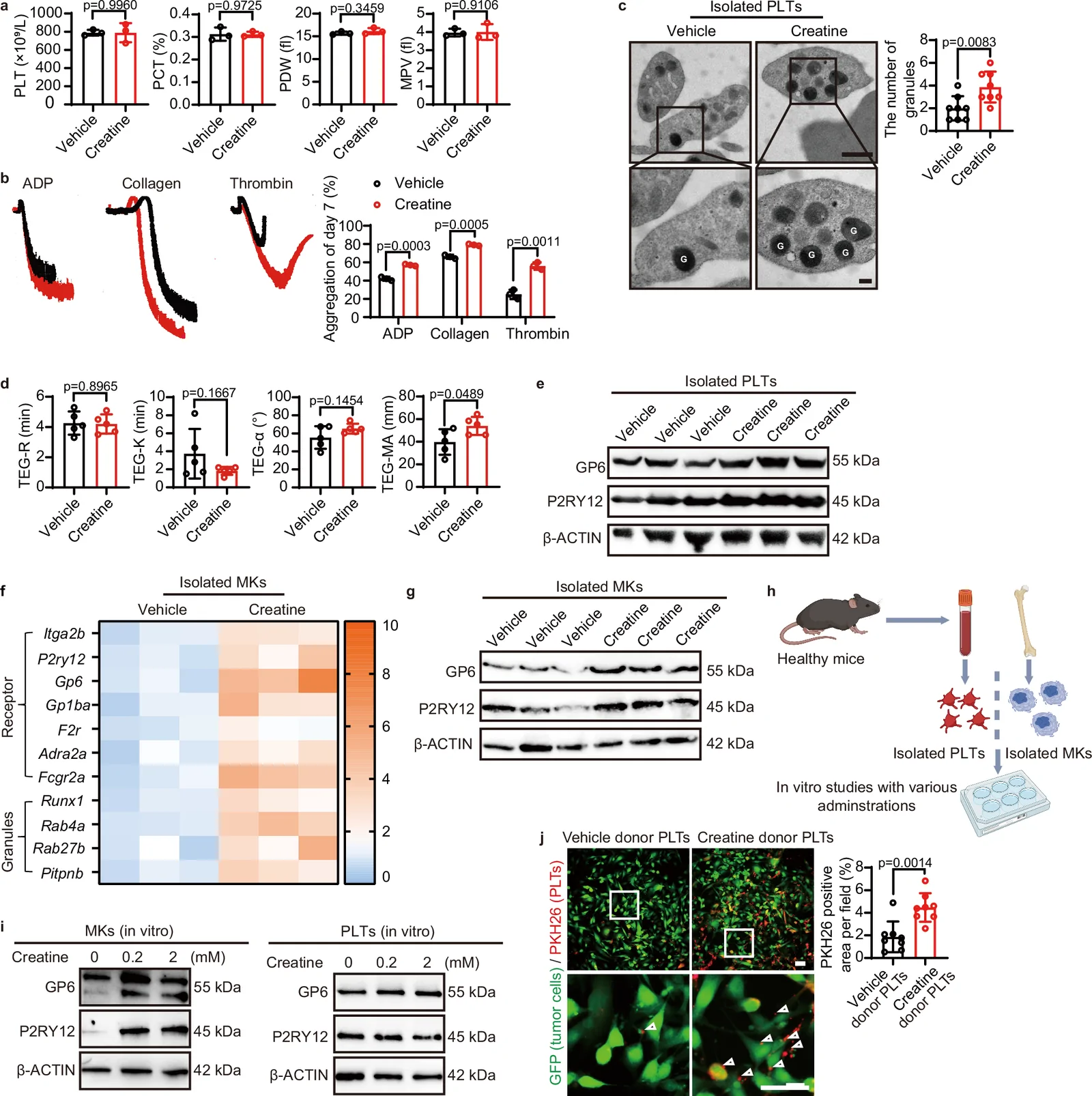
Creatine promotes hyperactive platelets via MK stimulation. **a** Platelet counts (PLT), plateletcrit (PCT), platelet distribution width (PDW), mean platelet volume (MPV) analysis in whole blood from vehicle- or creatine-treated mice (*n* = 3 mice per group). **b** Representative aggregometry tracings of platelet-rich plasma in response to ADP, collagen, or thrombin from vehicle- or creatine-treated mice. Quantification of aggregation (*n* = 3 mice per group). **c** Representative transmission electron microscopy micrographs of platelets isolated from vehicle- or creatine-treated mice. G granule. Scale bar in upper panels, 1 μm. Scale bar in lower panels, 200 nm. Quantifications of granule number per platelet (*n* = 8 random fields per group). **d** Thromboelastographic (TEG) analysis including reaction time (*R*), K time (*K*), α-angle (Angle), and maximum amplitude (MA) in peripheral blood from vehicle- or creatine-treated mice (*n* = 5 mice per group). **e** Western blot of GP6 and P2RY12 in isolated platelets from vehicle- or creatine-treated mice. β-ACTIN marks the loading amount in each lane. **f** Heatmap of platelet receptor- and granule-associated genes of isolated primary MKs from vehicle- or creatine-treated mice (*n* = 3 mice per group). **g** Western blot of GP6 and P2RY12 in isolated primary MKs from vehicle- or creatine-treated mice. β-ACTIN marks the loading amount in each lane. **h** Schematic model of the in vitro study using freshly isolated platelets from peripheral blood or freshly isolated MKs from bone marrow (created in BioRender. Ruibo, C. (2026) https://BioRender.com/a8vew26). **i** Western blot of GP6 and P2RY12 in MKs or platelets treated with or without creatine in vitro. β-ACTIN marks the loading amount in each lane. **j** GFP^+^ tumor cells (green) were co-cultured with PKH26-labeled, freshly isolated platelets (red) from vehicle- or creatine-treated mice. Representative immunofluorescent micrographs. Arrowhead, adhered platelet. Scale bar in upper and lower panels, 40 μm. Quantifications of PKH26^+^ area (*n* = 8 random fields per group). Statistical significance in (**a**–**d**, **j**) was determined by unpaired two-tailed Student’s *t* test. Source data are provided as a Source data file. Data presented as mean ± s.d.

To investigate the biological functions of platelets in creatine-treated mice, peripheral blood samples were collected for platelet detection. Mean platelet volume (MPV), platelet distribution width (PDW), platelet count (PLT), and platelet crit (PCT) were not altered by 7 days of creatine administration (Fig. 2a). Similarly, cell counts of white blood cells, lymphocytes, monocytes, and red blood cells were not altered (Supplementary Fig. 3a). However, freshly isolated platelets were more sensitive to activators ADP, collagen, and thrombin, as tested by aggregometry (Fig. 2b). Transmission electron microscopy (TEM) confirmed that platelet size was not altered but interestingly, granule numbers significantly increased to approximately 2-fold (Fig. 2c). Whole blood thromboelastogram revealed that coagulation factors and fibrinogen remained unchanged while platelet aggregation was significantly increased as evidenced by increased maximum amplitude (MA) (Fig. 2d). These results suggest the presence of hyperactive platelets in creatine-treated mice.

To understand the mechanism of creatine-induced hyperactive platelets, we examined the expression of key receptors in freshly isolated platelets. Interestingly, glycoprotein VI (GP6) and purinergic receptor P2Y12 (P2RY12) were strongly upregulated in the platelets from creatine-treated mice (Fig. 2e). We further isolated bone marrow MKs using a density gradient centrifugation-based method^19^ and validated cell identity by FACS (Supplementary Fig. 3b). The mRNA levels of genes associated with platelet receptors and granules were detected by qPCR and the protein levels were measured by Western blot. RNA expression of platelet receptors, including *integrin subunit alpha 2b* (*Itga2b*), *purinergic receptor P2Y12* (*P2ry12*), *glycoprotein VI* (*Gp6*), *glycoprotein Ib platelet subunit alpha* (*Gp1ba*), *coagulation factor II thrombin receptor* (*F2r*), *adrenoceptor alpha 2A* (*Adra2a*), and *Fc gamma receptor IIa* (*Fcgr2a*), was markedly upregulated upon creatine administration. Likewise, RNA expression of granule-associated genes, including *RUNX family transcription factor 1* (*Runx1*), *RAB4A, member RAS oncogene family* (*Rab4a*), *RAB27B, member RAS oncogene family* (*Rab27b*), and *phosphatidylinositol transfer protein, beta* (*Pitpnb*), showed similar increases (Fig. 2f). Western blot confirmed the upregulation of receptors in MKs (Fig. 2g), suggesting that creatine-induced hyperactive platelets originate from creatine-treated MKs. To validate this view, freshly isolated MKs and platelets were stimulated with creatine in vitro. As expected, creatine-stimulated MKs, but not platelets, responded to creatine at a clinically relevant dose of 200 μM (Fig. 2h, i). In contrast to platelet activation-associated genes, platelet production-associated genes remained unaltered in MKs from creatine-treated mice (Supplementary Fig. 3c), supporting the findings that creatine insignificantly affects platelet count (Fig. 2a). To test the effect of these hyperactive platelets on tumor cells, freshly isolated platelets were labeled with a fluorescent dye and added onto cultured tumor cells. After washing, platelets from creatine-treated mice were significantly more adherent to tumor cells, suggesting that creatine promotes the formation of platelet-tumor cell clusters (Fig. 2j). Together, these results suggest that creatine induces hyperactive, tumor cell-adherent platelets via upregulation of various platelet-activation-associated genes in MKs.

### MK-specific *Slc6a8* depletion blocks creatine-induced platelet hyperactivity and metastasis

**Fig. 3:**
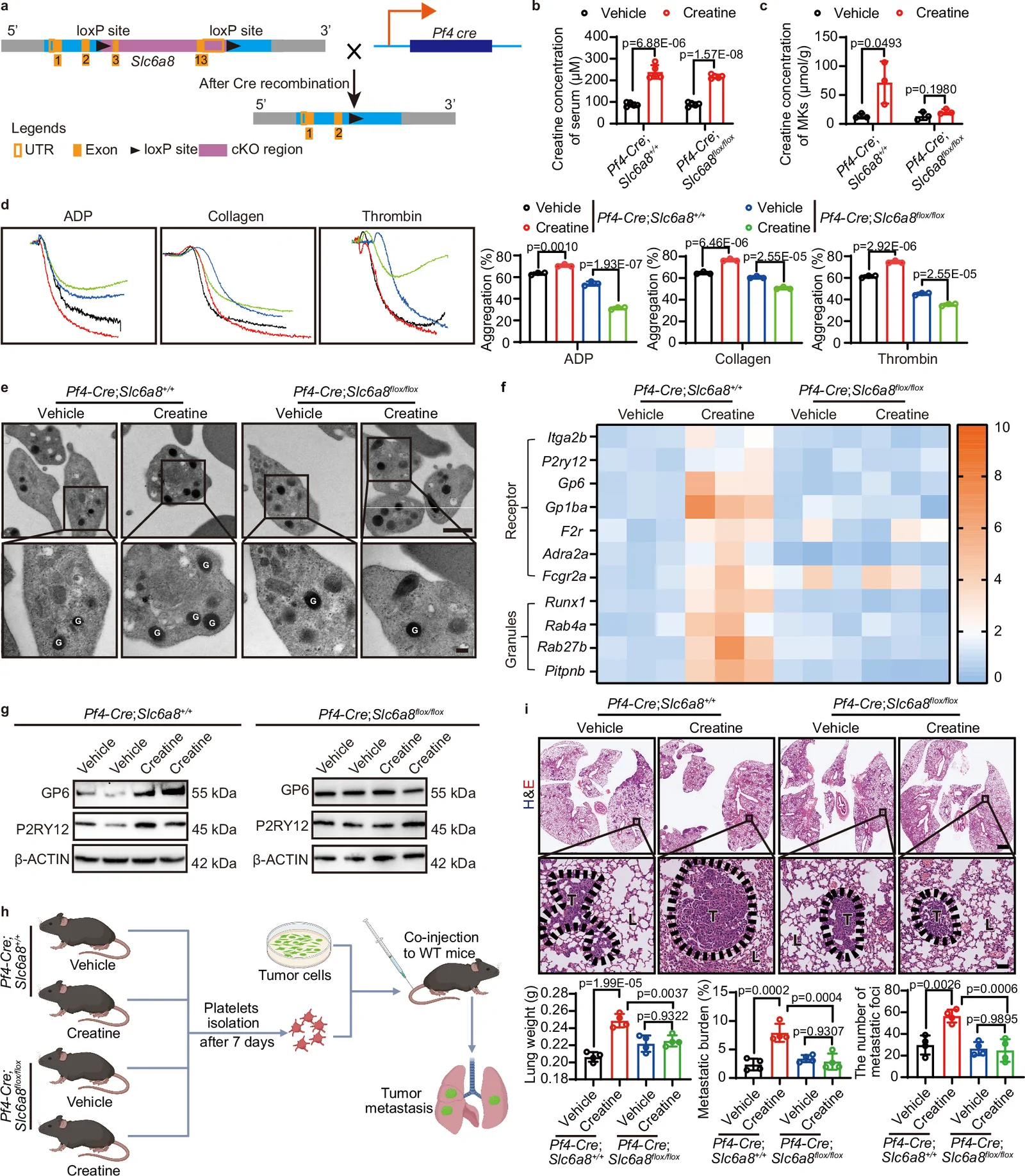
Genetic depletion of *Slc6a8* ablates creatine-induced platelet hyperactivity and metastasis. **a** Generation of *Pf4 Cre Slc6a8*^*flox/flox*^ mice. Mice expressing Cre under a cell type-specific *Pf4* promoter were crossbred with mice with loxP sites inserted into the *Slc6a8* region. MK-specific *Slc6a8* KO mice were identified by genotyping. UTR, untranslated region. **b** Circulating creatine concentrations in vehicle- or creatine-treated wt or *Pf4 Cre Slc6a8*^*flox/flox*^ mice (*n* = 5 mice per group). **c** Intracellular creatine concentrations in freshly isolated MKs from vehicle- or creatine-treated wt or *Pf4 Cre Slc6a8*^*flox/flox*^ mice (*n* = 3 mice per group). **d** Representative aggregometry tracings of platelet-rich plasma in response to ADP, collagen, or thrombin from vehicle- or creatine-treated wt or *Pf4 Cre Slc6a8*^*flox/flox*^ mice. Quantification of aggregation (*n* = 3 mice per group). **e** Representative transmission electron microscopy micrographs of platelets isolated from vehicle- or creatine-treated wt or *Pf4 Cre Slc6a8*^*flox/flox*^ mice. G, granule. Scale bar in upper panels, 1 μm. Scale bar in lower panels, 200 nm. **f** Heatmap of platelet receptor- and granule-associated genes of isolated primary MKs from vehicle- or creatine-treated wt or *Pf4 Cre Slc6a8*^*flox/flox*^ mice (*n* = 3 mice per group). **g** Western blot of GP6 and P2RY12 in isolated primary MKs from vehicle- or creatine-treated wt or *Pf4 Cre Slc6a8*^*flox/flox*^ mice. β-ACTIN marks the loading amount in each lane. **h** Schematic diagram of the adoptive platelet transfer model. Platelets were isolated from vehicle- or creatine-treated wt or *Pf4 Cre Slc6a8*^*flox/flox*^ donor mice and were co-injected with melanoma cells into healthy recipient mice (created in BioRender. Ruibo, C. (2026) https://BioRender.com/hkklgl7). **i** H&E histological analysis of lung metastasis. Dashed lines mark the borders between tumor (T) and lung (L) tissues. Scale bar in upper panels, 2 mm. Scale bar in lower panels, 50 μm. Quantifications of metastatic burden and the number of metastatic foci (*n* = 4 mice per group). Statistical significance in (**b**, **c**) was determined by unpaired two-tailed Student’s *t* test. Statistical significance in (**d**, **i**) was determined by one-way ANOVA. Source data are provided as a Source data file. Data presented as mean ± s.d.

Knowing that creatine directly acts on MKs for hyperactive platelets, we next studied the role of the MK creatine transporter using a genetic approach to delete *Slc6a8* specifically in MKs by cross-breeding *Pf4 Cre* mice with *Slc6a8*^*flox/flox*^ mice (Fig. 3a). This approach effectively deleted SLC6A8 expression in isolated primary MKs (Supplementary Fig. 4a). Platelet counts were not altered by SLC6A8 depletion with or without creatine administration (Supplementary Fig. 4b). After 7 days of creatine administration, although circulating creatine levels were not affected in MK-specific creatine transporter knockout mice (Fig. 3b), the intra-MK creatine concentration was abolished (Fig. 3c). Functionally, the creatine-induced platelet hyperactivity to ADP, collagen, and thrombin was abolished by MK-specific *Slc6a8* depletion (Fig. 3d). Platelet morphology remained unaltered, and the creatine-induced increase in granules was abolished by this genetic approach (Fig. 3e and Supplementary Fig. 4c). In freshly isolated MKs, the creatine-stimulated gene expression profile and protein expression were markedly inhibited in *Pf4 Cre*; *Slc6a8*^*flox/flox*^ mice (Fig. 3f, g). These findings demonstrate that the MK creatine transporter is critical for creatine-induced platelet hyperactivity.

To validate whether loss of platelet hyperactivity reduces the metastasis rate of infused tumor cells, we isolated platelets from vehicle- or creatine-treated *Pf4 Cre*; *Slc6a8*^*+/+*^ mice and *Pf4 Cre*; *Slc6a8*^*flox/flox*^mice for the aforementioned adoptive platelet transfer model (Fig. 3h). As expected, transplantation of creatine-pretreated platelets from *Pf4 Cre*; *Slc6a8*^*+/+*^ mice markedly promoted metastasis compared to the vehicle-treated group (Fig. 3i). Transplantation of creatine-pretreated platelets from *Pf4 Cre*; *Slc6a8*^*flox/flox*^mice completely abolished the creatine-associated metastasis-promoting effect (Fig. 3i). We next performed the rescue experiment by delivering creatine bypassing the transporter in *Slc6a8*^*-/-*^ MKs isolated from *Pf4 Cre*; *Slc6a8*^*flox/flox*^mice. Administration with 2 mM creatine in vitro on these MKs did not increase the expression of platelet-associated genes. In contrast, administration with liposomes containing creatine (creatine@liposome) elevated the expression of platelet-associated genes in creatine transporter-depleted MKs in vitro (Supplementary Fig. 4d, e). Using genetically engineered mouse models, we provide compelling evidence that exogenous creatine induces platelet hyperactivity and metastasis via the creatine transporter in MKs.

### Creatine-induced platelet hyperactivity depends on CKB upregulation

**Fig. 4:**
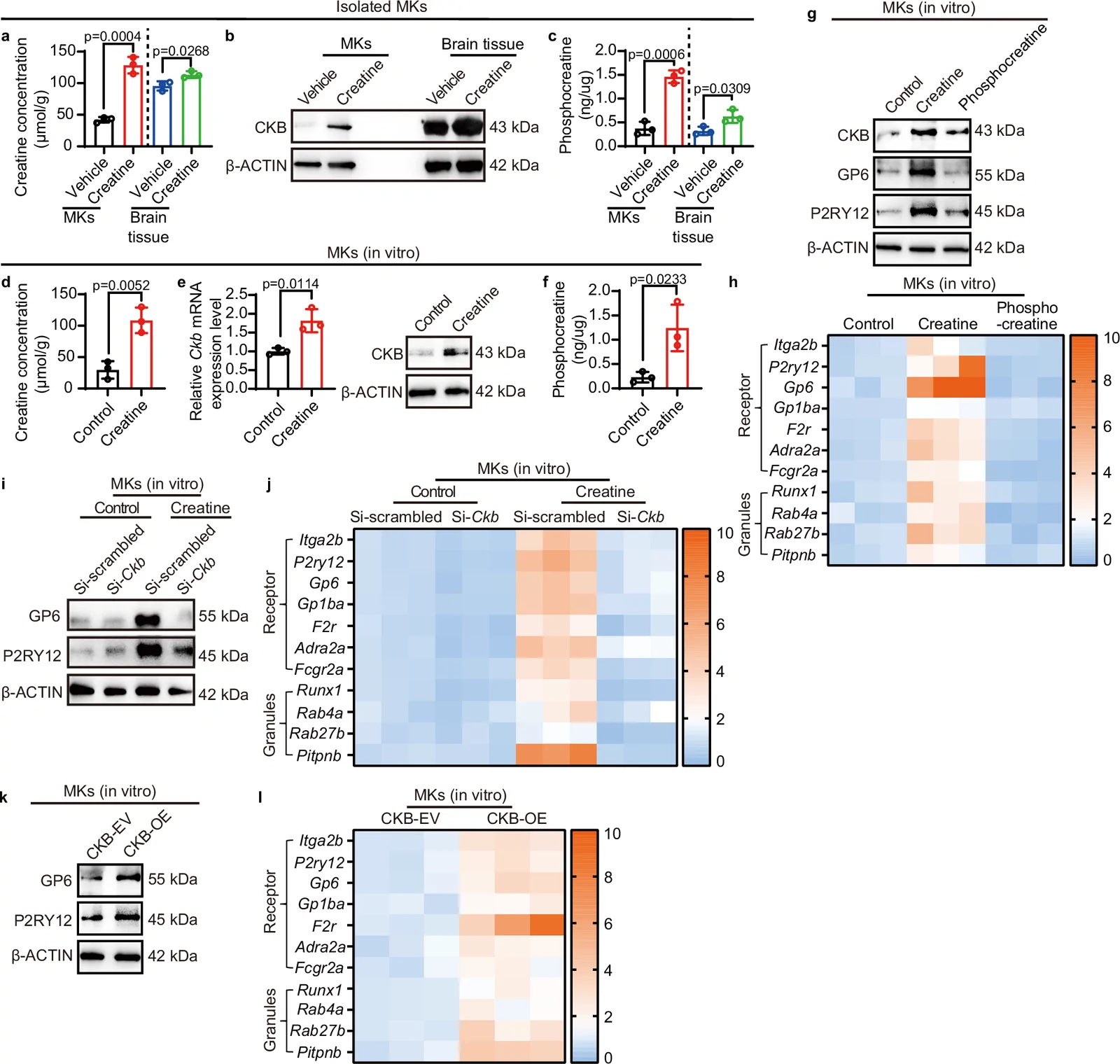
Creatine promotes CKB expression for platelet hyperactivity in MKs. **a** Intracellular creatine concentrations in freshly isolated MKs from vehicle- or creatine-treated mice (*n* = 3 mice per group). Brain tissues from the same group served as controls. **b** Western blot of CKB in freshly isolated MKs from vehicle- or creatine-treated mice. Brain tissues from the same group served as controls. β-ACTIN marks the loading amount in each lane. **c** Intracellular phosphocreatine concentrations in freshly isolated MKs from vehicle- or creatine-treated mice (*n* = 3 mice per group). **d** Intracellular creatine concentrations in MKs treated with or without creatine in vitro (*n* = 3 samples per group). **e** QPCR quantification of *Ckb* mRNA expression in MKs treated with or without creatine in vitro (*n* = 3 samples per group). Western blot of CKB in MKs treated with or without creatine in vitro. β-ACTIN marks the loading amount in each lane. **f** Intracellular phosphocreatine concentrations in MKs treated with or without creatine in vitro (*n* = 3 samples per group). **g** Western blot of CKB, GP6, and P2RY12 in MKs treated with or without creatine or phosphocreatine in vitro. β-ACTIN marks the loading amount in each lane. **h** Heatmap of platelet receptor- and granule-associated genes in MKs treated with or without creatine or phosphocreatine in vitro (*n* = 3 samples per group). **i** Western blot of GP6 and P2RY12 in si-scrambled- or si-*Ckb*-transfected MKs treated with or without creatine in vitro. β-ACTIN marks the loading amount in each lane. **j** Heatmap of platelet receptor- and granule-associated genes in si-scrambled- or si-*Ckb*-transfected MKs treated with or without creatine in vitro (*n* = 3 samples per group). **k** Western blot of GP6 and P2RY12 in empty vector- or *Ckb* overexpression vector-transfected MKs in vitro. β-ACTIN marks the loading amount in each lane. **l** Heatmap of platelet receptor- and granule-associated genes in empty vector- or *Ckb* overexpression vector-transfected MKs in vitro (*n* = 3 samples per group). Statistical significance in (**a**, **c**–**f**) was determined by unpaired two-tailed Student’s *t* test. Source data are provided as a Source data file. Data presented as mean ± s.d.

To further investigate the mechanism of creatine-induced hyperactive platelets in MKs, we examined the creatine energy buffering process in which creatine and ATP are catalyzed by creatine kinases to form phosphocreatine and ADP^27^. In isolated MKs from creatine-treated mice, creatine levels, CKB levels, and phosphocreatine levels were significantly upregulated (Fig. 4a–c) as previously reported^28^. Interestingly, creatine levels in brain tissue, which is known to synthesize creatine and express high levels of CKB, were relatively independent of exogenous creatine. In contrast, creatine levels in bone marrow MKs were strongly affected by creatine supplementation and exhibited similar levels of intracellular creatine and CKB expression compared with brain tissues (Fig. 4a–c). Of note, exogenous creatine supplementation did not alter the key enzymes of de novo creatine synthesis in MKs (Supplementary Fig. 5a). In MKs administrated with creatine in vitro, intracellular creatine levels, CKB levels, and phosphocreatine levels were similarly upregulated (Fig. 4d–f). To study whether the pro-hyperactive platelet effect is instigated by high energy supply, we administered phosphocreatine to MKs in vitro. Surprisingly, in contrast to the creatine-induced effect, we did not observe elevated CKB levels or increased protein or RNA levels of platelet function genes (Fig. 4g, h), suggesting that the upregulation of these genes is independent of energy metabolism.

We therefore focused on the increased expression of CKB in MKs. Importantly, *Ckb* siRNA significantly blocked the creatine-stimulated gene expression profile and protein expression (Fig. 4i, j, and Supplementary Fig. 5b). Furthermore, overexpression of CKB without creatine supplementation was sufficient to instigate RNA and protein expression of platelet function genes (Fig. 4k, l, and Supplementary Fig. 5b), suggesting a role for CKB in regulating gene expression in MKs. Notably, overexpression of CKB had no significant effect on either creatine levels or phosphocreatine levels in MKs (Supplementary Fig. 5c). Together, these results suggest that creatine-induced platelet hyperactivity is dependent on the upregulation of CKB in MKs. In addition to maintaining the cellular ATP pool, CKB was recently found to have protein kinase activity^29,30^. These findings led us to investigate the downstream pathways of CKB-associated signaling in MKs.

### Creatine induces noncanonical STAT5B phosphorylation in MKs

**Fig. 5:**
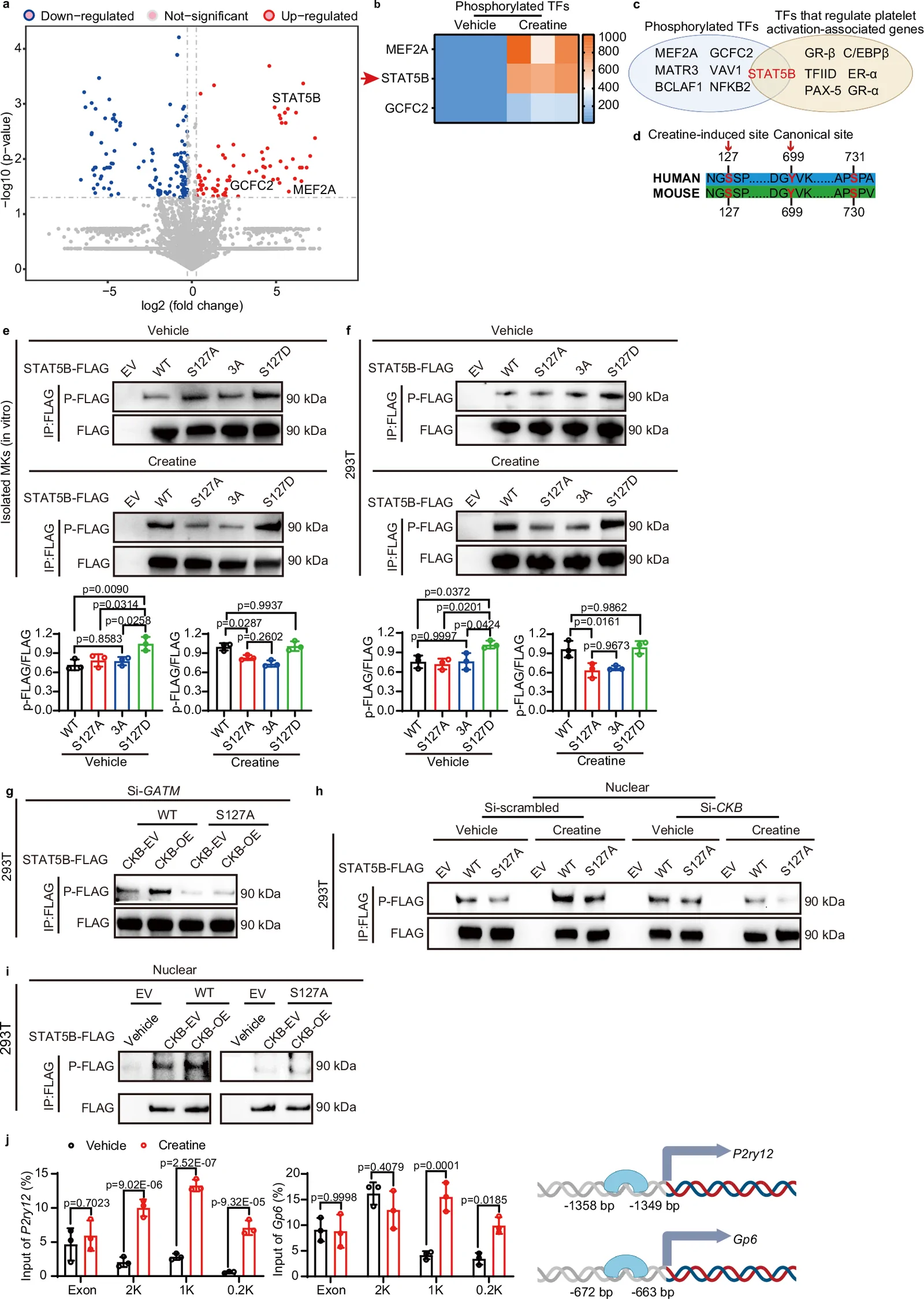
Noncanonical STAT5B phosphorylation confers creatine-instigated gene expression in MKs. **a** Volcano plot of 5812 differentially phosphorylated peptides in freshly isolated MKs from vehicle- or creatine-treated mice (*n* = 3 mice per group). **b** Top 3 phosphorylated TFs in freshly isolated MKs from vehicle- or creatine-treated mice (*n* = 3 mice per group). Arrow points to STAT5B phosphorylated peptides. **c** Venn diagram of top phosphorylated TFs in creatine-treated MKs and predicted TFs that may regulate platelet activation-associated genes. **d** Diagram of human and mouse STAT5B amino acid sequence. Creatine-induced phosphorylation site S127 and canonical phosphorylation sites Y699 and S731 were marked. MKs (**e**) and 293T cells (**f**) were transfected with empty vector and STAT5B-FLAG vector with wt, S127A mutant, triple mutants, or S127D mutant. These groups were then treated with vehicle- or creatine in vitro. Western blot of phosphorylation of STAT5B-FLAG in various groups. FLAG marks the loading amount in each lane. Quantifications of signal in various groups (*n* = 3 blots per group). **g**–**i** SiRNA-*GATM* (human)-transfected 293T cells were further transfected with wt or S127A mutant STAT5B-FLAG vector, and empty vector or *CKB* overexpression vector (**g**). Si-scrambled- or si-*CKB* (human)-transfected 293T cells were further transfected with empty vector, wt STAT5B-FLAG vector, or S127A mutant STAT5B-FLAG vector, and were treated with or without creatine (**h**). 293T cells were transfected with empty vector and STAT5B-FLAG vector with wt or S127A mutant, and further transfected with empty vector or *CKB* overexpression vector (**i**). Western blot of phosphorylation of STAT5B-FLAG in various groups. FLAG marks the loading amount in each lane. **j** ChIP assay of STAT5B binds to different promoter regions of *P2ry12* and *Gp6*. Nonimmune IgG was used for input. Coding region (exon) of each gene served as controls (*n* = 3 samples per group). Schematic diagram of the DNA binding model (created in BioRender. Ruibo, C. (2026) https://BioRender.com/f37rihj). Statistical significance in (**a**) was determined by unpaired two-tailed Student’s *t* test. Statistical significance in (**e**, **f**) was determined by one-way ANOVA. Statistical significance in (**j**) was determined by two-way ANOVA. Source data are provided as a Source data file. Data presented as mean ± s.d.

To investigate the downstream signaling of CKB in creatine-treated MKs, we performed an unbiased phosphoproteomics in freshly isolated MKs from vehicle- and creatine-treated mice. Phosphopeptides were enriched and separated by high-pressure liquid chromatography (HPLC), and subsequently ionized and fast sequenced by phosphorylation-specific multistage MS. A total of 5812 differentially phosphorylated peptides were identified (Fig. 5a and Supplementary Data 1). Because we observed robust and broad mRNA expression in MKs, we focused on differentially phosphorylated transcription factors (TFs) that are likely to bind promoter regions of genes and drive platelet phenotypic changes (Fig. 5b). Interestingly, MEF2A, highly expressed in muscle and associated with muscle development^31^, STAT5B, highly expressed in bone marrow and associated with hematopoiesis and leukemia^20^, and GCFC2, widely expressed and associated with neurological disorders^32^, were ranked as the top 3 up-phosphorylated TFs. We then analyzed the promoter regions of platelet activation-associated genes using the prediction tool PROMO, and found a group of TFs that potentially bind to the promoter region of these genes (Fig. 5c). Intersection of phosphorylated TFs and potential TFs that regulate platelet function genes identified STAT5B as a candidate for creatine-induced platelet hyperactivity. Surprisingly, STAT5B was suggested to be phosphorylated at a previously unknown site S127 in creatine-stimulated MKs (Fig. 5d). In contrast, phosphorylation at the canonical STAT5B phosphorylation site Y699 was not detected by a phospho-STAT5 (Y694/Y699) antibody in either vehicle- or creatine-treated groups (Supplementary Fig. 6a). Molecular docking suggested a direct binding possibility of the CKB enzyme pocket and the site S127, located in the oligomerization domain of STAT5B, compared to that of the canonical site Y699 near the SH2 domain (Supplementary Fig. 6b)^33^. To study whether CKB directly phosphorylates STAT5B, we constructed Flag-STAT5B fusion vectors and transfected these plasmids into 293T cells. Wildtype FLAG-STAT5B was purified from 293T cells and was incubated with bacterially purified wildtype His-CKB in ATP supplemented kinase buffer for 1 h. FLAG-STAT5B co-incubated with CKB showed a strong phosphorylation (Supplementary Fig. 6c, d), validating that CKB directly phosphorylates STAT5B.

To study the detailed mechanism of creatine-induced phosphorylation of STAT5B, we constructed Flag-STAT5B fusion vectors with different mutations and transfected these plasmids into 293T cells and into isolated fresh MKs (Fig. 5e, f). Flag-STAT5B was immunoprecipitated and detected for phosphorylation using a universal phosphorylation antibody. As expected, a STAT5B mutant at site S127 (S127D) mimicking the phosphorylated form was detected by phosphorylation antibody in both vehicle- and creatine-treated cells, whereas a STAT5B phospho-mutant at site S127 (S127A) was not detected by phosphorylation antibody in either vehicle- or creatine-treated cells (Fig. 5e, f). Of note, a triple phospho-mutant at site S127 and two other canonical sites (S127A, Y699A, and S731A, designated 3A) showed that there was no clear additive effect surpassing the S127A mutant. As a result, in both 293T cells and isolated MKs, creatine administration significantly increased wildtype STAT5B phosphorylation, whereas this phosphorylation could be blocked in the S127A mutant group (Fig. 5e, f). Additionally, we blocked the de novo creatine synthesis pathway by *glycine amidinotransferase* (*Gatm*) siRNA, and overexpressed CKB without creatine administration. CKB overexpression alone was sufficient for STAT5B phosphorylation, and S127A abolished this effect (Fig. 5g and Supplementary Fig. 6e, f). These results suggest that CKB promotes noncanonical phosphorylation of STAT5B.

To study whether the STAT5B phosphorylation at site S127 is able to translocate into the nucleus as canonical STAT5B, we detected the nuclear translocation of STAT5B. As expected, creatine administration increased the nuclear localization of phosphorylated STAT5B, whereas CKB knockdown largely abolished this effect. Consistently, CKB overexpression without creatine administration increased the nuclear localization of phosphorylated STAT5B (Fig. 5h, i, and Supplementary Fig. 6g). The nuclear translocation of STAT5B was validated in MKs (Supplementary Fig. 6h). These results suggest that noncanonical phosphorylation of STAT5B promotes its nuclear translocation.

To study the functional consequences of STAT5B nuclear translocation, we performed chromatin immunoprecipitation (ChIP) in isolated MKs from vehicle- or creatine-treated mice. The results showed that STAT5B binds to the promoter region of various genes associated with platelet function, including the receptor-associated genes *P2ry12* and *Gp6*, as well as the granule-associated genes *Rab4a* (Fig. 5j and Supplementary Fig. 6i). Taken together, these results support that creatine initiates gene transcription for platelet hyperactivity via the creatine transporter-CKB-noncanonical STAT5B phosphorylation-nuclear translocation axis.

### Pharmacological inhibition of STAT5 or MK-specific *Stat5b* depletion blocks creatine-induced platelet hyperactivity and metastasis

**Fig. 6:**
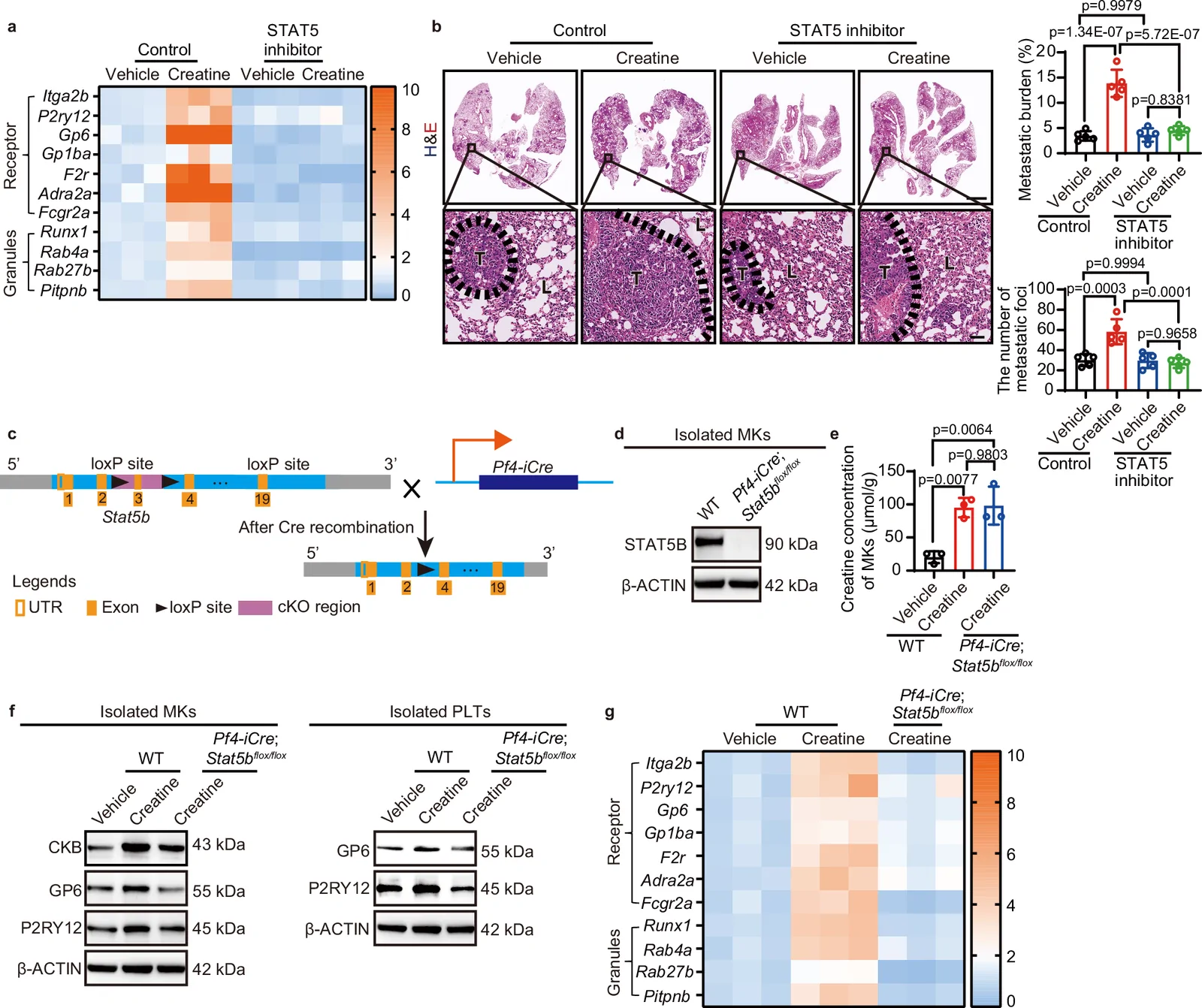
Pharmacological inhibition of STAT5 or MK-specific *Stat5b* depletion blocks creatine-induced platelet hyperactivity and metastasis. **a** Heatmap of platelet receptor- and granule-associated genes of isolated primary MKs from vehicle- or creatine-treated mice with or without STAT5-specific inhibitor administration (*n* = 3 mice per group). **b** H&E histological analysis of lung metastasis. Dashed lines mark the borders between tumor (T) and lung (L) tissues. Scale bar in upper panels, 2 mm. Scale bar in lower panels, 50 μm. Quantifications of metastatic burden and the number of metastatic foci (*n* = 5 mice per group). **c** Generation of *Pf4 iCre Stat5b*^*flox/flox*^ mice. Mice expressing codon-improved Cre recombinase (iCre) under a cell type-specific *Pf4* promoter were crossbred with mice with loxP sites inserted into the *Stat5b* region. MK-specific *Stat5b* KO mice were identified by genotyping. UTR, untranslated region. **d** Western blot of STAT5B in isolated primary MKs from wt or *Pf4 iCre Stat5b*^*flox/flox*^ mice. β-ACTIN marks the loading amount in each lane. **e** Intracellular creatine concentrations in freshly isolated MKs from vehicle- or creatine-treated wt or *Pf4 iCre Stat5b*^*flox/flox*^ mice (*n* = 3 samples per group). **f** Western blot of CKB, GP6, and P2RY12 in isolated primary MKs and isolated platelets from vehicle- or creatine-treated wt or *Pf4 iCre Stat5b*^*flox/flox*^ mice. β-ACTIN marks the loading amount in each lane. **g** Heatmap of platelet receptor- and granule-associated genes of isolated primary MKs from vehicle- or creatine-treated wt or *Pf4 iCre Stat5b*^*flox/flox*^ mice. (*n* = 3 mice per group). Statistical significance in (**b**, **e**) was determined by one-way ANOVA. Source data are provided as a Source data file. Data presented as mean ± s.d.

To test the therapeutic potential of targeting STAT5B for treating hyperactive platelets, we applied STAT5-IN-1, a STAT5 specific inhibitor^34^, for in vivo administration. Daily administration of STAT5 inhibitor in mice did not alter platelet size or number (Supplementary Fig. 7a), but markedly abolished the creatine-elevated expression of platelet function genes (Fig. 6a). Pharmacological inhibition of STAT5 in donor mice markedly abolished metastasis in the platelet-adoptive transfer mouse model (Fig. 6b). These results suggest that targeting STAT5 has therapeutic potential for creatine-induced platelet hyperactivity and metastasis of infused tumor cells.

To further validate the role of STAT5B in MKs, we generated a MK-specific STAT5B KO mice by cross-breeding MK-specific *Pf4 iCre* mice with *Stat5b*^*flox/flox*^ mice (Fig. 6c). The generated genetic modified mouse model breeds normally, with STAT5B effectively depleted in isolated primary MKs (Fig. 6d). Platelet counts were not altered by STAT5B depletion with or without creatine administration (Supplementary Fig. 7b). Intracellular creatine levels were not altered (Fig. 6e), and the CKB levels remained at high level (Fig. 6f). However, in freshly isolated MKs, the creatine-stimulated gene expression profile and protein expression was markedly inhibited in *Pf4 iCre*; *Stat5b*^*flox/flox*^ mice (Fig. 6f, g). Taken together, these findings demonstrate that creatine-induced platelet hyperactivity is MK STAT5B dependent.

### Creatine supplementation promotes platelet hyperactivity in humans

**Fig. 7:**
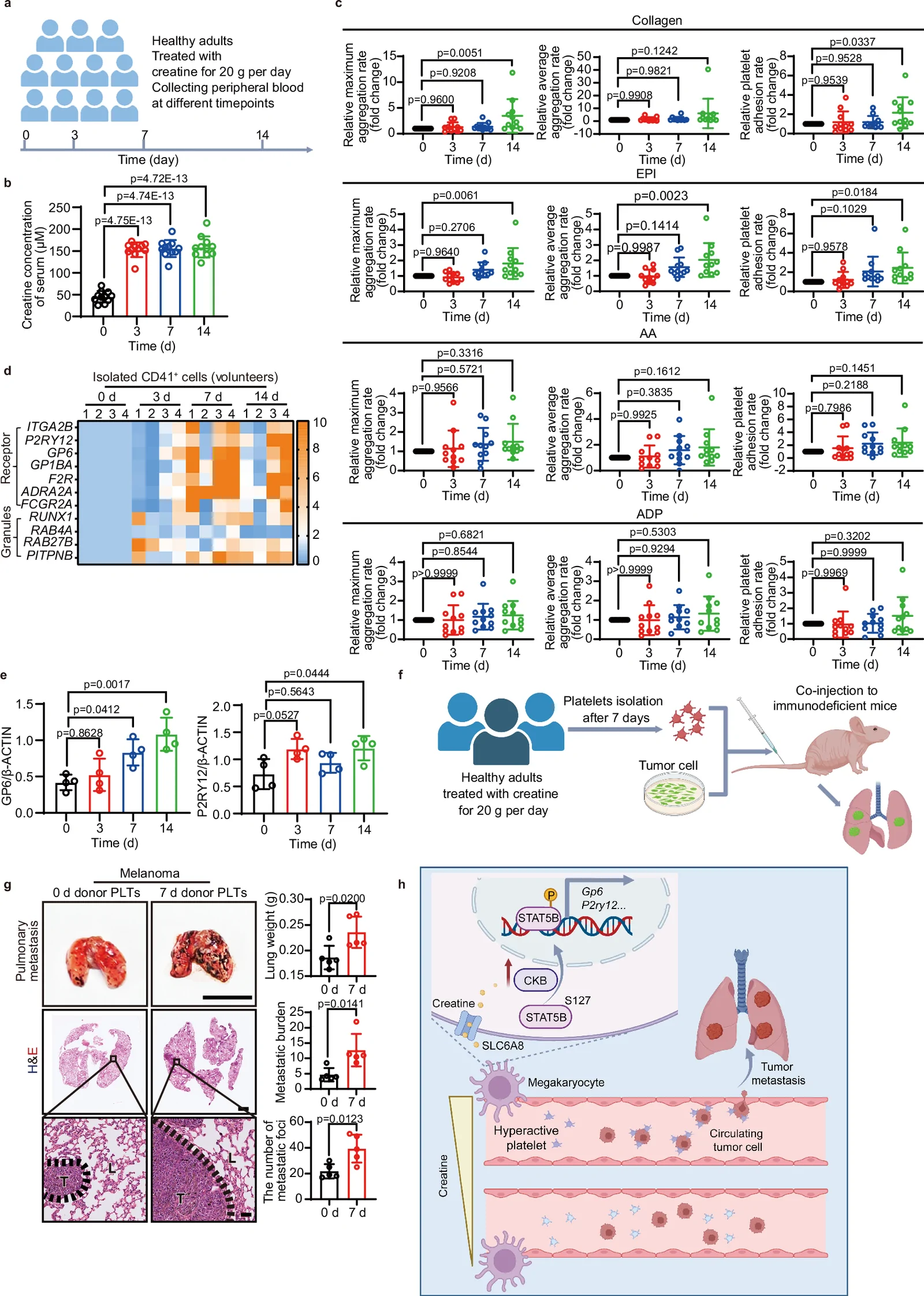
Exogenous creatine supplementation increases hyperactive platelets in humans. **a** Schematic model of 11 healthy male and female volunteers taking 20 g creatine powder per day for 14 days. Peripheral blood samples were collected at various timepoints for further detections. **b** Circulating creatine concentrations at day 0, 3, 7, and 14 after receiving creatine supplement in volunteers (*n* = 11 volunteers per group). **c** Platelet aggregometry analysis of platelet-rich plasma in response to collagen, epinephrine (EPI), arachidonic acid (AA), or ADP. Quantifications of maximum aggregation rate, average aggregation rate, and platelet adhesion rate in various groups (*n* = 11 volunteers per group). **d** Four volunteers were randomly selected, and circulating CD41^+^ cells were isolated. Heatmap of mRNA expression of platelet receptor- and granule-associated genes in isolated CD41^+^ cells at various timepoints (*n* = 4 volunteers per group). **e** Quantification of protein levels of GP6 and P2RY12 in isolated platelets at various timepoints (*n* = 4 volunteers per group). **f**, **g** Schematic diagram of the adoptive platelet transfer model. Platelets were isolated from volunteers before exogenous creatine supplementation and after treatment for 7 days. Donor platelets were co-injected with melanoma cells in immunocompromised recipient mice. Representative lung pictures, H&E histological analysis and quantifications of lung metastasis (*n* = 5 mice per group). Dashed lines mark the borders between tumor (T) and lung (L) tissues. Scale bar in upper panels, 1 cm. Scale bar in middle panels, 2 mm. Scale bar in lower panels, 50 μm. **h** Circulating creatine is taken up by megakaryocytes via the creatine transporter SLC6A8. Elevated intracellular creatine upregulates CKB expression, which in turn phosphorylates STAT5B at the noncanonical site S127. Nuclear-translocated phosphorylated STAT5B drives gene transcription to generate hyperactive platelets. These hyperactive platelets facilitate tumor metastasis by protecting CTCs from blood shear stress, evading immune surveillance, inhibiting anoikis, and promoting CTC extravasation into distant organs. Schematics in (**a**, **f**, **h**) were created in BioRender. Ruibo, C. (2026) https://BioRender.com/35axa7t. Statistical significance in (**b**, **c**, **e**) was determined by one-way ANOVA. Statistical significance in (**g**) was determined by unpaired two-tailed Student’s *t* test. Source data are provided as a Source data file. Data presented as mean ± s.d.

Although creatine supplementation is becoming increasingly popular among exercising individuals, its impact on platelet activity and in tumor metastasis remains unknown. To translate our findings to humans, 11 healthy male and female volunteers were recruited to receive oral creatine supplementation at a dose of 20 g creatine per day without lifestyle changes. Peripheral blood samples were collected on days 0, 3, 7, and 14 (Fig. 7a). Baseline characteristics were summarized, and none of the participants were taking NSAID drugs (Supplementary Table 1). After 14 days of creatine supplementation, BMI of these subjects was unaltered (Supplementary Fig. 8a). Circulating creatine levels were significantly increased to more than 150 μM after day 3 and were kept at this plateau (Fig. 7b). Interestingly, similar to what we observed in mice, platelet count was not altered (Supplementary Fig. 8b) but platelet aggregometry showed a significantly higher activity or a trend towards higher activity of peripheral platelets on day 14 compared to those on day 0, depending on different stimuli (Fig. 7c). To investigate the RNA expression profiles of MKs, we isolated the trace amount of circulating CD41^+^ cells from peripheral blood using magnetic-activated cell sorting. These cells expressed high mRNA levels of human *CD41* (Supplementary Fig. 8c). Consistently, the creatine-stimulated gene expression profile was markedly increased after day 7 of creatine supplementation in 4 randomly selected representative individuals out of the 11 (Fig. 7d). Similarly, human P2RY12 and GP6 were upregulated in isolated platelets in these individuals (Fig. 7e and Supplementary Fig. 8d). These results suggest that creatine supplementation induces human platelet hyperactivity.

### Creatine-induced hyperactive human platelets promote metastasis in mice

To investigate the pro-metastatic effect of these hyperactive human platelets, we established an adoptive platelet xenotransplantation model, in which platelets from volunteers taking creatine supplementation were co-injected with mouse tumor cells in immunodeficient mice (Fig. 7f). Similar to what we observed in mouse models, compared to the same amount of untreated platelets, the creatine-primed human platelets significantly enhanced pulmonary metastasis in immunodeficient mice (Fig. 7g). Histological examination showed that the number of metastatic lesions was significantly increased in the creatine-treated donor platelet group (Fig. 7g). These results support the pro-metastatic effect of creatine-induced hyperactive platelets in humans. Although our human data is preliminary, these results are consistent with our preclinical models and provide the first evidence that creatine supplementation induces hyperactive, pro-metastatic platelets in humans.

## Discussion

Creatine supplementation is commonly used by athletes for increasing muscle mass and function. In recent decades, it is increasingly popular among the health-conscious population and exercising individuals^2^. Creatine can also be used as a medicine to treat neurodegenerative diseases, including Huntington’s disease in the clinic^27,28^. Various reports support the safety of creatine supplementation in healthy adults^35^. However, its role in pathological scenarios has not been fully investigated. Among the major diseases that greatly affect life expectancy, the impact of creatine supplementation in cancer remains elusive. Creatine has been reported to directly inhibit cancer cells or to suppress tumor growth via upregulating cytolytic T cell activity^6^. However, for tumor metastasis, evidence has shown that creatine promotes metastasis in various cancer types by directly assisting cancer cells in migration or colonization^7,9^. Of note, although it is well known that the cancer metastasis cascade is a multistep process involving various tumor-host cell crosstalks, the impact of creatine in host cells during the metastasis cascade has been largely overlooked. We show that creatine stimulates MKs to produce hyperactive platelets that strongly facilitate CTC metastasis. Blocking the creatine-instigated MKs is beneficial for combating tumor metastasis as demonstrated by various gain- and loss-of-function experiments. As creatine exerts both pro-tumor and anti-tumor effects in different cell types, it cannot simply be recommended to patients with cancer. Furthermore, although exercise has been shown to improve outcomes for cancer patients, using exogenous creatine to enhance exercise performance may have the opposite effect. Other than promoting metastasis, platelet hyperactivity is known to be associated with thrombotic events^36^. This interesting point requires further investigation.

Our work reveals the previously unknown effects of exogenous creatine on MKs. Although platelets express creatine transporter, they do not respond directly to creatine in vitro. In contrast, MKs absorb creatine via SLC6A8 and increase CKB expression for platelet hyperactivity (Fig. 7h). This evidence demonstrates different creatine response paradigms between platelets and MKs. Compared to cytokines and ligands that directly activate platelets, metabolites as signals are more easily regulated by dietary intake or global metabolism, making them highly variable. MKs may serve as a signal integration hub to respond to metabolite signals and thereby alter platelet function. This model is supported by the evidence from our previous works^18,19^. One of the important mechanistic discoveries in our present study is that STAT5B is activated by CKB and regulates platelet function. Although it has long been known that STAT5B is more expressed than STAT5A in MKs^20^, its role has only been intensively studied in lymphocyte development and leukemias, but has been largely overlooked in MKs. To our knowledge, this is the first time that STAT5B is reported to regulate platelet function. Some clinical case reports showed that patients with STAT5B mutations develop platelet disorders in addition to lymphocyte disorders^37,38^. It is reasonable to speculate that STAT5B regulates platelet function in certain pathophysiological settings. In addition to CKB, several other kinases are known to phosphorylate STAT5B. Their role in platelet biology requires further investigation.

Cancer metastasis consists of multiple steps, each involving different host cell types that interact with the tumor cells^13,39^. The response to creatine in different cell types is highly variable, as shown by several reports^4–7^. In the circulation, platelets strongly promote CTC metastasis^11,14^, whereas in the primary tumor microenvironment, immune cells and cancer-associated fibroblasts play critical roles in regulating tumor invasion^40^. In distant organs, vascular endothelial cells mediate CTC extravasation^41^. Our work focuses on the circulation microenvironment, where CTC-MK distantly communicate through platelets. The impact of creatine on tumor metastasis should encompass each step of the metastasis cascade and deserves further investigation. In addition, our research focuses on the role of exogenous creatine due to its extensive use and its clinical importance. It should be noted that endogenous creatine can be synthesized by guanidinoacetate N-methyltransferase (GAMT) in the liver, kidney, and pancreas^1,42^ and can be released into the circulation and transported into myocytes and other cell types. Although the GAMT expression in MKs and its biological consequences have not been reported, *Gamt*^*-/-*^ mice are fertile^43^ and *Gamt* RNA expression in bone marrow tissue is generally low in humans^44^, implying that the endogenous creatine may play a minor role in MK biology. The impact of endogenous creatine may differ from that of exogenous creatine supplementation, since the former requires the activation of the creatine biosynthesis pathway and consumes glycine and arginine. The biological consequences of endogenous creatine in MKs remain to be elucidated.

We found that CKB mediates noncanonical phosphorylation of STAT5B. Although molecular docking and in vitro kinase assays show that CKB directly phosphorylates STAT5B, we do not exclude the possibility that other proteins may be involved in the creatine-associated STAT5B phosphorylation process. This phosphorylation site is located at the boundary between the oligomerization domain and the coiled-coil domain. This site is poorly understood, and it is unclear whether it mediates the structural changes of STAT5B and whether it confers dimerization or even tetramerization of STAT5B. Our work has only demonstrated that the noncanonical phosphorylation of STAT5B mediates creatine-induced platelet activation. The real structure of STAT5B and the detailed molecular mechanism require further exploration. In addition, our human study recruited young, healthy participants, whereas cancer predominantly affects elderly people, whose metabolic profiles differ from those of young, healthy adults and who may respond differently to exogenous creatine. Further clinical validation in patients with cancer is warranted.

Together, our results show that creatine supplementation promotes platelet hyperactivity and confers tumor metastasis. Circulating creatine has profound impacts on MKs that drive platelet activation-associated genes via CKB-STAT5B signaling. Creatine-instigated hyperactive platelets further facilitate CTC metastasis in mice. Oral creatine supplementation in humans induces platelet hyperactivity in a small clinical pilot study. This work sheds mechanistic insights into the creatine-induced metastasis risk and provides an anti-metastatic therapeutic paradigm by targeting megakaryocyte creatine metabolism.

## Methods

### Ethical regulations

All animal experiments were approved by the Animal Experimental Ethical Committee of Fudan University, Shanghai, China (20220311-002). All human studies were approved by the Ethical Review Committee in Longyan First Hospital Affiliated to Fujian Medical University (LYREC2023-019-01).

### Cell culture and cell isolation

Murine B16-F10-EGFP melanoma and MC38 colorectal cancer cell lines were purchased (Cat. No. GZQ0031, Shanghai Zhong Qiao Xin Zhou Biotechnology, China; Cat. No. QuiCell-M545, Shanghai QuiCell Biotechnology Co., Ltd. &Technology, China). Murine Lewis lung carcinoma and 4T1 breast cancer cell lines were kindly provided by Prof. Yihai Cao from the Karolinska Institute, Sweden. Human embryonic kidney 293T (HEK 293T) cell line was kindly provided by Prof. Yongbo Wang from Fudan University, China. Human Jurkat T cell line was kindly provided by Prof. Dapeng Yan from Fudan University, China. Human MEG-01 cell line was provided by Dr. Xiangzhong Zhao from the central laboratory, the Affiliated Hospital of Qingdao University, Qingdao, China. Human and mouse platelets were freshly isolated from peripheral blood samples. In brief, acid-citrate-dextrose (ACD) solution was prepared (85 mM sodium citrate (Cat. No. MB2492, Meilunbio, China), 71.38 mM citric acid (Cat. No. MB8820, Meilunbio, China), and 27.78 mM glucose (Cat. No. MB2510, Meilunbio, China) in phosphate-buffered saline (PBS)). Blood samples were collected into ACD-containing tubes with a volume ratio of 6:1. Platelet-rich plasma was made by centrifugation at 200 × *g* for 10 min at 20 °C. Platelets were collected by further centrifugation at 800 × *g* for 2 min at 20 °C. For mouse primary MK isolation, primary MKs were isolated from bone marrow using a mouse MK isolation kit (Cat. No. MAG2014M, TBD Science, China). For human circulating CD41^+^ cells isolation, a magnetic-activated cell sorting system was used (Cat. No. 130-042-201, Miltenyi Biotec). In brief, peripheral blood samples were collected in an anticoagulation tube and centrifuged at 210 × *g* for 5 min. Cells were resuspended in RBC lysis buffer (Cat. No. MA0207, Meilunbio, China) for 5 min to remove RBCs. The single-cell suspension was prepared with a 40 µm filter (Cat. No. 22363547, Fisherbrand) and was stained for 10 min on ice with a rabbit anti-human CD41/ITGA2B antibody (Cat. No. A11836, Abclonal, China; 1:500). After PBS wash, cells were further stained with an Alexa Fluor 647-conjugated donkey anti-rabbit antibody (Cat. No. A31573, Invitrogen; 1:1000). Anti-Alexa Fluor 647 MicroBeads (Cat. No. 130-091-395, Miltenyi Biotec) were subsequently used for magnetic labeling. After washing, positive and negative cells were sorted with an MACS column and magnetic MACS separators (Miltenyi Biotec). CD41^+^ cells were collected for further detections. For cell culture, B16-F10, MC38, LLC, 4T1, and 293T cell lines were cultured in 10% FBS-DMEM (Cat. No. 40130ES76, YEASEN, China; Cat. No. MA0212-1, Meilunbio, China), containing 100 U/mL penicillin, 100 μg/mL streptomycin (Cat. No. MA0110, Meilunbio, China). Jurkat cell line and MEG-01 cell line were cultured in 10% FBS-RPMI 1640 (Cat. No. MA0215, Meilunbio, China). In in vitro experiments testing creatine effect, mouse primary MKs, mouse platelets, and 293T cells were shortly starved in a mixed low-glucose (1.0 g/L) DMEM medium (Cat. No. MA0212-1, Meilunbio, China and Cat. No. MA0581, Meilunbio, China; v/v = 1:3.5). All tumor cell lines were authenticated by short tandem repeat (STR) DNA profiling. All cell lines used in our study were negative for mycoplasma confirmed by a PCR method with 2 primer pairs: forward: 5′-GGCGAATGGGTGAGTAACACG-3′ and reverse: 5′-CGGATAACGCTTGCGACCTATG-3′; forward: 5′-GGGAGCAAACAGGATTAGATACCCT-3′ and reverse: 5′-TGCACCATCTGTCACTCTGTTAACCTC-3′.

### Animals

Male C57BL/6J mice (Cat. No. N000013) and female BALB/cJ (Cat. No. N000020) at the age between 6 and 8 weeks old were purchased from GemPharmatech, China and maintained under a 12-h dark/12-h light cycle with food and water provided ad libitum. To obtain MK-specific *Slc6a8* knockout mice, *Slc6a8*^*flox/flox*^ mice (Cat. No. T019665, GemPharmatech, China) were crossed with *Pf4 Cre* mice (Cat. No. C001050, Cyagen, China) to obtain *Pf4 Cre*; *Slc6a8*^*flox/flox*^ mice. Genotyping was performed using EZ DirectPCR Lysis Reagent (Cat. No. AN11L228, Life-iLab, China) and analyzed by PCR with the following primer pairs: *Slc6a8*^*flox*^ forward: 5′-GCAATTTGCCAAGGTTGTCC-3′ and *Slc6a8*^*flox*^ reverse: 5′-TCTGTCTCTGGCTAAGATTTGCC-3′; *Pf4 Cre* forward: 5′-CCAAGTCCTACTGTTTCTCACTC-3′ and *Pf4 Cre* reverse: 5′-TGCACAGTCAGCAGGTT-3′. MK-specific *Stat5b* knockout mice were established by crossing *Stat5b*^*flox/flox*^ mice (Cat. No. T052232, GemPharmatech, China) with *Pf4-P2A-iCre* mice (Cat. No. T005328, GemPharmatech, China) using the following genotyping primer pairs: *Stat5b*^*flox*^ forward 1: 5′-GACTAGATCCTTTCAACAGTGGGC-3′ and *Stat5b*^*flox*^ reverse 1: 5′-CTGACAAACCAAACAGCTGTCAGG-3′; *Stat5b*^*flox*^ forward 2: 5′-CCATTTGTCAACTGACTCACAAGTG-3′ and *Stat5b*^*flox*^ reverse 2: 5′-CATGTGCACATACATACCCACACAC-3′; *Pf4 iCre* forward: 5′-CTGGTCCCGAAGAAAGCGAT-3′ and *Pf4 iCre* reverse: 5′-CTTCCAGGTGTGTTCAGAGAAGG-3′. The maximal tumor size of the tumor-bearing mice permitted by our ethics committee is <2.5 cm^3^. The maximal tumor size/burden was not exceeded is this study. Mice of either sex were used for metastasis models in this study. The influence of sex was not evaluated in this study.

### Survival assay

Survival studies of tumor-bearing mice were performed at Fudan University, Shanghai, China, according to the ethical permit, in which the humane endpoint (body condition score (BCS) ≤1) was used to sacrifice each mouse.

### Human studies

Demographic information and fresh blood samples were collected from healthy Asian volunteers, including 6 self-reported males and 5 self-reported females aged 20–25 years old (BMI 20.94 ± 3.59 kg/m^2^). All participants provided written informed consent and received no financial compensation. Volunteers were treated with 14 days of creatine monohydrate powder (Myprotein, THG Nutrition Limited, UK). Each volunteer consumed 20 g/day of creatine powder, without changing their lifestyles. Blood samples were collected before breakfast for creatine concentration detection, complete blood count and coagulation tests. After collection, platelets or circulating CD41^+^ cells were immediately isolated for further in vitro studies.

### Infused tumor cell metastasis model and adoptive platelet transfer model

For the metastasis experiment, approximately 1 × 10^5^ mouse tumor cells with 1 × 10^9^ platelets in 100 μL PBS were intravenously injected into each C57BL/6 mouse. After 14 days of tumor cell injection (21 days of MC38 tumor cell injection), mice were sacrificed. Visible metastatic nodules were detected, and metastatic tumor masses were monitored ex vivo under the green fluorescence channel using a VISQUE InVivo Elite system (Vieworks). Lung tissues were immediately collected for further histological examination. For the adoptive platelet transfer, blood samples were collected from donor mice by cardiac puncture, and platelets were immediately isolated for intravenous transfer into the recipient mice.

### Diet and drug treatment

For creatine supplementation, creatine monohydrate (Cat. No. S20197, Yuanye, China) was mixed in water at a dose of 42.5 mg/mL. Each mouse was orally administrated with creatine solution for 400 μL three times per week. Creatine solution was mixed thoroughly before use. For creatine diet and control diet feeding, mice were provided with a 5% w/w creatine monohydrate supplement diet (Cat. No. RC10120, Ready Dietech, China) or a regular control diet (Cat. No. XT01WC-009, Jiangsu Xietong Pharmaceutical Bio-engineering, China) ad libitum. For inhibiting platelet activation, aspirin (Cat. No. MB1790, Meilunbio) at 30 mg/kg in water was given orally once per day to each mouse^18^. For STAT5 inhibition, STAT5-IN-1 (Cat. No. S84762, MedMol, China), a STAT5-specific inhibitor that inhibits both STAT5A and STAT5B, was dissolved in dimethyl sulfoxide (DMSO, Cat. No. PWL064, Meilunbio, China). STAT5-IN-1 was orally administrated to each mouse at a dose of 5 mg/kg on a daily basis. DMSO was used as a control. For in vitro experiment, platelets, isolated MKs, and 293T cells were treated with 0, 0.2, and 2 mM of creatine for up to 48 h. IL-2 (Cat. No. HY-P7037B, MCE) at a dose of 10 ng/mL was applied to Jurkat cells for 24 h for STAT5 activation. For delivering creatine into MKs while bypassing creatine transporter, creatine loaded liposomes were prepared^45^. Briefly, an aqueous phase was prepared by dissolving creatine in PBS to a concentration of 10 mg/mL. Hydrogenated soybean phospholipids (Cat. No. N01003, AVT, China), cholesterol (Cat. No. O01001, AVT, China), and DSPE-PEG 2000 (Cat. No. F01008, AVT, China) were mixed at a mass ratio of 7:3:1 and dissolved in ethanol to form a lipid solution. Creatine@liposomes were made via ethanol injection method. 1 mL of ethanolic solution of lipids was injected into 10 mL of the aqueous phase, allowing spontaneous liposome formation. The mixture was stirred at 50 °C for 1 h to remove residual ethanol, then extruded 5 times through a 200 nm pore-size cellulose acetate membrane filter.

### ShRNA knockdown, siRNA knockdown, and plasmid construction

For stable knockdown of *Slc6a8*, three sets of mouse *Slc6a8*-shRNA (PGMLV-ZsGreen1-Puro) (Cat. No. 103815, 103816, 103818, Genomeditech, China) or Scramble-shRNA (PGMLV-ZsGreen1-Puro) (Cat. No. 2519, Genomeditech, China) were transfected into murine melanoma cells and further selected by 2 μg/mL puromycin dihydrochloride (Cat. No. 60210ES25, YEASEN, China). For siRNA knockdown, three sets of *Gatm* siRNA (Cat. Nos. siB13424174921, siB13424174934, siB13424174947, RIBBIO, China), *Ckb* siRNA (Cat. Nos. siG171113021556, siG171113021601, siG171113021607, RIBBIO, China), or scrambled siRNA (Cat. No. siN0000001-1-5, RIBBIO, China) were transfected into 293T cells or freshly isolated MK cells using liposomal transfection reagent (Cat. No. 40802ES03, YEASEN, China). Knockdown efficiency was confirmed after 24 or 48 h of transfection by qPCR, and the highest efficiency set was selected for subsequent experiments. For plasmid transfection, the constructs were generated from the GFP-containing vector PGMLV-CMV-MCS-EF1-ZsGreen1-T2A-Puro (Cat. No. 10502, Genomeditech, China), inserted with the *Stat5b*-coding fragment and the 3×Flag tag. Plasmids constructs including PGMLV-CMV-3×Flag-EF1-ZsGreen1.T2A-Puro (empty vector with 3×Flag), PGMLV-CMV-Mouse Stat5b (wt)−3×Flag-EF1-ZsGreen1-T2A-Puro, PGMLV-CMV-Mouse Stat5b (p.S127D)−3×Flag-EF1-ZsGreen1-T2A-Puro, PGMLV-CMV-Mouse Stat5b (p.S127A)−3×Flag-EF1-ZsGreen1-T2A-Puro, and PGMLV-CMV-Mouse Stat5b (p.S730A:Y699A:S127A)−3×Flag-EF1-ZsGreen1-T2A-Puro were generated and transfected into the 293 T cells using GMTrans liposomal Transfection Reagent (Cat. No. TG-10015-2, Genomeditech, China) according to the manufacturer’s instruction. After transfection, GFP^+^ cells were collected for further molecular biological experiments. The CKB overexpression plasmid GMLV-CMV-Mouse Ckb-EF1-mScarlet-T2A-Blasticidin and the CKB empty vector plasmid PGMLV-CMV-EF1-mScarlet-T2A-Blasticidin (Cat. No. 21676, Genomeditech, China) were constructed.

### Immunoblot and immunoprecipitation

For immunoblot, an equal amount of protein samples from each group and a standard molecular weight marker (Cat. No. AP13L052, Life-iLab, China) were loaded on a SmartPAGE Precast Protein Gel Plus (Cat. No. SLE015, Smart-Lifesciences, China), and transferred onto a polyvinylidene difluoride (PVDF) membrane (Cat. No. IPVH00010, Millipore), which was subsequently blocked with 5% skimmed milk for 2 h. Membranes were incubated overnight at 4 °C with primary antibodies diluted in Primary Antibody Dilution Buffer (Cat. No. MB9881, Meilunbio, China). After rigorous washing with PBS containing 0.1% Tween-20 (Cat. No. MB2483, Meilunbio, China), membranes were incubated at room temperature for 2 h with a goat anti-rabbit HRP-conjugated IgG antibody (Cat. No. M21001L, Abmart, China; 1:5000) or a goat anti-mouse HRP-conjugated IgG antibody (Cat. No. M21002L, Abmart, China; 1:5000). Target proteins were visualized via a SuperSignal SuperDura Extended Duration Substrate (Cat. No. 36223ES76, YEASEN, China) with a Molecular Imager ChemiDoc XRS System (Bio-Rad). A rabbit anti-GP6 antibody (Cat. No. ab302950, Abcam; 1:1000), a rabbit anti-P2RY12 antibody (Cat. No. 11976-1-AP, Proteintech; 1:1000), a rabbit anti-SLC6A8 antibody (Cat. No. 20299-1-AP, Proteintech; 1:1000), a mouse anti-CKB antibody (Cat. No. 66764-1-Ig, Proteintech; 1:1000), a rabbit anti-GATM antibody (Cat. No. 12801-1-AP, Proteintech; 1:1000), a rabbit anti-STAT5B antibody (Cat. No. 34662, Cell Signaling Technology; 1:1000), a rabbit anti-Phospho-STAT5 antibody (Cat. No. AP0138, ABclonal; 1:1000), a rabbit anti-Histone H3 antibody (Cat. No. 4499, Cell Signaling Technology; 1:1000), and a mouse anti-β-actin antibody (Cat. No. 66009-1-Ig, Proteintech; 1:5000) were used as primary antibodies.

For immunoprecipitation, in brief, approximately 1 × 10^6^ MKs or 293T cells were lysed with IP lysis buffer (Cat. No. PC105, Epizyme, China) supplemented with Protease Inhibitor Cocktail (Cat. No. MB2678, Meilunbio, China) and Phosphatase inhibitor cocktail I (Cat. No. MB12707-1, Meilunbio, China) on ice for 20 min. The lysates were cleared by centrifugation at 14,000 × *g* for 10 min. The supernatants were incubated with an anti-DYKDDDDK (FLAG) Magarose Beads (Cat. No. SM009001, Smart-Lifesciences, China) rotated at 4 °C overnight. The beads were collected using DynaMag-2 magnets (Cat. No. 12321D, Thermo Fisher Scientific) and sequentially washed 3 times with 0.5% Triton X-100 (v/v, Cat. No. 30188928, Sinopharm, China) in PBS. Precipitated proteins were supplemented with 100 μL SDS-PAGE loading buffer (Cat. No. MA0003-D, Meilunbio, China) and incubated at 95 °C for 5 min for immunoblot analysis. For IP-associated immunoblot, a mouse anti-FLAG antibody (Cat. No. F3165, Sigma-Aldrich, 1:4000) and a rabbit anti-Phosphoserine/threonine/tyrosine antibody (Cat. No. 61-8300, Thermo Fisher Scientific; 1:500) were used as primary antibodies. For nuclear fraction extraction from cells, NE-PER Nuclear and Cytoplasmic Extraction Reagents (Cat. No. 78833, Thermo Fisher Scientific) were used. In brief, 1 ×  10^6^ cells were added with Cytoplasmic Extraction Reagent I, and vortexed vigorously for 15 s. After 10 min incubation, Cytoplasmic Extraction Reagent II was added, and the cells were further vortexed for 5 s. The cell lysates were centrifuged at 16,000 × *g* for 5 min. The nuclei-containing pellets were added with nuclear extraction reagent and vortexed for 15 s every 10 min for 3 times. The nuclear lysates were centrifuged at 16,000 × *g* for 10 min. The supernatant was collected for further studies.

### Chromatin immunoprecipitation

For ChIP assay, a ChIP assay kit (Cat. No. p2078, Beyotime, China) was used. Briefly, DNA-bound proteins were fixed using 4% paraformaldehyde (PFA) (Cat. No. MA0192, Meilunbio, China) in approximately 1 × 10^6^ freshly isolated MKs. Chromatin was purified and sonicated by an ultrasound sonicator (Cat. No. Scientz08-IIIC, Ningbo Scientz Biotechnology, China) to generate 500–1000 bp fragments. Supernatant was added with ChIP Dilution Buffer, and Protein A + G agarose (Cat. No. P2078-1, Beyotime, China) was used to precipitate chromatins. A rabbit anti-STAT5B antibody (Cat. No. 34662, Cell Signaling Technology, 1:200) or a rabbit Control IgG antibody (Cat. No. AC005, Abclonal, China; 1:200) was used. Five molar NaCl was mixed with the protein–DNA complexes and incubated at 65 °C for 4 h. The purified DNA fraction was used for qPCR analysis. Primer pairs for ChIP were summarized (Supplementary Table 2). Data were normalized with the nonimmune rabbit IgG and were presented as mean determinants of percentages of input.

### RNA extraction and quantitative real-time PCR

Total RNAs were extracted from adipose tissues, isolated MKs, or cultured cells using an RNAsimple Total RNA kit (Cat. No. DP419, TIANGEN, China). Total RNA from each sample was reversely transcribed using a Hifair II 1st Strand cDNA Synthesis SuperMix (Cat. No. 11123ES60, YEASEN, China). Reverse transcription was performed at 42 °C for 15 min, subsequently 80 °C for 5 min to inactivate the enzyme activity. The cDNA samples were subjected to qPCR using a StepOnePlus Real-Time PCR System (Applied Biosystems) or a Real-Time PCR System (Cat. No. ASA-9600, Baiyuan Gene Technology, China). Each sample was triplicated and in a 10 μL reaction containing Hieff qPCR SYBR Green Master Mix (Cat. No. 11203ES03, YEASEN, China), 50 nM forward and reverse primers, and 2 μL cDNA. The qPCR protocol was executed for 40 cycles, and each cycle consisted of denaturation at 95 °C for 15 s, annealing at 60 °C for 1 min, and extension at 72 °C for 1 min. The primer pairs specific for mouse and human platelet activation-associated genes, etc., were summarized (Supplementary Table 2).

### Histology and immunofluorescence staining

For histological analysis, lung tissues were fixed with 4% PFA (Cat. No. MA0192, Meilunbio, China) for 24 h at room temperature. Paraffin-embedded tissues were cut at a thickness of 5 µm, mounted onto glass slides, baked for 1 h at 60 °C, deparaffinized in Xylene (Cat. No. 10023418, Sinopharm, China), and sequentially rehydrated in 99, 95, and 70% ethanol (Cat. No. 10009218, Sinopharm, China). Tissue slides were counterstained with haematoxylin (Mayer’s) (Cat. No. MB9897, Meilunbio, China) and eosin (Cat. No. MA0164, Meilunbio, China) before dehydration with 95 and 99% ethanol, and were mounted with neutral balsam (Cat. No. 1004160, Sinopharm, China). For immunofluorescence staining, paraffin-embedded lung tissue sections were stained with a chicken anti-GFP antibody (Cat. No. ab13970, Abcam, 1:200) overnight at 4 °C. After rinsing, tissue samples were further stained for 45 min at 37 °C with an Alexa Fluor 488-conjugated goat anti-chicken IgY secondary antibody (Cat. No. ab150169, Abcam, 1:200). Slides were stained with DAPI (Cat. No. C0060, Solarbio, 1:500) for 5 min at room temperature. Positive signals were captured using a fluorescence microscope (Olympus BX53). The captured images were further analyzed using Adobe Photoshop CS software.

### Flow cytometry

For CTC detection, blood samples were collected, transferred to an anticoagulation tube, and centrifuged at 500 × *g* for 5 min. Cells were resuspended in RBC lysis buffer (Cat. No. MA0207, Meilunbio, China) for 5 min to remove RBCs. All samples were kept in 1% PFA for detection of GFP^+^ signal using a Flow Cytometer CytoFLEX S (Beckman Coulter) with a 488 nm laser, 525 nm filter, and analyzed using CytExpert software (Beckman Coulter). For the MK polyploidy test, isolated MKs were stained with Hoechst 33342 (Cat. No. IH0070, Solarbio, China) for 30 min at room temperature. After washing, cells were filtered and examined by FACS canto II (BD Bioscience).

### Molecular docking

The structure of CKB was obtained from the Protein Data Bank (PDB code: 3B6R). The structure of STAT5B was obtained from the AlphaFold protein structure database, with the ordered domains (residues 1–708) used for this study. Molecular dynamics simulations of STAT5B were performed in Amber18 using pmemd.cuda. For MD simulations, energy minimization was firstly carried out using steepest descent algorithm for 3000 steps. Next, protein equilibration was carried out for 1 ns. Then the production simulation was performed in the NVT ensemble at 298 K for 50 ns with a 2.5 fs time step. The representative structure of the most populated cluster obtained from K-means clustering analysis of MD-generated snapshots was used as the initial structure of STAT5B for docking. Protein-protein global docking was then performed for CKB and STAT5B using the Rosetta program v2019.35 with nstruct set to 10,000 to generate 10,000 predicted poses. The interface score (I_sc) from the resulting output file was used as a metric to evaluate CKB-STAT5B binding affinity. The active site residue H66 in CKB was used as a reference residue, and the distances between STAT5B-S127(or STAT5B-Y699) and the reference residue were calculated for all 10000 predicted poses to identify potential bound poses (distance <9 Å).

### In vitro kinase assay

For CKB in vitro kinase assay, wildtype FLAG-STAT5B was purified from 293T cells using anti-FLAG Magarose beads (Cat. No. SM009001, Smart-Lifesciences, China) and DynaMag-2 magnets (Cat. No. 12321D, Thermo Fisher Scientific). Bacterially purified wild-type His-CKB (0.4 μg/μL) (Cat. No. HY-P7897, MCE) was incubated with FLAG-STAT5B (1 μg/μL) in the presence of 0.5 μM ATP or without ATP in a kinase buffer (25 mM Tris-HCl (pH 7.5), 5 mM β-glycerophosphate, 2 mM DTT, 0.1 mM Na_3_VO_4_, 10 mM MgC1_2_) (Cat. No. MKB1235, MesGen, China). After incubation for 1 h at room temperature, FLAG-STAT5B was purified from the kinase buffer and was tested for phosphorylation.

### Phosphoproteomics

Murine MKs were freshly isolated and frozen in liquid nitrogen. Phosphoproteomics was performed with the assistance of Shanghai Biotree Biotech Co., Ltd. In brief, total proteins were extracted from cell samples. After bicinchoninic acid (BCA) quantification, protein samples were added with tris(2-carboxyethyl)phosphine (TCEP) solution for reduction of disulfide bonds, and these disulfide bonds were further alkylated by 15 mM 2-chloroacetamide (CAA) solution for 15 min. Protein samples were digested with trypsin (Cat. No. V5111, Promega) overnight and subjected to peptide desalting. Phosphopeptides were collected using a High-Select Fe-NTA Phosphopeptide Enrichment Kit (Cat. No. A32992, Thermo Fisher Scientific) according to the manufacturer’s instructions.

For each sample, 200 ng of enriched phosphopeptides were separated and analyzed using a nanoElute 2 high-performance nanoflow liquid chromatography system (Bruker) coupled to a timsTOF Pro2 system (Bruker) with a nano-electrospray ion source. Chromatographic separation was performed using a reversed-phase column (PePSep C18, 1.9 μm, 75 μm × 15 cm, Bruker). Mobile phases were H_2_O with 0.1% formic acid (phase A) and acetonitrile with 0.1% formic acid (phase B). Sample separation was performed with a 60 min gradient at a flow rate of 300 nL/min. The phase B ratio and corresponding times were: 2% for 0 min, 5–22% for 45 min, 22–37% for 5 min, 37–80% for 5 min, and 80% for 5 min.

Mass spectrometry analysis was performed using the data-dependent acquisition (DDA) parallel accumulation serial fragmentation (PASEF) mode in positive ion detection, and the scan range was from 100 to 1700 m/z for MS1. During scanning, the impact energy increases linearly with ion mobility, from 20 eV (1/K0 = 0.6 Vs/cm^2^) to 59 eV (1/K0 = 1.6 Vs/cm^2^).

The SpectroMine database search was performed by processing raw MS files using SpectroMine software (4.2.230428.52329) and the built-in pulsar search engine. Database matching parameter settings: database: uniprot_*Mus musculus*_10090_reviewe_2023_09.fasta; fixed modification: carbamidomethyl (C); variable modification: oxidation (M), phosphorylation (S, T, Y), acetyl (N-terminal); proteases: trypsin; max missed cleavages: 2; peptide & PSM false discovery rate (FDR): ≤0.01; peptide tolerance: 20 ppm; other parameters: default. For analysis, the screening criteria for differential peptides were set as follows: fold change ≥1.2 or ≤0.83, with a *P*-value or *P*-value from chi-square test <0.05. Differentially abundant phosphorylated proteins were listed (Supplementary Data 1).

### Colorimetric assay and ELISA assay

After collecting the serum samples and cell lysate samples, circulating creatine and intracellular creatine levels were determined by a Creatine Assay kit (Cat. No. ab65339, Abcam) according to the manufacturer’s protocol using the appropriate standard curve. Absorbance values were detected at 570 nm using a Synergy 2 Multi-Mode Microplate Reader (BioTek). Intracellular phosphocreatine levels were determined by phosphocreatine ELISA kit (Cat. No. USCN-CEV808Ge, Cloud-clone, China) according to the manufacturer’s protocol. Absorbance values were detected at 450 nm using a Synergy 2 Multi-Mode Microplate Reader (BioTek).

### Platelet-tumor cell adhesion assay

EGFP^+^ melanoma cells were grown in 12-well plates to about 80% confluence. Freshly isolated platelets were collected and stained with 5 μM PKH26 (Cat. No. HY-D1451, MCE). Pre-stained platelets at a density of 1 × 10^8^ per well were seeded in each well, followed by gentle centrifugation at 300 × *g* for 5 min. After 30 min incubation, unattached platelets were rinsed out 3 times with PBS. Cells were analyzed under a confocal microscope system (X-LIGHT V2 spinning disk confocal, 89 North; Leica DMi8 microscope, Leica).

### Complete blood count and blood coagulation tests

Blood samples of mice were collected into anti-coagulation tubes and examined by an automated hematology analyzer (Cat. No. BC-2600, Mindary, China). Peripheral blood samples from human volunteers were collected into 2 tubes separately: an EDTA K2 vacuum blood collection tube (Cat. No. 0072497, BD, USA) for examination by an automated hematology analyzer (Cat. No. BC-6800 PLUS, Mindary, China).

### Platelet aggregometry and thromboelastography

Turbidometric platelet aggregation was performed in platelet-rich plasma in response to 10 μM ADP (Cat. No. MB1706, Meilunbio, China), 0.025 U/L thrombin (Cat. No. P/N 386, Chrono-Log, China) or 0.8 μg/mL collagen (Cat. No. P/N 385, Chrono-Log, China). Aggregation was assessed as maximal aggregation at 6 min after agonist challenge. Results were recorded by an aggregometer (Cat. No. Aggram, Helena, UK). For human platelet detection, 250 μL of blood samples were mixed with 25 μL of platelet aggregation reagent (ADP) (Cat. No. 23811000, Sinnowa, China), epinephrine (EPI) (Cat. No. 23802300, Sinnowa, China), arachidonic acid (AA) (Cat. No. 23812010, Sinnowa, China), or platelet aggregation reagent (Collagen) (Cat. No. 23802100, Sinnowa, China). After the mixture, platelet aggregation and adhesion were assessed and analyzed in Aggrestar Platelet Function Analyser (Cat. No. PL-12, Sinnowa, China). For thromboelastography, mouse peripheral blood samples were collected into sodium citrate-anticoagulation tubes (Cat. No. 0065400, BD, USA) and evaluated by a thromboelastography instrument (Cat. No. TEG@5000, Haemonetics Corporation, USA). Reaction time (*R*), K time (*K*), α-angle (angle), and maximum amplitude (MA) were measured.

### Transmission electron microscopy

TEM was performed as our previous published method^19^. In brief, MKs and platelets were fixed by 2.5% glutaraldehyde in Tyrode’s buffer (Cat. No. DF0156, Leagene) for 1.5 h at room temperature and then pelleted by centrifugation at 500 × *g* for 5 min. The pellet was washed and post-fixed with 1% osmium tetroxide in Tyrode’s buffer supplemented with sucrose (25 mg/mL) for 2 h. The samples were dehydrated in ascending ethanol concentrations, acetone, propylene oxide, and embedded. Polymerization was performed under increasing temperatures from 65 °C for 48 h. Ultrathin sections were made by an Ultracut E system (Leica, Germany) and stained with uranyl acetate and lead citrate. The specimens were examined using a Tecnai G2 Spirit electron microscope (FEI, Netherlands).

### Statistics and reproducibility

Statistical analysis was performed using GraphPad Prism (GraphPad, USA). The normality of data was tested by the D’Agostino-Pearson normality test. Statistical differences between the two groups were determined by a two-tailed Student’s *t* test. Differences among multiple groups were evaluated using a one-way or two-way ANOVA test. The data is presented as means ± s.d. Blots in Figs. 2e, g, i, 3g, 4b, g, i, k, 5e–i, and 6d, f have been repeated at least twice. For each panel, samples derived from the same experiment but different gels, with different antibodies processed in parallel. All animals were randomly assigned to groups before experiments. The investigators were not blinded to allocation during experiments and outcome assessment. No statistical method was used to predetermine sample size. No samples, animals or data were excluded.

### Reporting summary

Further information on research design is available in the Nature Portfolio Reporting Summary linked to this article.

## Supplementary information

Supplementary Information

Description of Additional Supplementary Files

Supplementary Data 1

Reporting Summary

Transparent Peer Review File

## Source data

Source data

## Data Availability

The phosphoproteomics data generated in this study have been deposited in the ProteomeXchange Consortium database under accession code PXD065649. Other data generated in this study are provided in the Supplementary Information/Source data file. Source data are provided with this paper.
