# Supplementary material for: Exogenous creatine supplementation promotes tumor metastasis via megakaryocyte creatine kinase B-STAT5B signaling
Source: Nat Commun. 2026 Jul 15;17:8662. doi: 10.1038/s41467-026-74639-z (PMC13490413; doi:10.1038/s41467-026-74639-z)
Supplement: Supplementary file 1 [file 41467_2026_74639_MOESM1_ESM.pdf]

Exogenous creatine supplementation promotes tumor metastasis via megakaryocyte creatine kinase B-STAT5B signaling

Sisi Xie[1#], Ruibo Chen[2#], Xiaoting Sun[3#], Xiaolei Ni[1#], Yuting Wu[1], Linli Cai[1], Yangyi Chen[1], Shuangming Lin[1,4], Mei Rao[1], Liling Chen[1], Yintao Li[5], Yanyong Xu[6], Minfeng Chen[7,8], Dongmei Zhang[7,8], Shun Zhu[2], Ling Yang[2], Wen Liu[2], Ji Zuo[2] and Yunlong Yang[2*]

[1]Department of Cardiology, Basic Scientific Research Center, Longyan First Hospital Affiliated to Fujian Medical University, Longyan, Fujian Province, China

[2]Department of Cellular and Genetic Medicine, School of Basic Medical Sciences, Fudan University, Shanghai, China

[3]School of Pharmaceutical Science, Wenzhou Medical University, Wenzhou, Zhejiang Province, China

[4]Department of Gastroenterology and Anorectal Surgery, Longyan First Hospital, Fujian Medical University, Longyan, Fujian Province, China

[5]Department of Respiratory Oncology, Shandong Cancer Hospital and Institute, Shandong First Medical University and Shandong Academy of Medical Sciences, Jinan, Shandong Province, China

[6]Key Laboratory of Metabolism and Molecular Medicine of the Ministry of Education, Department of Biochemistry and Molecular Biology, School of Basic Medical Sciences, Fudan University Shanghai, Shanghai, China.

[7]State Key Laboratory of Bioactive Molecules and Druggability Assessment, Jinan University, Guangzhou, China

[8]Guangdong Basic Research Center of Excellence for Natural Bioactive Molecules and Discovery of Innovative Drugs, College of Pharmacy, Jinan University, Guangzhou, China

Keywords: megakaryocyte, metastasis, creatine, platelet, STAT5

[#]These authors contributed equally.

*Corresponding author:
Yunlong Yang, Ph.D., Department of Cellular and Genetic Medicine, School of Basic Medical Sciences, Fudan University, Shanghai 200032, P.R. China. Tel: (+86)-21-5423 7311, E-mail: yunlongyang@fudan.edu.cn

Running title: megakaryocytes confer creatine-induced metastasis

Supplementary Figures and Figure Legends

Sup Fig.1

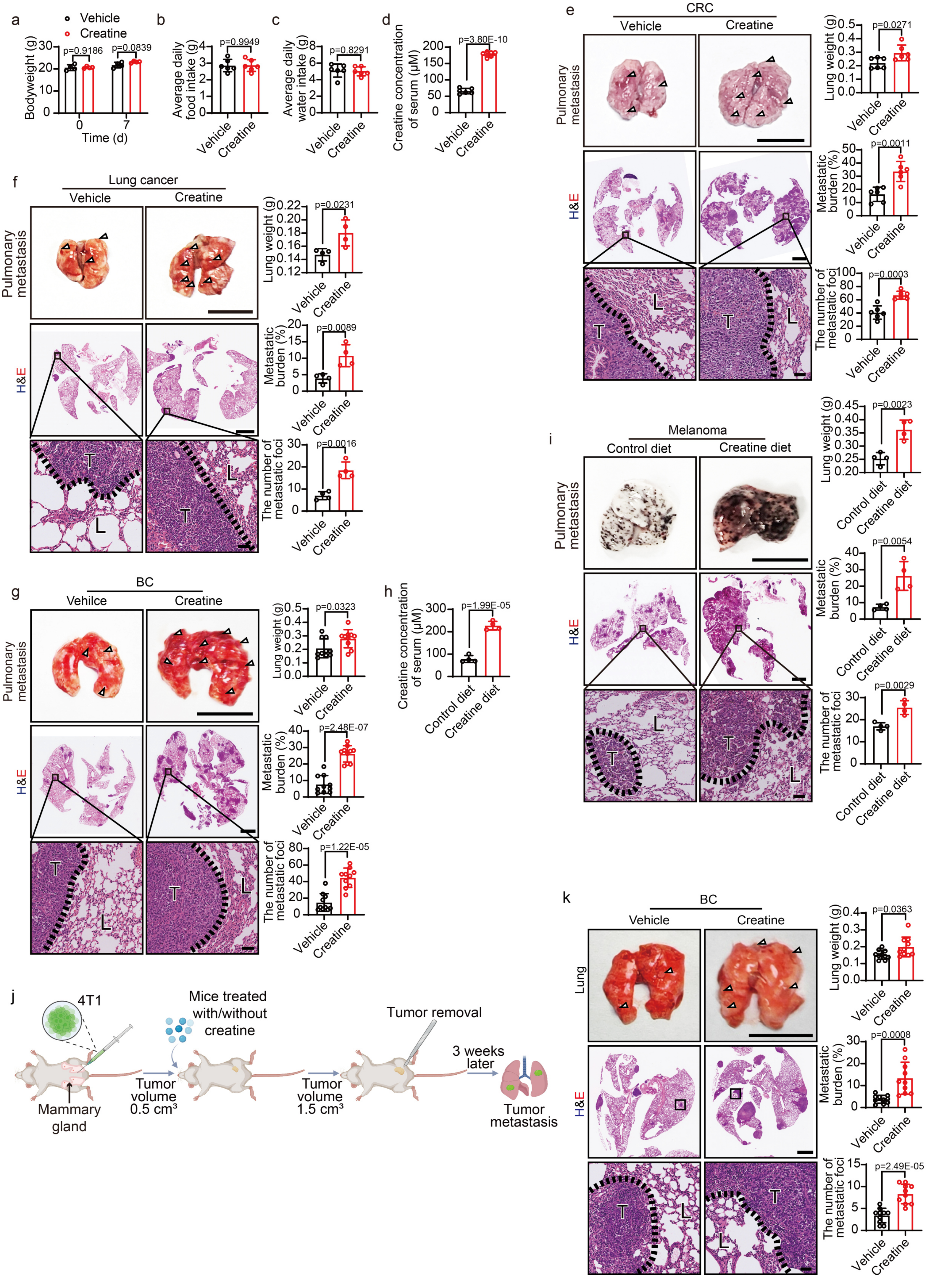

a
Vehicle
Creatine
Bodyweight (g)
p=0.9186
p=0.0839
Time (d)
b
Average daily food intake (g)
p=0.9949
c
Average daily water intake (g)
p=0.8291
d
Creatine concentration of serum (μM)
p=3.80E-10
e
CRC
Vehicle
Creatine
Pulmonary metastasis
H&E
Lung weight (g)
p=0.0271
Metastatic burden (%)
p=0.0011
The number of metastatic foci
p=0.0003
f
Lung cancer
Vehicle
Creatine
Pulmonary metastasis
H&E
Lung weight (g)
p=0.0231
Metastatic burden (%)
p=0.0089
The number of metastatic foci
p=0.0016
g
BC
Vehilce
Creatine
Pulmonary metastasis
H&E
Lung weight (g)
p=0.0323
Metastatic burden (%)
p=2.48E-07
The number of metastatic foci
p=1.22E-05
h
Creatine concentration of serum (μM)
p=1.99E-05
Control diet
Creatine diet
i
Melanoma
Control diet
Creatine diet
Pulmonary metastasis
H&E
Lung weight (g)
p=0.0023
Metastatic burden (%)
p=0.0054
The number of metastatic foci
p=0.0029
j
4T1
Mice treated with/without creatine
Tumor removal
Tumor volume 0.5 cm³
Tumor volume 1.5 cm³
3 weeks later
Mammary gland
Tumor metastasis
k
BC
Vehicle
Creatine
Lung
H&E
Lung weight (g)
p=0.0363
Metastatic burden (%)
p=0.0008
The number of metastatic foci
p=2.49E-05
T
L

Supplementary Figure 1. Exogenous creatine promotes tumor metastasis in various tumor models

a Quantification of bodyweight in vehicle- or creatine-treated mice (*n* = 4 mice per group). **b, c** Healthy mice were treated with vehicle or creatine and the average food intake and water intake were quantified (*n* = 6 tests per group). **d** Circulating creatine concentrations in vehicle- or creatine-treated mice after 7 days of treatment (*n* = 6 mice per group). **e-g** Male C57BL/6 mice were treated with vehicle or creatine for 7 days and were then injected i.v. with MC38 murine CRC cells (**e**) or LLC murine lung cancer cells (**f**). Female BALB/c mice were treated with vehicle or creatine for 7 days and were then injected i.v. with 4T1 murine breast cancer cells (**g**). Representative lung pictures and H&E histological analysis of lung metastasis. Dashed lines mark the borders between tumor (T) and lung (L) tissues. Arrowhead, visible metastatic nodules. Scale bar in upper panels, 1 cm. Scale bar in middle panels, 2 mm. Scale bar in lower panels, 50 μm. Quantifications of lung weight (*n* = 6, 4, and 10 mice per group in **e**, **f**, **g**, respectively). Quantifications of metastatic burden and the number of metastatic foci (*n* = 6, 4, and 10 mice per group in **e**, **f**, **g**, respectively). **h** Circulating creatine concentrations in mice fed with control diet or creatine diet ad libitum for 7 days (*n* = 4 mice per group). **i** Mice were fed with control diet or 5% w/w creatine diet ad libitum and were then injected i.v. with melanoma cells. Representative lung pictures and H&E histological analysis of lung metastasis. Dashed lines mark the borders between tumor (T) and lung (L) tissues. Scale bar in upper panels, 1 cm. Scale bar in middle panels, 2 mm. Scale bar in lower panels, 50 μm. Quantifications of lung weight (*n* = 4 mice per group). Quantifications of metastatic burden and the number of metastatic foci (*n* = 4 mice per group). **j** Schematic diagram of the spontaneous metastasis model of 4T1 breast cancer in BALB/c mice (created in BioRender. Ruibo, C. (2026)

https://BioRender.com/ey8rzg9). **k** Representative lung pictures and H&E histological analysis of lung metastasis. Dashed lines mark the borders between tumor (T) and lung (L) tissues. Arrowhead, visible metastatic nodules. Scale bar in upper panels, 1 cm. Scale bar in middle panels, 2 mm. Scale bar in lower panels, 50 μm. Quantifications of lung weight (*n* = 10 mice per group). Quantifications of metastatic burden and the number of metastatic foci (*n* = 10 mice per group). Statistical significance in **a**, **b**, **c**, **d**, **e**, **f**, **g**, **h**, **i**, and **k** was determined by unpaired two-tailed Student's *t*-test. Source data are provided as a Source Data file. Data presented as mean ± s.d..

Sup Fig.2

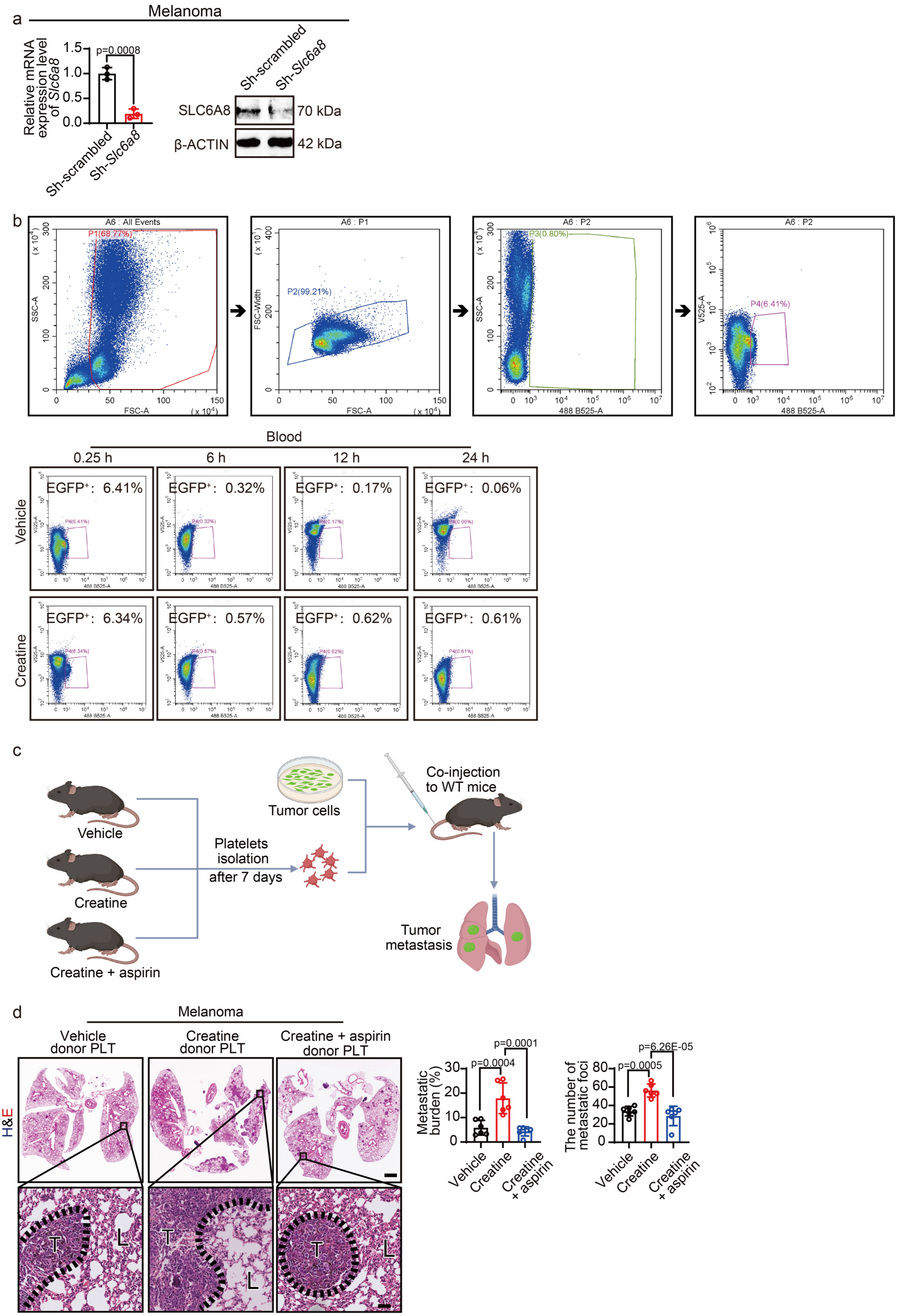
a
Melanoma
Relative mRNA expression level of Slc6a8
p=0.0008
Sh-scrambled
Sh-Slc6a8
SLC6A8
70 kDa
β-ACTIN
42 kDa
b
A6 : All Events
P1(68.77%)
SSC-A
FSC-A
A6 : P1
P2(99.21%)
FSC-Width
A6 : P2
P3(0.80%)
488 B525-A
P4(6.41%)
V525-A
Blood
0.25 h
6 h
12 h
24 h
Vehicle
EGFP+: 6.41%
EGFP+: 0.32%
EGFP+: 0.17%
EGFP+: 0.06%
Creatine
EGFP+: 6.34%
EGFP+: 0.57%
EGFP+: 0.62%
EGFP+: 0.61%
c
Vehicle
Creatine
Creatine + aspirin
Platelets isolation after 7 days
Tumor cells
Co-injection to WT mice
Tumor metastasis
d
Melanoma
Vehicle donor PLT
Creatine donor PLT
Creatine + aspirin donor PLT
H&E
T
L
Metastatic burden (%)
p=0.0004
p=0.0001
The number of metastatic foci
p=0.0005
p=6.26E-05
Vehicle
Creatine
Creatine + aspirin

Supplementary Figure 2. Creatine-promoted metastasis is platelet activation-dependent

a QPCR quantification of *Slc6a8* mRNA expression in melanoma cells transfected with ShRNA of scrambled or *Slc6a8* (n = 3 samples per group). Western blot of SLC6A8 in melanoma cells transfected with ShRNA of scrambled or *Slc6a8*. β-ACTIN marks the loading amount in each lane. Blots have been repeated twice. Samples derive from the same experiment but different gels, with different antibodies processed in parallel. **b** Gating strategies and representative images of flow cytometry of GFP^+ tumor cells in the peripheral blood from vehicle- or creatine-treated mice at various timepoints. **c** Schematic diagram of the adoptive platelet transfer model. Platelets were isolated from vehicle- or creatine-treated donor mice receiving various drug treatments and were co-injected with melanoma cells into healthy recipient mice (created in BioRender. Ruibo, C. (2026) https://BioRender.com/9d97ycz). **d** H&E histological analysis of lung metastasis. Dashed lines mark the borders between tumor (T) and lung (L) tissues. Scale bar in upper panels, 2 mm. Scale bar in lower panels, 50 μm. Quantifications of metastatic burden and the number of metastatic foci (n = 6 mice per group). Statistical significance in **a** was determined by unpaired two-tailed Student's *t*-test. Statistical significance in **d** was determined by one-way ANOVA. Source data are provided as a Source Data file. Data presented as mean ± s.d..

Sup Fig.3

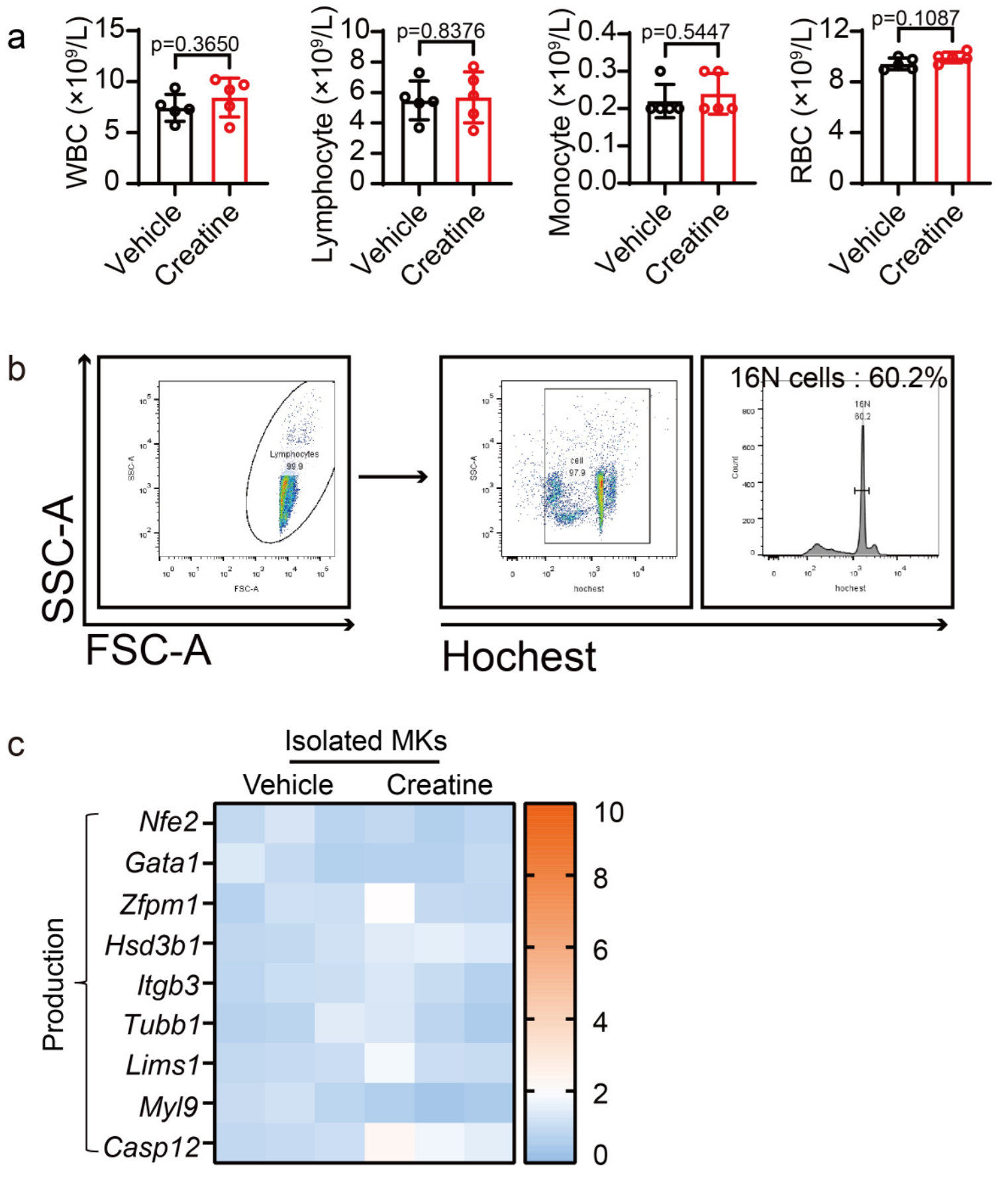
a
WBC (×10⁹/L)
p=0.3650
Lymphocyte (×10⁹/L)
p=0.8376
Monocyte (×10⁹/L)
p=0.5447
RBC (×10⁹/L)
p=0.1087
Vehicle
Creatine
b
SSC-A
FSC-A
Hochest
16N cells : 60.2%
c
Isolated MKs
Vehicle
Creatine
Production
Nfe2
Gata1
Zfpm1
Hsd3b1
Itgb3
Tubb1
Lims1
Myl9
Casp12

Supplementary Figure 3. Creatine insignificantly affects thrombocytopoiesis-associated genes

a WBC, lymphocyte, monocyte, and RBC analysis in whole blood from vehicle- or creatine-treated mice (*n* = 5 mice per group). **b** Gating strategies and Hoechst 33342 staining of isolated MKs. MK polyploidy was validated. **c** Heatmap of thrombocytopoiesis-associated genes of isolated primary MKs from vehicle- or creatine-treated mice (*n* = 3 mice per group). Statistical significance in **a** was determined by unpaired two-tailed Student's *t*-test. Source data are provided as a Source Data file. Data presented as mean ± s.d..

Sup Fig.4

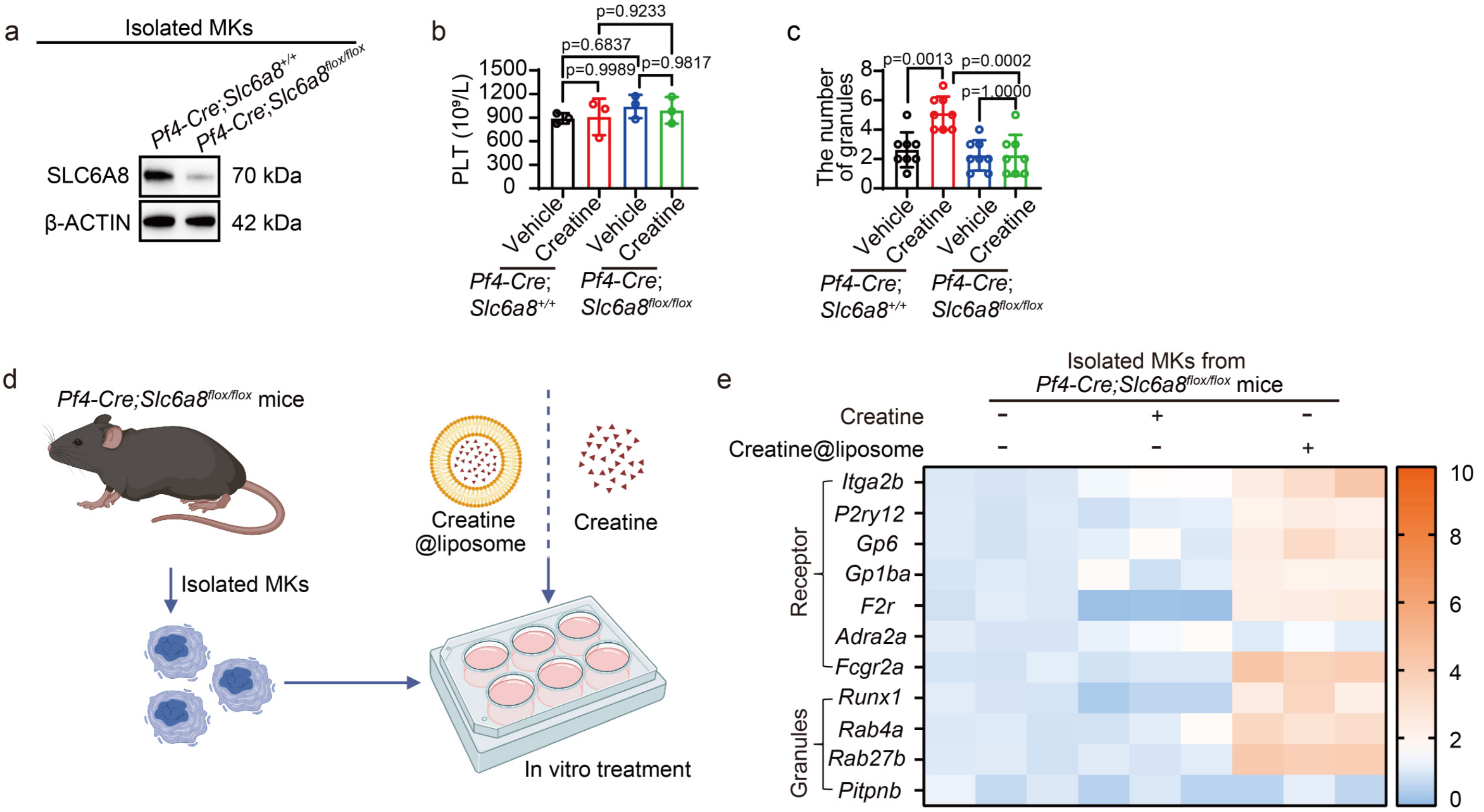

a
Isolated MKs
Pf4-Cre;Slc6a8+/+
Pf4-Cre;Slc6a8flox/flox
SLC6A8
70 kDa
β-ACTIN
42 kDa
b
PLT (10⁹/L)
p=0.9233
p=0.6837
p=0.9989
p=0.9817
Vehicle
Creatine
Vehicle
Creatine
Pf4-Cre; Slc6a8+/+
Pf4-Cre; Slc6a8flox/flox
c
The number of granules
p=0.0013
p=0.0002
p=1.0000
Vehicle
Creatine
Vehicle
Creatine
Pf4-Cre; Slc6a8+/+
Pf4-Cre; Slc6a8flox/flox
d
Pf4-Cre;Slc6a8flox/flox mice
Isolated MKs
Creatine @liposome
Creatine
In vitro treatment
e
Isolated MKs from Pf4-Cre;Slc6a8flox/flox mice
Creatine
Creatine@liposome
Receptor
Itga2b
P2ry12
Gp6
Gp1ba
F2r
Adra2a
Fcgr2a
Granules
Runx1
Rab4a
Rab27b
Pitpnb

Supplementary Figure 4. Genetic depletion of *Slc6a8* in MKs does not affect platelet counts.

a Western blot of SLC6A8 in isolated primary MKs from wt or *Pf4 Cre Slc6a8*$^{flox/flox}$ mice. β-ACTIN marks the loading amount in each lane. Blots have been repeated twice. Samples derive from the same experiment but different gels, with different antibodies processed in parallel. **b** Platelet counts analysis in whole blood from vehicle- or creatine-treated wt or *Pf4 Cre Slc6a8*$^{flox/flox}$ mice (n = 3 mice per group). **c** Quantifications of granules in transmission electron microscopy micrographs of platelets isolated from vehicle- or creatine-treated wt or *Pf4 Cre Slc6a8*$^{flox/flox}$ mice (n = 8 random fields per group). **d** Schematic diagram of the in vitro study in which freshly isolated MKs from *Slc6a8*$^{flox/flox}$ mice were treated with creatine or creatine@liposome (created in BioRender. Ruibo, C. (2026) https://BioRender.com/urkzg4m). **e** Heatmap of platelet receptor- and granule-associated genes of isolated primary MKs treated with creatine or creatine@liposome. (n = 3 samples per group). Statistical significance in **b** and **c** was determined by one-way ANOVA. Source data are provided as a Source Data file. Data presented as mean ± s.d..

Sup Fig.5

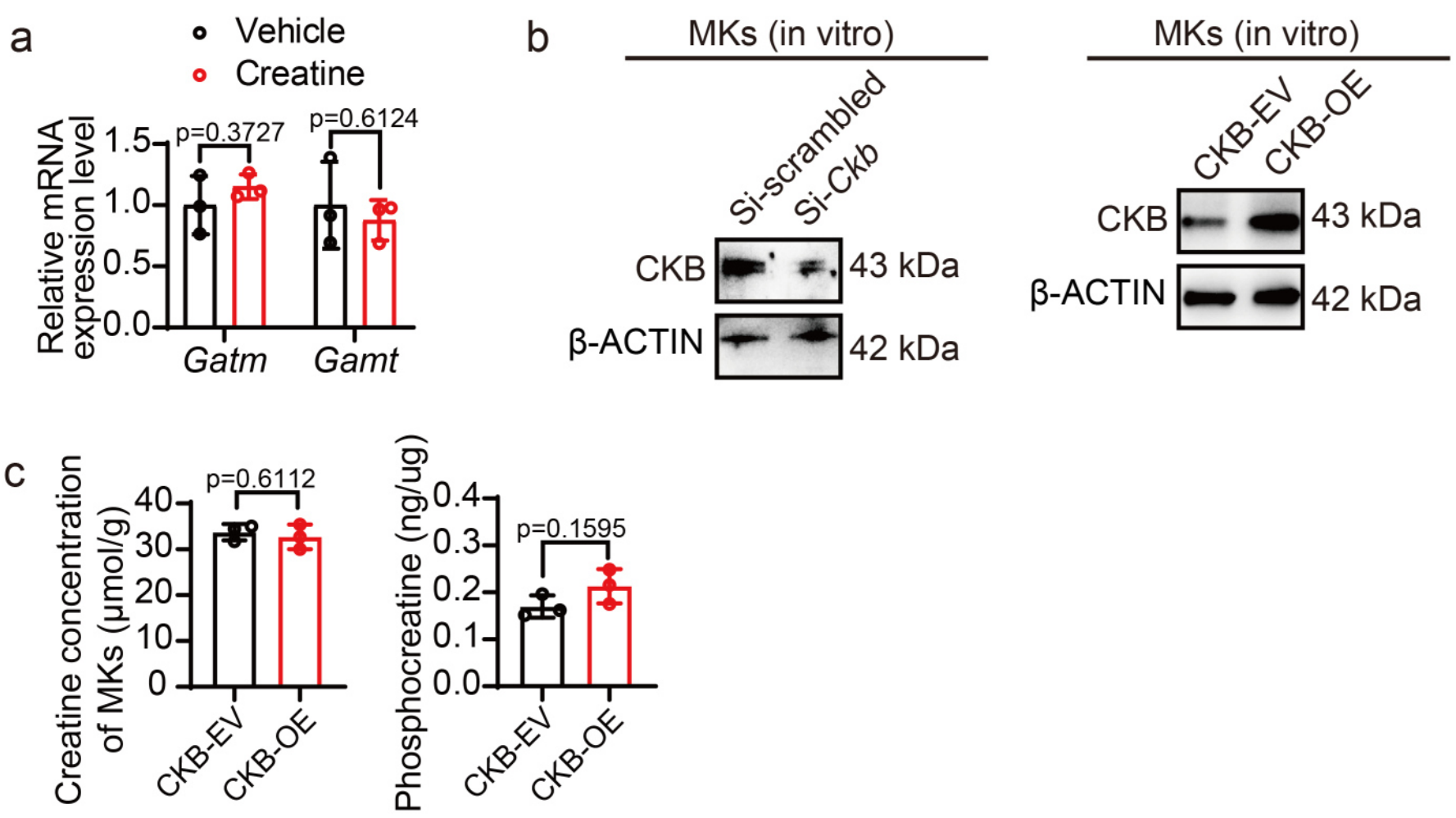
a
Vehicle
Creatine
Relative mRNA expression level
p=0.3727
p=0.6124
Gatm
Gamt
b
MKs (in vitro)
Si-scrambled
Si-Ckb
CKB
β-ACTIN
43 kDa
42 kDa
MKs (in vitro)
CKB-EV
CKB-OE
CKB
β-ACTIN
43 kDa
42 kDa
c
Creatine concentration of MKs (μmol/g)
p=0.6112
CKB-EV
CKB-OE
Phosphocreatine (ng/ug)
p=0.1595
CKB-EV
CKB-OE

Supplementary Figure 5. Exogenous creatine insignificantly affects creatine synthesis enzymes

a QPCR quantification of *Gatm* and *Gamt* mRNA expression in freshly isolated MKs from vehicle- or creatine-treated mice (n = 3 mice per group). **b** Western blot of CKB in si-scrambled- or si-*Ckb* (mouse)-transfected MKs in vitro. Western blot of CKB in empty vector- or *Ckb* overexpression vector-transfected MKs in vitro. β-ACTIN marks the loading amount in each lane. Blots have been repeated twice. Samples derive from the same experiment but different gels, with different antibodies processed in parallel. **c** Intracellular creatine concentrations in empty vector- or *Ckb* overexpression vector-transfected MKs in vitro (n = 3 mice per group). Intracellular phosphocreatine concentrations in empty vector- or *Ckb* overexpression vector-transfected MKs in vitro (n = 3 mice per group). Statistical significance in **a** and **c** was determined by unpaired two-tailed Student's t-test. Source data are provided as a Source Data file. Data presented as mean ± s.d..

Sup Fig.6

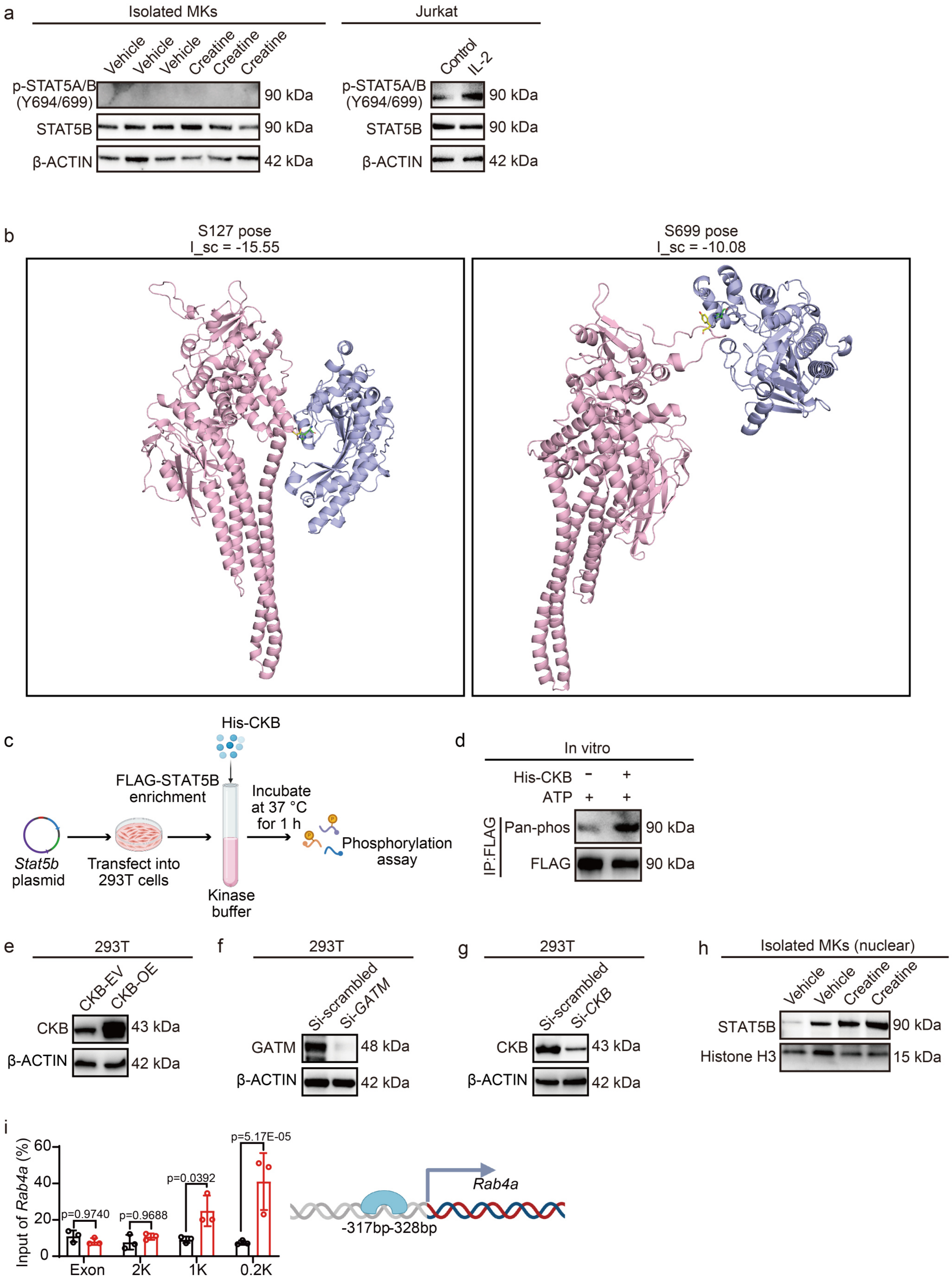
a
Isolated MKs
Jurkat
Vehicle
Vehicle
Vehicle
Creatine
Creatine
Creatine
Control
IL-2
p-STAT5A/B
(Y694/699)
90 kDa
STAT5B
90 kDa
β-ACTIN
42 kDa
b
S127 pose
I_sc = -15.55
S699 pose
I_sc = -10.08
c
His-CKB
FLAG-STAT5B
enrichment
Incubate
at 37 °C
for 1 h
Stat5b
plasmid
Transfect into
293T cells
Kinase
buffer
Phosphorylation
assay
d
In vitro
His-CKB
ATP
IP:FLAG
Pan-phos
FLAG
90 kDa
e
293T
CKB-EV
CKB-OE
CKB
43 kDa
β-ACTIN
42 kDa
f
293T
Si-scrambled
Si-GATM
GATM
48 kDa
β-ACTIN
42 kDa
g
293T
Si-scrambled
Si-CKB
CKB
43 kDa
β-ACTIN
42 kDa
h
Isolated MKs (nuclear)
Vehicle
Vehicle
Creatine
Creatine
STAT5B
90 kDa
Histone H3
15 kDa
i
Input of Rab4a (%)
p=0.9740
p=0.9688
p=0.0392
p=5.17E-05
Exon
2K
1K
0.2K
Rab4a
-317bp-328bp

Supplementary Figure 6. Molecular docking of CKB and STAT5B

a Western blot of phosphorylation of Y699 in STAT5B and total STAT5B in freshly isolated MKs from vehicle- or creatine-treated mice. IL-2-stimulated Jurkat human T cell line served as positive control. β-ACTIN marks the loading amount in each lane. **b** Predicted binding poses of CKB and STAT5B showing the highest binding affinity pose for CKB with STAT5B-S127 site and STAT5B-Y699 site, respectively. CKB and STAT5B are shown in light blue and light pink ribbon, respectively. The S127 and Y699 residues in STAT5B are shown in yellow sticks. The H66 residues in CKB are shown in green sticks. **c** Schematic diagram of the in vitro kinase assay. FLAG-STAT5B and His-CKB were co-incubated in a kinase buffer containing ATP. FLAG-STAT5B was then purified for the phosphorylation test. **d** Western blot of phosphorylation of the STAT5B-FLAG co-incubated with or without His-CKB. FLAG marks the loading amount in each lane. **e** Western blot of CKB in empty vector- or *Ckb* overexpression vector-transfected 293T cells in vitro. β-ACTIN marks the loading amount in each lane. **f** Western blot of GATM in si-scrambled- or si-*Gatm*-transfected 293T cells in vitro. β-ACTIN marks the loading amount in each lane. **g** Western blot of CKB in si-scrambled- or si-*Ckb* (human)-transfected 293T cells in vitro. β-ACTIN marks the loading amount in each lane. **h** Nuclear fractions were separated from freshly isolated MKs from vehicle- or creatine-treated mice. Western blot of STAT5B in total nuclear fraction. Histone H3 marks the loading amount in each lane. **i** ChIP assay of STAT5B binds to different promoter regions of *Rab4a*. Nonimmune IgG was used for input. Coding region (exon) served as controls (n = 3 samples per group). Schematic diagram of the DNA binding model. Blots in **a**, **d**, **e**, **f**, **g**, and **h** have been repeated twice. Samples derive from the same experiment but different gels, with different antibodies processed in parallel. Schematics in **c** and **i** were created in BioRender.

Ruibo, C. (2026) https://BioRender.com/5hsv4us. Statistical significance in **i** was determined by two-way ANOVA. Source data are provided as a Source Data file. Data presented as mean ± s.d..

Sup Fig.7

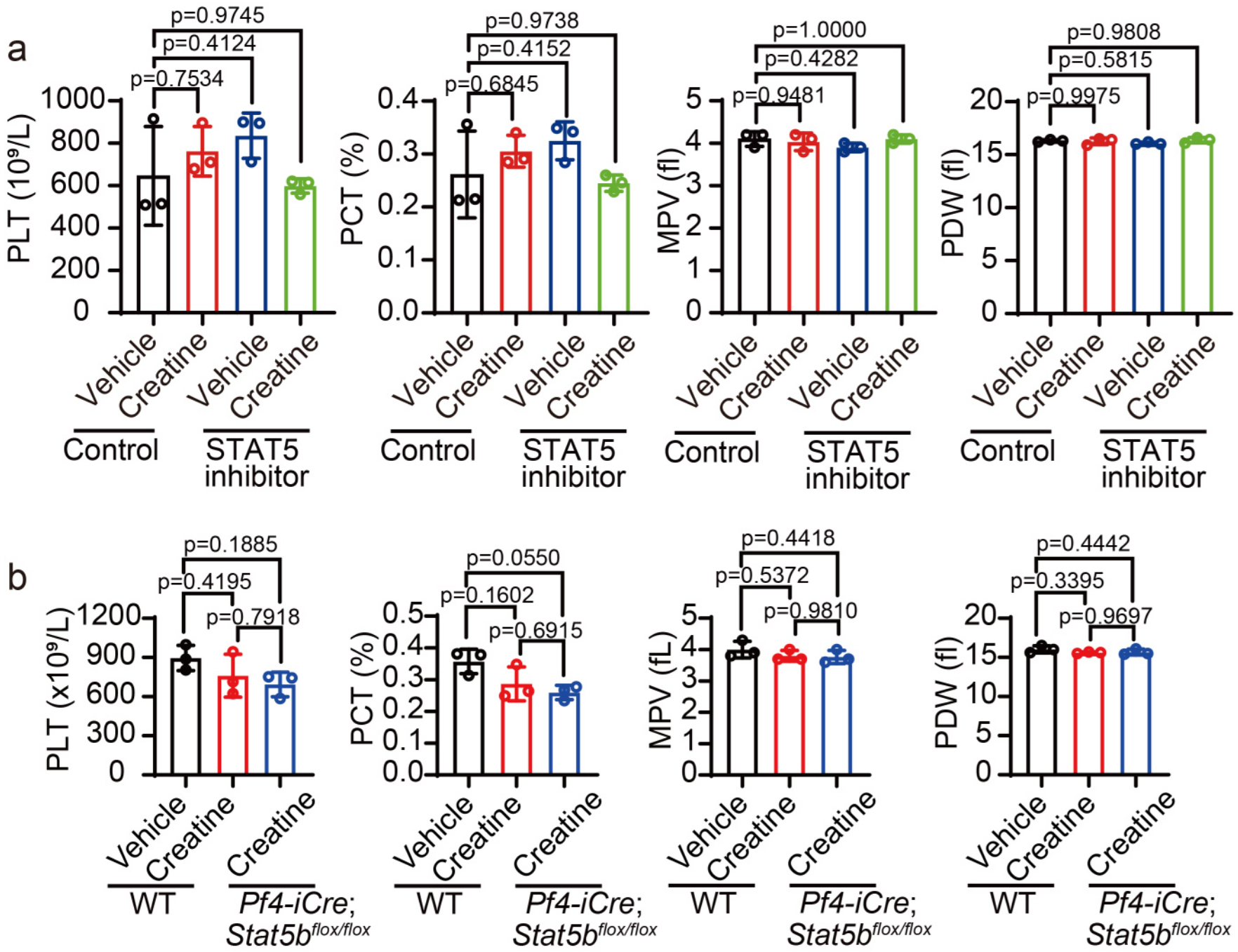
a
PLT (10⁹/L)
1000
800
600
400
200
0
p=0.9745
p=0.4124
p=0.7534
PCT (%)
0.4
0.3
0.2
0.1
0.0
p=0.9738
p=0.4152
p=0.6845
MPV (fl)
5
4
3
2
1
0
p=1.0000
p=0.4282
p=0.9481
PDW (fl)
20
15
10
5
0
p=0.9808
p=0.5815
p=0.9975
Vehicle
Creatine
Vehicle
Creatine
Control
STAT5 inhibitor
b
PLT (x10⁹/L)
1200
900
600
300
0
p=0.1885
p=0.4195
p=0.7918
PCT (%)
0.5
0.4
0.3
0.2
0.1
0.0
p=0.0550
p=0.1602
p=0.6915
MPV (fL)
5
4
3
2
1
0
p=0.4418
p=0.5372
p=0.9810
PDW (fl)
20
15
10
5
0
p=0.4442
p=0.3395
p=0.9697
Vehicle
Creatine
Creatine
WT
Pf4-iCre;
Stat5b flox/flox

Supplementary Figure 7. Pharmacological inhibition of STAT5 or MK-specific *Stat5b* depletion insignificantly affects platelet count

a Platelet counts (PLT), plateletcrit (PCT), platelet distribution width (PDW), mean platelet volume (MPV) analysis in whole blood from vehicle- or creatine-treated mice with or without STAT5-specific inhibitor administration (*n* = 3 samples per group). **b** Platelet counts (PLT), plateletcrit (PCT), platelet distribution width (PDW), mean platelet volume (MPV) analysis in whole blood from vehicle- or creatine-treated wt or Pf4 iCre Stat5b$^{flox/flox}$ mice (*n* = 3 samples per group). Statistical significance in **a** and **b** was determined by one-way ANOVA. Source data are provided as a Source Data file. Data presented as mean ± s.d..

Sup Fig.8

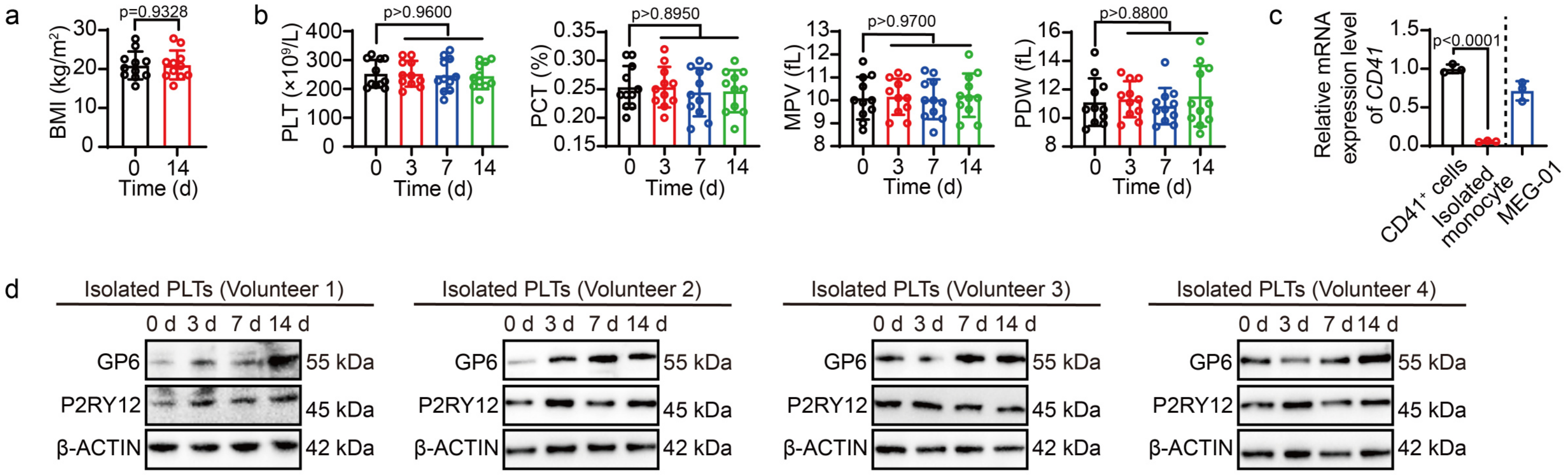
a
p=0.9328
BMI (kg/m²)
30
20
10
0
0
14
Time (d)
b
p>0.9600
PLT (×10⁹/L)
400
300
200
100
0
0
3
7
14
Time (d)
p>0.8950
PCT (%)
0.35
0.30
0.25
0.20
0.15
Time (d)
p>0.9700
MPV (fL)
13
12
11
10
9
8
Time (d)
p>0.8800
PDW (fL)
16
14
12
10
8
Time (d)
c
Relative mRNA expression level of CD41
1.5
1.0
0.5
0.0
p<0.0001
CD41⁺ cells
Isolated monocyte
MEG-01
d
Isolated PLTs (Volunteer 1)
Isolated PLTs (Volunteer 2)
Isolated PLTs (Volunteer 3)
Isolated PLTs (Volunteer 4)
0 d
3 d
7 d
14 d
GP6
P2RY12
β-ACTIN
55 kDa
45 kDa
42 kDa

Supplementary Figure 8. Exogenous creatine supplementation increases hyperactive platelets in humans

a Quantifications of BMI of volunteers before and after 14 days of creatine treatment (*n* = 11 volunteers per group). **b** Platelet counts (PLT), plateletcrit (PCT), platelet distribution width (PDW), mean platelet volume (MPV) analysis in whole blood from volunteers at various timepoints (*n* = 11 volunteers per group). **c** QPCR quantification of *CD41* mRNA expression in freshly isolated $CD41^{+}$ cells, isolated monocytes, and MEG-01 cells (*n* = 3 samples per group). **d** For functional study, 4 out of 11 volunteers were randomly selected and platelets were isolated from peripheral blood. Western blot of GP6 and P2RY12 in isolated platelets from each volunteer at various timepoints. β-ACTIN marks the loading amount in each lane. Blots have been repeated twice. Samples derive from the same experiment but different gels, with different antibodies processed in parallel. Statistical significance in **a** and **c** was determined by unpaired two-tailed Student's *t*-test. Statistical significance in **b** was determined by one-way ANOVA. Source data are provided as a Source Data file. Data presented as mean ± s.d..

Supplementary Tables

Supplementary Table 1. Baseline characteristics of 11 healthy volunteers

**Gender**	Female	6
	Male	5
**Age**		20.91 ± 1.45
**Height (cm)**		165.09 ± 7.08
**Weight (kg)**		57.64 ± 14.16
**BMI (kg/m^2)**		20.94 ± 3.59
**NSAID**	Yes	0
	No	11

Supplementary Table 2. Primers for qPCR and ChIP used in the experiment

**Primer Name**	**Primers (5'-3')**
**Primers for qPCR**	
m-*Rab4a*-F	CTCCAGGTTCGCACAAGAGAA
m-*Rab4a*-R	TCCTTGCACACTGCATGAAAG
m-*Plcg1*-F	ATCCAGCAGTCCTAGAGCCTG
m-*Plcg1*-R	GGATGGCGATCTGACAAGC
m-*Gp1ba*-F	CATTCCCAGCACACTTGTAGT
m-*Gp1ba*-R	TGAGGTGAGTGAAATGCACCA
m-*Orai1*-F	GATCGGCCAGAGTTACTCCG
m-*Orai1*-R	TGGGTAGTCATGGTCTGTGTC
m-*Itga2b*-F	TTCTTGGGTCCTAGTGCTGTT
m-*Itga2b*-R	CGCTTCCATGTTTGTCCTTATGA
m-*P2ry12*-F	ATGGATATGCCTGGTGTCAACA
m-*P2ry12*-R	AGCAATGGGAAGAGAACCTGG
m-*F2r*-F	TGAACCCCCGCTCATTCTTTC
m-*F2r*-R	CCAGCAGGACGCTTTCATTTTT
m-*Adra2a*-F	GTGACACTGACGCTGGTTTG
m-*Adra2a*-R	CCAGTAACCCATAACCTCGTTG
m-*Gp6*-F	CTTTCTTCTGTATTGGGCTGTGT
m-*Gp6*-R	CGTCCAGCATTACTTCTTTCCA
m-*Fcgr2a*-F	AGGGCCTCCATCTGGACTG
m-*Fcgr2a*-R	GTGGTTCTGGTAATCATGCTCTG
m-*Actb*-F	GGCTGTATTCCCCTCCATCG
m-*Actb*-R	CCAGTTGGTAACAATGCCATGT
m-*Gatm*-F	GCTTCCTCCCGAAATTCCTGT
m-*Gatm*-R	CCTCTAAAGGGTCCCATTCGT
m-*Gamt*-F	CACGCACCTGCAAATCCTG
m-*Gamt*-R	TACCGAAGCCCACTTCCAAGA
m-*Ckb*-F	AGTTCCCTGATCTGAGCAGC
m-*Ckb*-R	GAATGGCGTCGTCCAAAGTAA
m-*Runx1*-F	GATGGCACTCTGGTCACCG
m-*Runx1*-R	GCCGCTCGGAAAAGGACAA
m-*Rab27b*-F	CGTCAGGAAAAGCGTTTAAGGT
m-*Rab27b*-R	AGAAGCTCTGTTGACTGGTGA
m-*Pitpnb*-F	TCCATTCAGTCAAGACCAAGAGA
m-*Pitpnb*-R	AACCCTTCTTACGCATTGTTTCT
m-*Nfe2*-F	TCCTCAGCAGAACAGGAACAG
m-*Nfe2*-R	GGCTCAAAAGATGTCTCACTTGG
m-*Gata1*-F	TGGGGACCTCAGAACCCTTG
m-*Gata1*-R	GGCTGCATTTGGGGAAGTG
m-*Zfpm1*-F	AGGAAACAGAGCAATCCCCG

m-*Zfpm1*-R	CAGGTGGGCTCACATCTTCT
m-*Hsd3b1*-F	TGGACAAAGTATTCCGACCAGA
m-*Hsd3b1*-R	GGCACACTTGCTTGAACACAG
m-*Itgb3*-F	CCACACGAGGCGTGAACTC
m-*Itgb3*-R	CTTCAGGTTACATCGGGGTGA
m-*Tubb1*-F	CTGGGAGGTGATCGGGGAA
m-*Tubb1*-R	GCACATACTTCTTACCGTAGGCT
m-*Lims1*-F	CAGCGTCCTTGAAGCTAATGG
m-*Lims1*-R	TTACTGTTCACGATCTTCTCAGC
m-*My19*-F	ACAGCGCCGAGGACTTTTC
m-*My19*-R	AGACATTGGACGTAGCCCTCT
m-*Casp12*-F	AGACAGAGTTAATGCAGTTTGCT
m-*Casp12*-R	TTCACCCCACAGATTCCTTCC
h-*RAB4A*-F	GTCCGTGACGAGAAGTTATTACC
h-*RAB4A*-R	TGAGCGCACTTGTTTCCAAAA
h-*PLCG1*-F	GGAAGACCTCACGGGACTTTG
h-*PLCG1*-R	GCGTTTTCAGGCGAAATTCCA
h-*ORAI1*-F	GACTGGATCGGCCAGAGTTAC
h-*ORAI1*-R	GTCCGGCTGGAGGCTTTAAG
h-*ITGA2B*-F	GATGAGACCCGAAATGTAGGC
h-*ITGA2B*-R	GTCTTTTCTAGGACGTTCCAGTG
h-*P2RY12*-F	CACTGCTCTACACTGTCCTGT
h-*P2RY12*-R	AGTGGTCCTGTTCCCAGTTTG
h-*GP6*-F	TCCCGGCCATGAAGAGAAGT
h-*GP6*-R	TTACGTCCCCTCCTGACGAC
h-*GP1BA*-F	CTGTGAGGTCTCCAAAGTGGC
h-*GP1BA*-R	GTGAGGCGAGTGTAAGGCATC
h-*F2R*-F	CCACCTTAGATCCCCGGTCAT
h-*F2R*-R	GTGGGAGGCTGACTACAAACA
h-*ADRA2A*-F	TCGTCATCATCGCCGTGTTC
h-*ADRA2A*-R	AAGCCTTGCCGAAGTACCAG
h-*FCGR2A*-F	TTTGAGATGAGTAATCCCAGCCA
h-*FCGR2A*-R	TCAGGCCCAGTCTCCATTTTA
h-*ACTB*-F	CATGTACGTTGCTATCCAGGC
h-*ACTB*-R	CTCCTTAATGTCACGCACGAT
h-*RUNX1*-F	CTGCCCATCGCTTTCAAGGT
h-*RUNX1*-R	GCCGAGTAGTTTTCATCATTGCC
h-*RAB27B*-F	TAGACTTTCGGGAAAAACGTGTG
h-*RAB27B*-R	AGAAGCTCTGTTGACTGGTGA
h-*PITPNB*-F	CTGTTCAGGAGTATCAGGTTGGG
h-*PITPNB*-R	GGCACTTTGCTCTTTAGGTGA

**Primers for ChIP**	
m-*Rab4a*-promoter-200-F	CTCAGGTGGGTCAAACTCCC
m-*Rab4a*-promoter-200-R	GGTCACCAGAACTCTCCTGC
m-*Rab4a*-promoter-1000-F	ACCTCTGCACTTGGCAGTTT
m-*Rab4a*-promoter-1000-R	ATCCAGTACACCGCAGGAAC
m-*Rab4a*-promoter-2000-F	ACCTCTGCACTTGGCAGTTT
m-*Rab4a*-promoter-2000-R	TGGCCTCTGATCCTATCCAGT
m-*Rab4a*-exon-F	CATCACCAGCCGAGAAACCT
m-*Rab4a*-exon-R	GTCCTTCTTGTTCCCGCAGA
m-*P2ry12*-promoter-200-F	AGGAGGACAGGAGAGGCAAT
m-*P2ry12*-promoter-200-R	TTCCTGTCAGAGGACCTGGA
m-*P2ry12*-promoter-1000-F	AACCGCTTGACTCTGGGAAG
m-*P2ry12*-promoter-1000-R	CATGTGATCTGGGGTGGTGG
m-*P2ry12*-promoter-2000-F	TACACCAGCTGTCGAGCAAG
m-*P2ry12*-promoter-2000-R	GGTAGACATGGATCCGGCTG
m-*P2ry12*-exon-F	TTGCACGGATTCCCTACACC
m-*P2ry12*-exon-R	GAAGCAGCCCCTTGGGTAAT
m-*Gp6*-promoter-200-F	ATGTGCCAATGGAGAGGCAA
m-*Gp6*-promoter-200-R	CTGCCAGGTCCAAAGGTCAT
m-*Gp6*-promoter-1000-F	GTCAGGAATGAGGTATGTTTTTCAC
m-*Gp6*-promoter-1000-R	TGCTATTTCTCCTGCTCTAGCC
m-*Gp6*-promoter-2000-F	ACGTGGGTGTTAATCCAGCC
m-*Gp6*-promoter-2000-R	CACGGCTTTGTGGTCAAGTG
m-*Gp6*-exon-F	ATGCCTTGGTGCCACGATTA
m-*Gp6*-exon-R	TCCCTCTGCTGAAAGCCAAG
