# Supplementary material for: Exogenous creatine supplementation promotes tumor metastasis via megakaryocyte creatine kinase B-STAT5B signaling
Source: Nat Commun. 2026 Jul 15;17:8662. doi: 10.1038/s41467-026-74639-z (PMC13490413; doi:10.1038/s41467-026-74639-z)
Supplement: Supplementary file 2 [file 41467_2026_74639_MOESM2_ESM.pdf]

Description of Additional Supplementary Files:

Supplementary Data 1: Differentially phosphorylated peptides in freshly isolated MKs from vehicle- or creatine-treated mice
