# Supplementary material for: Exogenous creatine supplementation promotes tumor metastasis via megakaryocyte creatine kinase B-STAT5B signaling
Source: Nat Commun. 2026 Jul 15;17:8662. doi: 10.1038/s41467-026-74639-z (PMC13490413; doi:10.1038/s41467-026-74639-z)
Supplement: Supplementary file 3 [file 41467_2026_74639_MOESM4_ESM.pdf]

nature portfolio

Corresponding author(s): Yunlong Yang

Last updated by author(s): Apr 22, 2026

Reporting Summary

Nature Portfolio wishes to improve the reproducibility of the work that we publish. This form provides structure for consistency and transparency in reporting. For further information on Nature Portfolio policies, see our Editorial Policies and the Editorial Policy Checklist.

Statistics

For all statistical analyses, confirm that the following items are present in the figure legend, table legend, main text, or Methods section.

n/a	Confirmed	
☐	☒	The exact sample size (*n*) for each experimental group/condition, given as a discrete number and unit of measurement
☐	☒	A statement on whether measurements were taken from distinct samples or whether the same sample was measured repeatedly
☐	☒	The statistical test(s) used AND whether they are one- or two-sided<br>*Only common tests should be described solely by name; describe more complex techniques in the Methods section.*
☐	☒	A description of all covariates tested
☐	☒	A description of any assumptions or corrections, such as tests of normality and adjustment for multiple comparisons
☐	☒	A full description of the statistical parameters including central tendency (e.g. means) or other basic estimates (e.g. regression coefficient) AND variation (e.g. standard deviation) or associated estimates of uncertainty (e.g. confidence intervals)
☐	☒	For null hypothesis testing, the test statistic (e.g. *F*, *t*, *r*) with confidence intervals, effect sizes, degrees of freedom and *P* value noted<br>*Give P values as exact values whenever suitable.*
☒	☐	For Bayesian analysis, information on the choice of priors and Markov chain Monte Carlo settings
☒	☐	For hierarchical and complex designs, identification of the appropriate level for tests and full reporting of outcomes
☒	☐	Estimates of effect sizes (e.g. Cohen's *d*, Pearson's *r*), indicating how they were calculated

Our web collection on statistics for biologists contains articles on many of the points above.

Software and code

Policy information about availability of computer code

Data collection

- Visible metastatic nodules were detected and metastatic tumor masses were monitored ex vivo using a VISQUE InVivo Elite system (Vieworks).
- Immunofluorescence images were captured using a fluorescence microscope (Olympus BX53). The captured images were further analyzed using the Adobe Photoshop CS software and Image J.
- The confocal images were recorded under a confocal microscope system (X-LIGHT V2 spinning disk confocal, 89 North; Leica DMi8 microscope, Leica) and quantified using Adobe Photoshop software CS5 extended (Adobe) and Image J.
- For H&E staining, images were captured using a light microscope (Olympus CX31). The captured images were further analyzed using the Adobe Photoshop CS software and Image J.
- qPCR were performed using a StepOnePlus Real-Time PCR System (Applied Biosystems).
- Flow cytometry files of CTC detection were collected using a Flow Cytometer CytoFLEX S (Beckman Coulter) with a 488 nm laser, 525 nm filter, and analyzed using CytExpert software (Beckman Coulter). Flow cytometry files of MK polyploidy test were collected by FACS canto II (BD Bioscience).
- Protein-protein global docking was performed using Rosetta program v2019.35.
- The SpectroMine database search was performed by processing raw MS files using SpectroMine software (4.2.230428.52329) and the built-in Pulsar search engine.
- The platelet aggregation and adhesion were assessed in Aggrestar Platelet Function Analyser (Cat. No. PL-12, Sinnowa, China). The thromboelastography were evaluated by a thromboelastography instrument (Cat. No. TEG@5000, Haemonetics Corporation, USA).
- Transmission electron microscopy (TEM) were performed using a Tecnai G2 Spirit electron microscope (FEI, Netherlands).

Data analysis	Microsoft 365 Excel; GraphPad Prism 9.3.0; CleVue software (Vieworks)

For manuscripts utilizing custom algorithms or software that are central to the research but not yet described in published literature, software must be made available to editors and reviewers. We strongly encourage code deposition in a community repository (e.g. GitHub). See the Nature Portfolio guidelines for submitting code & software for further information.

Data

Policy information about availability of data

All manuscripts must include a data availability statement. This statement should provide the following information, where applicable:

- Accession codes, unique identifiers, or web links for publicly available datasets
- A description of any restrictions on data availability
- For clinical datasets or third party data, please ensure that the statement adheres to our policy

The phosphoproteomics data generated in this study have been deposited in the ProteomeXchange Consortium database under accession code PXD065649 [https://proteomecentral.proteomexchange.org/ui?pxid=PXD065649]. Other data generated in this study are provided in the Supplementary Information/Source Data file. Source data are provided with this paper.

Research involving human participants, their data, or biological material

Policy information about studies with human participants or human data. See also policy information about sex, gender (identity/presentation), and sexual orientation and race, ethnicity and racism.

Reporting on sex and gender	This study included 6 self-reported males and 5 self-reported females
Reporting on race, ethnicity, or other socially relevant groupings	The participants are healthy Asian volunteers.
Population characteristics	The participants are healthy Asian volunteers at the age between 20-25-year-old (BMI 15.6-27.8 kg/m2) with written informed consent.
Recruitment	The following were the inclusion criteria: healthy volunteers between ages 18 and 35 years; all subjects had normal heart function, no chronic inflammatory conditions, and were not taking any medications which would interfere with the study; all subjects signed informed consent and were willing to take creatine and no caffeine during the trial; all subjects had baseline bloodwork, which included heart testing, and all were normal.
Ethics oversight	All human studies were approved by the Ethical Review Committee in Longyan First Hospital Affiliated to Fujian Medical University (LYREC2023-019-01)

Note that full information on the approval of the study protocol must also be provided in the manuscript.

Field-specific reporting

Please select the one below that is the best fit for your research. If you are not sure, read the appropriate sections before making your selection.

- [x] Life sciences
- [ ] Behavioural & social sciences
- [ ] Ecological, evolutionary & environmental sciences

For a reference copy of the document with all sections, see nature.com/documents/nr-reporting-summary-flat.pdf

Life sciences study design

All studies must disclose on these points even when the disclosure is negative.

Sample size	We did not perform any statistical calculations to predetermine the sample size for the in vivo experiments. Instead, the sample size was determined based on our extensive prior in vivo studies, which confirmed the extent and consistency of measurable differences. This approach ensures both statistical and biological significance. We utilized an optimal number of animals to comply with the 3Rs principle regarding animal ethical approval.
Data exclusions	No data were excluded.
Replication	Murine studies: Experiments were conducted at least twice. The fundamental tumor experiments were carried out at least twice across multiple institutions and were reproducible. Phosphoproteomics studies: Quality sample control was performed before subjecting those analyses. The phosphoproteomics were performed once using three independent biological replicants because of further validation.
Randomization	Murine studies: Age, gender, and species background-matched mice were randomly allocated into the groups for all animal experiments.
Blinding	Murine studies: Blinding was not performed for experiments involving different tumor metastatic model. Phosphoproteomics: the staff who were blinded to the experimental groups did mass spectrometry analysis.

Reporting for specific materials, systems and methods

We require information from authors about some types of materials, experimental systems and methods used in many studies. Here, indicate whether each material, system or method listed is relevant to your study. If you are not sure if a list item applies to your research, read the appropriate section before selecting a response.

Materials & experimental systems

n/a	Involved in the study	
☐	☒	Antibodies
☐	☒	Eukaryotic cell lines
☒	☐	Palaeontology and archaeology
☐	☒	Animals and other organisms
☒	☐	Clinical data
☒	☐	Dual use research of concern
☒	☐	Plants

Methods

n/a	Involved in the study	
☒	☐	ChIP-seq
☐	☒	Flow cytometry
☒	☐	MRI-based neuroimaging

Antibodies

Antibodies used

Information for antibodies for this study are,
-a rabbit anti-human CD41/ITGA2B antibody (Cat. No. A11836, Abclonal, China)
-an Alexa Fluor 647-conjugated donkey anti-rabbit antibody (Cat. No. A31573, Invitrogen)
-a goat anti-rabbit HRP-conjugated IgG antibody (Cat. No. M21001L, Abmart. Shanghai, China)
-a goat anti-mouse HRP-conjugated IgG antibody (Cat. No. M21002L, Abmart. Shanghai, China)
-a rabbit anti-GP6 antibody (Cat. No. ab302950, Abcam)
-a rabbit anti-P2RY12 antibody (Cat. No. 11976-1-AP, Proteintech)
-a rabbit anti-SLC6A8 antibody (Cat. No. 20299-1-AP, Proteintech)
-a mouse anti-CKB antibody (Cat. No. 66764-1-Ig, Proteintech)
-a rabbit anti-GATM antibody (Cat. No. 12801-1-AP, Proteintech)
-a rabbit anti-STAT5B antibody (Cat. No. 34662, Cell Signaling Technology)
-a rabbit anti-Phospho-STAT5 antibody (Cat. No. AP0138, ABclonal)
-a rabbit anti-Histone H3 antibody (Cat. No. 4499, Cell Signaling Technology)
-a mouse anti-β-actin antibody (Cat. No. 66009-1-Ig, Proteintech)
-a mouse anti-FLAG antibody (Cat. No. F3165, Sigma-Aldrich)
-a rabbit anti-Phosphoserine/threonine/tyrosine antibody (Cat. No. 61-8300, Thermo Fisher Scientific)
-a rabbit Control IgG antibody (Cat. No. AC005, Abclonal, China)
-a chicken anti-GFP antibody (Cat. No. ab13970, Abcam)
-an Alexa Fluor 488-conjugated goat anti-chicken IgY secondary antibody (Cat. No. ab150169, Abcam)
Information for antibodies for this study are,
-a rabbit anti-human CD41/ITGA2B antibody (Cat. No. A11836, Abclonal, China)
-an Alexa Fluor 647-conjugated donkey anti-rabbit antibody (Cat. No. A31573, Invitrogen)
-a goat anti-rabbit HRP-conjugated IgG antibody (Cat. No. M21001L, Abmart. Shanghai, China)
-a goat anti-mouse HRP-conjugated IgG antibody (Cat. No. M21002L, Abmart. Shanghai, China)
-a rabbit anti-GP6 antibody (Cat. No. ab302950, Abcam)
-a rabbit anti-P2RY12 antibody (Cat. No. 11976-1-AP, Proteintech)
-a rabbit anti-SLC6A8 antibody (Cat. No. 20299-1-AP, Proteintech)
-a mouse anti-CKB antibody (Cat. No. 66764-1-Ig, Proteintech)
-a rabbit anti-GATM antibody (Cat. No. 12801-1-AP, Proteintech)
-a rabbit anti-STAT5B antibody (Cat. No. 34662, Cell Signaling Technology)
-a rabbit anti-Phospho-STAT5 antibody (Cat. No. AP0138, ABclonal)
-a rabbit anti-Histone H3 antibody (Cat. No. 4499, Cell Signaling Technology)
-a mouse anti-β-actin antibody (Cat. No. 66009-1-Ig, Proteintech)
-a mouse anti-FLAG antibody (Cat. No. F3165, Sigma-Aldrich)
-a rabbit anti-Phosphoserine/threonine/tyrosine antibody (Cat. No. 61-8300, Thermo Fisher Scientific)
-a rabbit Control IgG antibody (Cat. No. AC005, Abclonal, China)
-a chicken anti-GFP antibody (Cat. No. ab13970, Abcam)
-an Alexa Fluor 488-conjugated goat anti-chicken IgY secondary antibody (Cat. No. ab150169, Abcam)

Validation

Information for antibodies for this study are,
-a rabbit anti-human CD41/ITGA2B antibody (Cat. No. A11836, Abclonal, China)
https://abclonal.com.cn/catalog/A11836
-an Alexa Fluor 647-conjugated donkey anti-rabbit antibody (Cat. No. A31573, Invitrogen)
https://www.thermofisher.cn/cn/zh/antibody/product/Donkey-anti-Rabbit-IgG-H-L-Highly-Cross-Adsorbed-Secondary-Antibody-Polyclonal/A-31573
-a goat anti-mouse HRP-conjugated IgG antibody (Cat. No. M21001L, Abmart. Shanghai, China)

https://www.ab-mart.com.cn/page.aspx?node=%2062%20&id=%20960
-a goat anti-rabbit HRP-conjugated IgG antibody (Cat. No. M21002L, Abmart. Shanghai, China)
https://www.ab-mart.com.cn/page.aspx?node=%2062%20&id=%20980
-a rabbit anti-GP6 antibody (Cat. No. ab302950, Abcam)
https://www.abcam.cn/products/primary-antibodies/gpvi-antibody-epr25690-71-ab302950.html
-a rabbit anti-P2RY12 antibody (Cat. No. 11976-1-AP, Proteintech)
https://www.ptgcn.com/products/P2RY12-Antibody-11976-1-AP.htm
-a rabbit anti-SLC6A8 antibody (Cat. No. 20299-1-AP, Proteintech)
https://www.ptgcn.com/products/SLC6A8-Antibody-20299-1-AP.htm
-a mouse anti-CKB antibody (Cat. No. 66764-1-Ig, Proteintech)
https://www.ptgcn.com/products/CKB-Antibody-66764-1-Ig.htm
-a rabbit anti-GATM antibody (Cat. No. 12801-1-AP, Proteintech)
https://www.ptgcn.com/products/GATM-Antibody-12801-1-AP.htm
-a rabbit anti-STAT5B antibody (Cat. No. 34662, Cell Signaling Technology)
https://www.cellsignal.cn/products/primary-antibodies/stat5b-antibody/34662
-a rabbit anti-Phospho-STAT5 antibody (Cat. No. AP0138, ABclonal)
https://abclonal.com.cn/catalog/AP0138
-a rabbit anti-Histone H3 antibody (Cat. No. 4499, Cell Signaling Technology)
https://www.cellsignal.cn/products/primary-antibodies/histone-h3-d1h2-xp-rabbit-mab/4499
-a mouse anti-β-actin antibody (Cat. No. 66009-1-Ig, Proteintech)
https://www.ptgcn.com/products/Pan-Actin-Antibody-66009-1-Ig.htm
-a mouse anti-FLAG antibody (Cat. No. F3165, Sigma-Aldrich)
https://www.sigmaaldrich.cn/CN/zh/product/sigma/f3165
-a rabbit anti-Phosphoserine/threonine/tyrosine antibody (Cat. No. 61-8300, Thermo Fisher Scientific)
https://www.thermofisher.cn/cn/zh/antibody/product/Phosphoserine-threonine-tyrosine-Antibody-Polyclonal/61-8300
-a rabbit Control IgG antibody (Cat. No. AC005, Abclonal, China)
https://abclonal.com.cn/catalog/AC005
-a chicken anti-GFP antibody (Cat. No. ab13970, Abcam)
https://www.abcam.cn/products/primary-antibodies/gfp-antibody-ab13970.html
-an Alexa Fluor 488-conjugated goat anti-chicken IgY secondary antibody (Cat. No. ab150169, Abcam)
https://www.abcam.cn/products/secondary-antibodies/goat-chicken-igy-hl-alexa-fluor-488-ab150169.html

Eukaryotic cell lines

Policy information about cell lines and Sex and Gender in Research

Cell line source(s)

Murine B16-F10-EGFP melanoma and MC38 colorectal cancer cell lines were purchased (Cat. No. GZQ0031, Shanghai Zhong Qiao Xin Zhou Biotechnology, China; Cat. No. QuiCell-M545, Shanghai QuiCell Biotechnology Co., Ltd. &Technology, China). Murine Lewis lung carcinoma lung cancer and 4T1 breast cancer cell lines were kindly provided by Prof. Yihai Cao from the Karolinska Institute, Sweden. Human embryonic kidney 293T (HEK 293T) cell line was kindly provided by Prof. Yongbo Wang from the Fudan University, China. Human Jurkat T cell line was kindly provided by Prof. Dapeng Yan from the Fudan University, China.Human MEG-01 cell line was provided by Dr. Xiangzhong Zhao from the central laboratory, the Affiliated Hospital of Qingdao University, Qingdao, China.

Authentication

Purchased cell lines were authenticated by short tandem repeat (STR) DNA profiling.

Mycoplasma contamination

All cell lines used in our study were negative for mycoplasma confirmed by a PCR method with 2 primer pairs: forward: 5'-GGCGAATGGGTGAGTAACACG-3' and reverse: 5'-CGGATAACGCTTGCGACCTATG-3'; forward: 5'-GGGAGCAAACAGGATTAGATACCCT-3' and reverse: 5'-TGCACCATCTGTCACTCTGTTAACCTC-3'.

Commonly misidentified lines
(See ICLAC register)

No commonly misidentified cell line was used.

Animals and other research organisms

Policy information about studies involving animals; ARRIVE guidelines recommended for reporting animal research, and Sex and Gender in Research

Laboratory animals

This study utilized laboratory mice as an in vivo animal model. Male C57BL/6J mice (Cat. No. N000013) and female BALB/cJ (Cat. No. N000020) at the age between 6-8-week-old were purchased from the GemPharmatech, China and maintained under 12-h dark/12-h light cycle with food and water provided ad libitum. To obtain MK-specific Slc6a8 knockout mice, Slc6a8flox/flox mice (Cat. No. T019665, GemPharmatech, China) were crossed with Pf4 Cre mice (Cat. No. C001050, Cyagen, China) to obtain Pf4 Cre; Slc6a8flox/flox mice.

Wild animals

This study did not use wild animals.

Reporting on sex

No sex differences were emphasized in this study. Both male and female mice were used in the experiments. The specific gender was indicated in each experiment.The influence of sex was not evaluated in this study.

Field-collected samples

This study did not collect samples from the field.

Ethics oversight | All animal experiments were approved by the Animal Experimental Ethical Committee of Fudan University, Shanghai, China (20220311-002)

Note that full information on the approval of the study protocol must also be provided in the manuscript.

Plants

Seed stocks | n/a

Novel plant genotypes | n/a

Authentication | n/a

Flow Cytometry

Plots

Confirm that:

- [x] The axis labels state the marker and fluorochrome used (e.g. CD4-FITC).
- [x] The axis scales are clearly visible. Include numbers along axes only for bottom left plot of group (a 'group' is an analysis of identical markers).
- [x] All plots are contour plots with outliers or pseudocolor plots.
- [x] A numerical value for number of cells or percentage (with statistics) is provided.

Methodology

Sample preparation | For CTC detection, blood samples were collected, transferred to an anticoagulation tube, and centrifuged at 500 g for 5 min. Cells were resuspended in RBC lysis buffer (Cat. No. MA0207, Meilunbio, China) for 5 min to remove RBCs.
For the MK polyploidy test, isolated MKs were stained with Hoechst 33342 (Cat. No. IH0070, Solarbio, China) for 30 min at room temperature.

Instrument | Flow Cytometer CytoFLEX S (Beckman Coulter) for CTC detection; FACS canto II (BD Bioscience) for MK polyploidy test.

Software | CytExpert software (Beckman Coulter).

Cell population abundance | We shorted GFP positive cell population and CD41 positive cell population.

Gating strategy | FSC/SSC gate for the elimination of debris followed by a gate for GFP+ tumor cells isolation.

- [ ] Tick this box to confirm that a figure exemplifying the gating strategy is provided in the Supplementary Information.
