# Supplementary material for: Exogenous creatine supplementation promotes tumor metastasis via megakaryocyte creatine kinase B-STAT5B signaling
Source: Nat Commun. 2026 Jul 15;17:8662. doi: 10.1038/s41467-026-74639-z (PMC13490413; doi:10.1038/s41467-026-74639-z)
Supplement: Supplementary file 4 [file 41467_2026_74639_MOESM5_ESM.pdf]

nature portfolio

Peer Review File

Exogenous creatine supplementation promotes tumor metastasis via megakaryocyte creatine kinase B-STAT5B signaling

Corresponding Author: Professor Yunlong Yang

Parts of this Peer Review File have been redacted as indicated to remove third-party material.

This file contains all reviewer reports in order by version, followed by all author rebuttals in order by version.

Version 0:

Reviewer comments:

Reviewer #1

(Remarks to the Author)
In this manuscript, Chen et al address an important and timely topic with potential implications for our understanding of creatine biology and its role in promoting metastasis. The study also provides insights into metabolic modulation in the context of platelet function and signalling. The authors found that creatine modifies platelet and megakaryocyte biology, regulates CKB activity and the phosphorylation of STAT5B at S126. Inhibition of STAT5B in MK and with pharmacological inhibitors decreased metastasis induced by creatine supplementation. A strong aspect of the study is that the effects of creatine in regulating platelet functions wer confirmed in human. The experimental design is sound overall, and the findings are of interest. However, several aspects of the manuscript would benefit from greater clarity. My detailed comments are outlined below.

Major points
1. The results do not show that CKB directly phosphorylates STAT5B, as claimed in the study. The data instead show that STAT5B phosphorylation may be regulated downstream of CKB but the involvement of a kinase mediating this phosphorylation cannot be ruled out from the data shown in the paper. To show potential for direct phosphorylation, the authors should carry out in vitro assays with purified STAT5 and CKB.
2. Regarding the phosphoproteomic experiment:
• Fig 5A shows the results of the phosphoproteomic experiments that identified STAT5B phosphorylation increased by creatine. Which sites on STATB etc were found to be differentially phosphorylated in this analysis?
• Related to the precious point, 5813 differentially abundant phosphorylated peptides do not match the volcano plot in Figure 5A.
• The authors should provide the phosphoproteomics data in a supplementary table for review and submitted to an appropriate repository to ensure transparency and reproducibility.
• In addition, the authors should comment on the statistical cut offs they have used to determine significance of differential phosphorylation and state if p-value adjustment was carried to correct for multiple testing.
• Have the authors performed broader enrichment analyses to provide a global perspective on the phosphoproteomics dataset?
• Does the MS data support increased activity of CKB or increased phosphorylation of STAT5B at S127
3. Whilst there is compelling evidence for a role of creatine in cancer metastasis, the authors solely focus on the impact of exogenous creatine. It remains to be seen whether targeting endogenous creatine levels would have any impact. Whilst this is touched on in the discussion, this statement should be clarified.
4. A limitation in the study is that it aims to understand creatine's effects in cancer metastasis. However, while cancer is predominantly a disease of older people, all human blood samples used in the study as controls were from young, healthy donors. This raises questions about generalizability to cancer patients, whose metabolic profiles and age may differ substantially. The authors should more clearly acknowledge this.

Minor points
5. Page 5, Line 20: Please clarify what is meant by "cascades." Are you referring to signalling cascades? Also, mention of

platelets needs context, are they involved in the pathways discussed?
6. Statistical results are reported without stating the statistical tests used or providing significance values. Please include these for clarity and rigor. This comment applies to many other statements in this paper.
7. The effect seen in the flow data appears numerically very small (just 0.3%). Could the authors comment on the biological relevance of this finding?
8. The dose of aspirin is not provided in the main text, nor is the rationale for the selected dose explained.
9. Across all figures, please include details of the statistical tests used. This is currently missing.
10. Label for Figure 2, panel G is misaligned.
11. The docking relies on an AlphaFold predicted structure (that of STAT5B), can the authors comment on the reliability of this structure?
12. In the discussion section, please Replace “inhibits” with “inhibit” for grammatical accuracy (Page 20, Line 9) and please review and correct grammatical errors for improved clarity and readability.
13. In the methods section:
• Page 25, Line 18: Plasma collection is typically done between 2 and 8°C to preserve sample integrity. The stated 20°C is unusual—please justify or correct this.
• Page 26, Line 12: The authors state some of the experiments where carried out in low-glucose media, could they reference the specific experiments.
• Page 29, Line 12: What was aspirin dissolved in?
• Page 35: The STAT5B structure used is a predicted structure from AlphaFold. Please discuss the reliability of this model and whether it has been validated experimentally or computationally.

Reviewer #2

(Remarks to the Author)
I co-reviewed this manuscript with one of the reviewers who provided the listed reports. This is part of the Nature Communications initiative to facilitate training in peer review and to provide appropriate recognition for Early Career Researchers who co-review manuscripts.

Reviewer #3

(Remarks to the Author)
In this manuscript, Chen et al. detail a novel mechanism through which creatine supplementation impacts megakaryocytic production of active platelets and the implications that activation of this pathway could have on tumor metastasis in creatine users. Overall, the construction of this investigation is well-done, convincing, and generally impressive; however, there are several issues throughout the manuscript that need the authors' attention.

First, I recommend a comprehensive and scrutinizing review of the formatting, styling and language used throughout the paper. While much of these composition errors are largely cosmetic and detract from the quality of the paper superficially, there are a substantial amount of language errors that significantly obstruct the interpretability of the manuscript. Errors in clarity that inhibit the manuscript's ability to communicate its findings can be found throughout the paper in the introduction, results, discussion, methods, and figure legends, and the authors should edit or contract an editor to go through the entire manuscript line-by-line.

Additionally, I have highlighted below several points throughout the paper that need specific amendment or clarification by the authors before their findings can be published:

9.12-14, Fig 1G, 1H, S2B: “At 5 min after injection, GFP+ tumor cells accounted for approximately 6% of circulating nucleated cells.” Figures 1H and S2B have the first timepoint listed as .25 hours. In Fig 1G, the time point of 3h is depicted despite no 3h time point in analyses but 5m (or 15m) and 6h are absent.

11.7-9, Fig S3A: “we further investigated the RNA and protein expression of various genes associated with platelet receptors and granules in freshly isolated bone marrow MKs using a density gradient centrifugation-based method” Structure of sentence implies RNA and protein expression was measured directly by density gradient. Authors also need to state that mRNA expression was measured via qPCR, both in results and figure legend (as was done in Fig. S2A, 4, S5A). Additionally, the labels for the gates in the first two panels of Fig. S3A are inaccurate and need to be corrected.

13.18-20: “compared to brain tissue, which is known to store high levels of creatine and express high levels of CKB, bone marrow MKs exhibited similar levels of intracellular creatine and expressed similar levels of CKB upon creatine supplementation (Fig. 4A and B).” In the structure of figures 4A-4C, it is odd to exclude phosphocreatine levels in brain tissue, as it has been used as a control for a creatine-synthesizing tissue largely independent of exogenous creatine levels to contextualize the creatine-importing behaviors observed in MK. Was there a specific reason for excluding PCr levels in brain tissue?

15.24-16.2: “phospho-mutant at site S127 (S127A), and a triple phospho-mutant at site S127 and two other canonical sites (S127A, Y699A, and S731A, designated 3A) were not detected by phosphorylation antibody in either vehicle- or creatine-treated cells (Fig. 5E and F).” Why was it decided to use the 3A mutant instead of a Y699A or “2A” (Y699A + S731A) mutant? Was this simply included to show that there was no clear additive effect surpassing the S127A IP ablation when

other candidate binding sites were mutated? Because quantifications of protein expression in WB are not given, the results of Fig. 5E have an opposite effect on confidence in S127 as the site of phosphorylation, especially in the lack of single-residue mutation controls for the ”2A” sites. The data is otherwise convincing and Fig. S6A helps dispel Y699 as a candidate CKB binding site, but the 3A mutant offers little rhetorically constructive benefit and could perhaps even introduce unnecessary doubt. If the original reasoning for inclusion of 3A greatly diverges from this, it should be clearly articulated in the results section.

20.9-10: “Creatine has been reported to directly inhibits cancer cells, or to suppress tumor growth via upregulating cytolytic T cell activity.” Citations needed. Discussing in greater detail the implications that these previously-described effects could have on the recommendation considerations of creatine for patients with cancer in concert with these new findings is the type of greater contextualization that the discussion section is meant for.

Reviewer #4

(Remarks to the Author)
This manuscript by Chen et al. presents a comprehensive and well-designed series of experiments demonstrating that exogenous creatine supplementation promotes tumor metastasis through megakaryocyte-mediated platelet hyperactivity. The authors establish a novel mechanistic pathway involving SLC6A8-mediated creatine uptake, Creatine Kinase B upregulation, and non-canonical STAT5B (S127) phosphorylation, which drives expression of platelet activation genes. The work is technically rigorous, employing conditional knockout models, pharmacological inhibition, adoptive platelet transfer, and multi-cancer metastasis assays. The discovery that Creatine Kinase B has a kinase function and not just a metabolic role is particularly striking considering that creatine biology in pathological status remains poorly understood. The inclusion of a pilot human supplementation study adds translational relevance, though the small sample size and use of circulating CD41+ cells as MK surrogates warrant caution. Further, mechanisms of platelet-dependent promotion of tumor metastasis are not explored, nor is a clinical evidence of creatine assumption and cancer risk in human studies.

Major:
- It is not clear whether CKB expression and activity in Megakaryocytes differ from other bone marrow leukocytes or even platelets.
- CKB inhibitors should be tested, at least in vitro.
- I have technical concerns about megakaryocyte analyses. Characterization of bone marrow isolated megakaryocytes should be provided. DNA content analysis included in Sup. Fig. 3A is not convincing. It seems that cells display a very low ploidy level. Is this the case?
- Similarly, the strategy of using CD41 cells isolated from peripheral blood of healthy subjects in human studies may not reflect megakaryocyte biology of the bone marrow. Additionally, cell identity used in this assay should be assessed. It is known that circulating platelets may attach to leukocytes, contaminating microbead separation strategies.
- What is the difference between “isolated MKs” and “isolated MKs in vitro”? Please specify.
- Sup. Fig. 5A shows that creatine supplementation does not affect mRNA levels of GATM and GAMT. However, the contribution of de novo creatine synthesis in these settings is not convincingly excluded.
- Platelets transfer experiments support the hypothesis of a mechanism restricted to the MK/plt lineage. I would suggest including any evidence that in vivo creatine supplementation in mice has no impact on other hematopoietic lineages.
- Figure 7. Western blot on before and after creatine supplementation platelets is missing.

Reviewer #5

(Remarks to the Author)
REVIEW: Creatine metastasis

Summary
Oral creatine at clinically relevant levels slowed clearance of intravenously injected tumor cells, increased lung metastases, and shortened survival across multiple mouse tumor models and both sexes; the effect was independent of tumor-cell SLC6A8, transferable by platelets, and attenuated by anti-platelet agents: aspirin and anagrelide. Mechanistically, creatine acts on megakaryocytes via SLC6A8 to produce hyperactive, tumor-adherent platelets—upregulating receptors (P2Y12, GPVI) and granule programs—through creatine-driven CKB upregulation that promotes non-canonical STAT5B phosphorylation, nuclear translocation, and transcription of platelet activation genes. MK-specific deletion of Slc6a8 or Stat5b, and pharmacologic STAT5 inhibition, abolishes effects. In humans, short-term creatine supplementation increases platelet activity and MK/platelet expression of P2RY12/GP6, and creatine-primed human platelets enhanced metastasis when xenotransferred into immunosuppressed mice.
This work is important, interesting, and well executed. There are some important questions that could significantly enhance the work.

Questions
1. What happens in other metastatic models in addition to tail vein: intracardiac, or spontaneous?
2. Anagrelide reduces MK maturation and proplatelet formation, and its role requires clarification in this context (not platelet activation). Have the authors tried platelet depletion strategies, or conversely, antagonising acethycholine receptor to enhance platelet activation (opposite effect control) to specifically show this is activation mediated and not just thrombocytopaenia - ie via MK maturation effect?
3. Similarly, COX inhibition is broad range effective. Will aspirin/anagrelide given to recipient mice, not just ex vivo–treated

donor platelets impact metastasis, and show impact on target TXB2 suppression in vivo.
4. could the authors show CTC aggregates with a marker for platelet activity in blood, for example p-selectin activation + GFP cells. similarly, they should rule out effects on lung fibrin by creatinine (d-dimer?), which could lead to more CTC trapping instead of platelet activation peripherally.
5. Please present RNA data to rule out platelets are contaminant in the MK RNA and protein assays.
6. does re expression of SLC6A8 (or creatine bypassing transporter) restore the phenotype?
7. are the human data stratified for NSAID intake.
8. The authors need further work on the CKB/stat5b pathway, including in vitro assay KD CKB controls, of pS127 antibody. They need to show this is jak independent, that canonical y699 stat5 is not involved. could they do a crispr of other ko stat5b with wt v s127a in MKs to test s127 is necessary for genetic induction and platelet activity.

Version 1:

Reviewer comments:

Reviewer #1

(Remarks to the Author)
The authors have addressed the points I made to the previous version of the manuscript, and I have no further comments. In my view, the article is ready for publication.

Reviewer #2

(Remarks to the Author)
I co-reviewed this manuscript with one of the reviewers who provided the listed reports. This is part of the Nature Communications initiative to facilitate training in peer review and to provide appropriate recognition for Early Career Researchers who co-review manuscripts.

Reviewer #3

(Remarks to the Author)
The revised manuscript appears to satisfactorily address the majority of the requests by myself and the other reviewers. With respect to my suggestions, the authors cover the gaps and update the preciseness of their language to better convey their results, as well as address and revise the inconsistencies in their experimental design or presentation.

Reviewer #5

(Remarks to the Author)

Reviewer #6

(Remarks to the Author)
Chen et al. show that exogenous creatinine supplementation promotes tumor metastasis via a previously unrecognized megakarocyte-mediated pathway leading to platelet hyperactivity. There studies are highly mechanistic and include a range of complementary strategies including in vitro and in vivo studies that leverage both pharmacological and genetic approaches. A small pilot study in humans extends the scope and relevance of their findings. The investigators have responded thoroughly to the prior critique of their manuscript and have provided appropriate clarifications and verifications of their initial report.

Open Access This Peer Review File is licensed under a Creative Commons Attribution 4.0 International License, which permits use, sharing, adaptation, distribution and reproduction in any medium or format, as long as you give appropriate credit to the original author(s) and the source, provide a link to the Creative Commons license, and indicate if changes were made.
In cases where reviewers are anonymous, credit should be given to 'Anonymous Referee' and the source.
The images or other third party material in this Peer Review File are included in the article's Creative Commons license, unless indicated otherwise in a credit line to the material. If material is not included in the article's Creative Commons license and your intended use is not permitted by statutory regulation or exceeds the permitted use, you will need to obtain permission directly from the copyright holder.
To view a copy of this license, visit https://creativecommons.org/licenses/by/4.0/

Our point-by-point responses to the reviewers' comments are as follows:

Reviewer: 1

Comment: In this manuscript, Chen et al address an important and timely topic with potential implications for our understanding of creatine biology and its role in promoting metastasis. The study also provides insights into metabolic modulation in the context of platelet function and signalling. The authors found that creatine modifies platelet and megakaryocyte biology, regulates CKB activity and the phosphorylation of STAT5B at S126. Inhibition of STAT5B in MK and with pharmacological inhibitors decreased metastasis induced by creatine supplementation. A strong aspect of the study is that the effects of creatine in regulating platelet functions wer confirmed in human. The experimental design is sound overall, and the findings are of interest. However, several aspects of the manuscript would benefit from greater clarity. My detailed comments are outlined below.

Response: We thank the reviewer for correctly understanding our work and considering our topic important and timely. We appreciate the reviewer's insightful comments, which are important to improve the quality of our work.

Comment: Major points 1. The results do not show that CKB directly phosphorylates STAT5B, as claimed in the study. The data instead show that STAT5B phosphorylation may be regulated downstream of CKB but the involvement of a kinase mediating this phosphorylation cannot be ruled out from the data shown in the paper. To show potential for direct phosphorylation, the authors should carry out in vitro assays with purified STAT5 and CKB.

Response: We thank the reviewer for raising this point. We agree with the reviewer that our data only suggest that CKB phosphorylates STAT5B, but do not exclude the involvement of other potential kinases in the phosphorylation of STAT5B. Following the reviewer's suggestion, we have conducted in vitro assays with purified STAT5B and CKB.

For in vitro kinase assay, wildtype FLAG-STAT5B was purified from 293T cells using anti-FLAG Magarose beads and DynaMag-2 magnets. Bacterially purified wildtype His-CKB (0.4 μg/μL) was incubated with FLAG-STAT5B (1 μg/μL) in the presence of 0.5 μM ATP in a commercially available Kinase Buffer (25 mM Tris-HCl (pH 7.5), 5 mM β-glycerophosphate, 2 mM DTT, 0.1 mM Na_3VO_4, 10 mM $MgC1_2$) (Fig. R1A).

After incubation for 1 h, FLAG-STAT5B was purified from the Kinase Buffer and was tested for phosphorylation. As expected, FLAG-STAT5B that not co-incubated with CKB showed low phosphorylation signal, while FLAG-STAT5B co-incubated

with CKB in vitro showed a strong phosphorylation (Fig. R1B), suggesting that CKB directly phosphorylates STAT5B.

Of note, although our results provide evidence that CKB directly phosphorylates STAT5B, these results do not exclude the possibility that other proteins may be involved in the STAT5B phosphorylation process in cells. We further discussed this point in the Discussion section. We thank the reviewer for this important comment.

REDACTED

Fig. R1 (A) Schematic diagram of the in vitro kinase assay. FLAG-STAT5B and His-CKB were co-incubated in a kinase buffer containing ATP. FLAG-STAT5B was then purified for the phosphorylation test. (B) Western blot of phosphorylation of the STAT5B-FLAG co-incubated with or without His-CKB. FLAG marks the loading amount in each lane.

Some of the results have been incorporated into the revised manuscript (New Fig S6C and D)

Comment: 2. Regarding the phosphoproteomic experiment:

• Fig 5A shows the results of the phosphoproteomic experiments that identified STAT5B phosphorylation increased by creatine. Which sites on STATB etc were found to be differentially phosphorylated in this analysis?

• Related to the precious point, 5813 differentially abundant phosphorylated peptides do not match the volcano plot in Figure 5A.

• The authors should provide the phosphoproteomics data in a supplementary table for review and submitted to an appropriate repository to ensure transparency and reproducibility.

• In addition, the authors should comment on the statistical cut offs they have used to determine significance of differential phosphorylation and state if p-value adjustment was carried to correct for multiple testing.

• Have the authors performed broader enrichment analyses to provide a global perspective on the phosphoproteomics dataset?

• Does the MS data support increased activity of CKB or increased phosphorylation of STAT5B at S127

Response: Completely agree. To increase the clarity of our phosphoproteomic data, we re-analyzed our MS data and revised the description for clarity.

(1) Analysis of the phosphoproteomics dataset confirmed that there is only one site on STAT5B, S127, that was differentially phosphorylated (Supplementary Table 2).

(2) Fig 5A displays a volcano plot of 5812 differentially phosphorylated peptides. To improve the clarity, we revised the figure legend to: "Volcano plot of 5812 differentially phosphorylated peptides in freshly isolated MKs from vehicle- or creatine-treated mice (n = 3 mice per group)".

(3) Completely agree. As indicated in the revised manuscript, the phosphoproteomics dataset is provided in Supplementary Table 3, and the raw phosphoproteomics data have been deposited in the PRIDE database under accession number PXD065649.

(4) Completely agree. In MS analysis, the screening criteria for differential peptides were set as follows: Fold change ≥1.2 or ≤0.83, with a P value or P value from chi-square test <0.05. This information has been added to the Methods section of the revised manuscript.

(5) We thank the reviewer for this insightful comment. KEGG and GO enrichment analyses have been performed, and the corresponding results are presented (Fig. R2A-B). Of note, mRNA processing pathways were enriched, which possibly reflect the RNA expression changes in creatine-treated MKs.

(6) Phosphorylated CKB was not detected in our dataset. MS data support enhanced phosphorylation of STAT5B at the S127 site, but provide no evidence for increased CKB activity.

Fig.R2

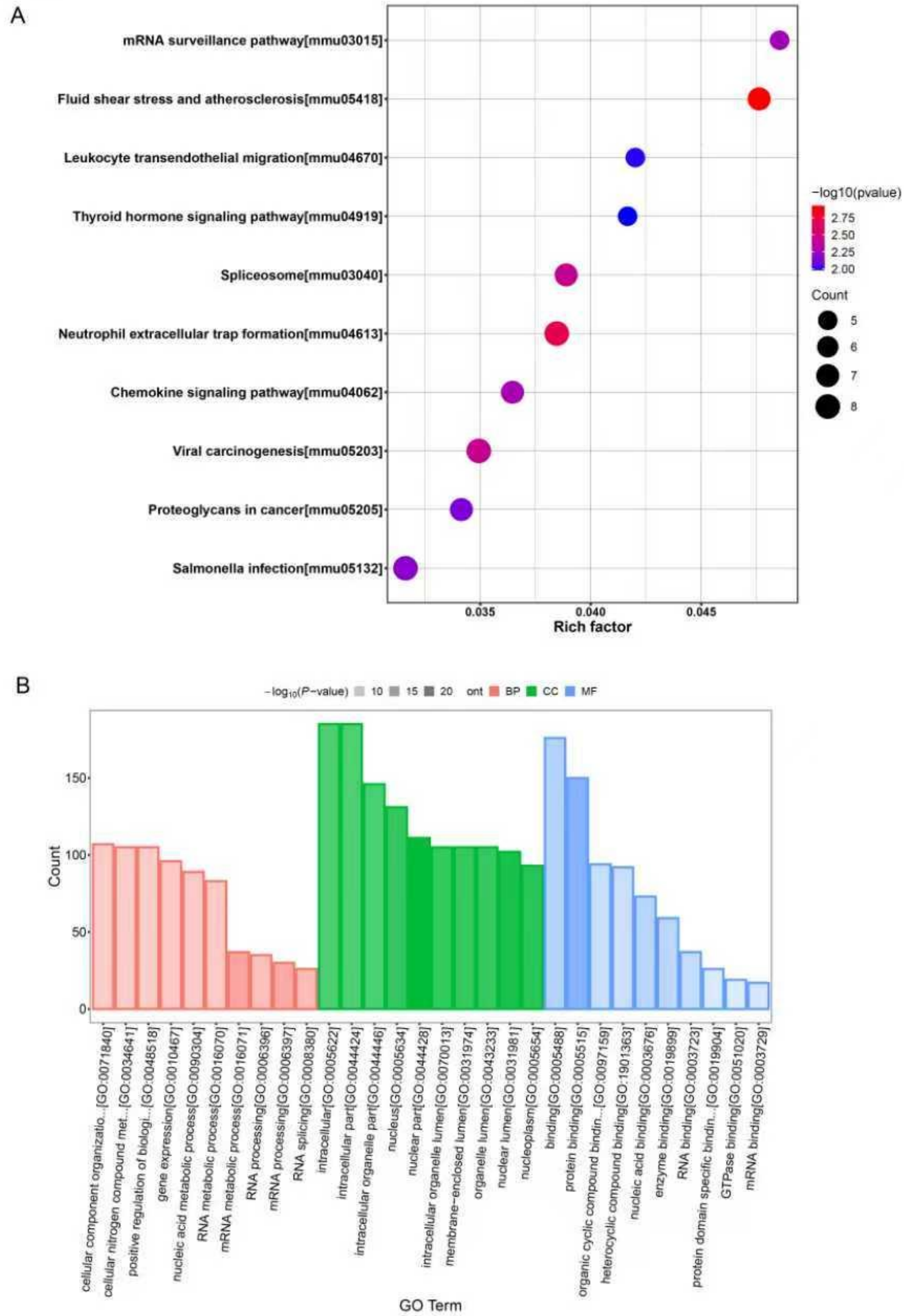

Fig. R2 (A) KEGG pathway enrichment analysis of phosphoproteomic data. Bubble plot showing enriched KEGG pathways. Rich Factor is plotted on the x-axis. Bubble size is proportional to the count of mapped differential phosphoproteins. Bubble color intensity (red scale) indicates the enrichment significance (lower p-value). (B) Gene Ontology (GO) enrichment analysis of phosphoproteomic data. Bar colors denote GO categories: Biological Process (BP, red), Cellular Component (CC, green), Molecular

Function (MF, blue). Bar height shows the count of mapped differential phosphoproteins per term. Bar transparency (darker shade) indicates higher statistical significance (lower p-value).

Comment: 3. Whilst there is compelling evidence for a role of creatine in cancer metastasis, the authors solely focus on the impact of exogenous creatine. It remains to be seen whether targeting endogenous creatine levels would have any impact. Whilst this is touched on in the discussion, this statement should be clarified.

Response: Completely agree. Given the growing popularity of creatine supplements around the world, our focus is on the impact of exogenous creatine. The impact of endogenous creatine may differ from that of exogenous creatine supplementation, since the former requires the activation of the creatine biosynthesis pathway and consumes glycine and arginine. In the revised manuscript, we have made this point clear in the Discussion section.

Comment: 4. A limitation in the study is that it aims to understand creatine's effects in cancer metastasis. However, while cancer is predominantly a disease of older people, all human blood samples used in the study as controls were from young, healthy donors. This raises questions about generalizability to cancer patients, whose metabolic profiles and age may differ substantially. The authors should more clearly acknowledge this.

Response: Completely agree. Our human study has limitations since we cannot recruit patients with cancer for this type of study. Patients with cancer may have different metabolic profiles and respond differently to exogenous creatine supplements than young, healthy adults. In the revised manuscript, this limitation has been clearly stated in the Discussion section.

Comment: Minor points

5. Page 5, Line 20: Please clarify what is meant by "cascades." Are you referring to signalling cascades? Also, mention of platelets needs context, are they involved in the pathways discussed?

6. Statistical results are reported without stating the statistical tests used or providing significance values. Please include these for clarity and rigor. This comment applies to many other statements in this paper.

7. The effect seen in the flow data appears numerically very small (just 0.3%). Could the authors comment on the biological relevance of this finding?
8. The dose of aspirin is not provided in the main text, nor is the rationale for the selected dose explained.
9. Across all figures, please include details of the statistical tests used. This is currently missing.
10. Label for Figure 2, panel G is misaligned.
11. The docking relies on an AlphaFold predicted structure (that of STAT5B), can the authors comment on the reliability of this structure?
12. In the discussion section, please Replace "inhibits" with "inhibit" for grammatical accuracy (Page 20, Line 9) and please review and correct grammatical errors for improved clarity and readability.
13. In the methods section:
• Page 25, Line 18: Plasma collection is typically done between 2 and 8°C to preserve sample integrity. The stated 20°C is unusual—please justify or correct this.
• Page 26, Line 12: The authors state some of the experiments where carried out in low-glucose media, could they reference the specific experiments.
• Page 29, Line 12: What was aspirin dissolved in?
• Page 35: The STAT5B structure used is a predicted structure from AlphaFold. Please discuss the reliability of this model and whether it has been validated experimentally or computationally.

Response:
5. The term "metastasis cascade" has been used by our group and others (PMID: 35307547, 39706193) to describe the sequential series of steps of the metastasis process. To improve the clarity, we have revised this sentence to "The cancer metastasis cascade is a multi-step process involving invasion, intravasation, circulation, extravasation, and colonization. Once cancer cells enter the bloodstream, platelets support circulating tumor cells (CTCs) by…"
6. We have revised the Methods section and figure legends to report the statistical methods used. We have also revised all the figures to show statistical values to improve the clarity.
7. One of the characteristics of CTCs is the extremely short half-life. Starting from 1960s, CTC measurements in various animal models by i.v. injection of labeled tumor cells have shown that, the number of living CTCs decreased rapidly to about 1% of cells after 24 h (PMID: 5513503). Recent study measuring kinetics of CTCs by advanced experimental setting have shown that, different infused tumor cells show a similar rapid clearance from the circulation, and only a small percentage (1−2%) of the injected cells remained detectable for 3 h (PMID: 34584084). Therefore, the numerically small flow data is reflecting the fact of extremely short half-life of infused tumor cells.

The survived CTCs represent a small but clinically significant pool for tumor progression. Clinically, the CTCs numbers are directly associated with the stages of the tumor (PMID: 35666958), therefore it is a valuable prognostic marker for patients with

cancer.
8. We apologize for not providing the detailed dose information in the main text. We chose the dose of 30 mg/kg as it is a clinically relevant dose and this dose is reported to be effectively inhibiting platelet activation in mice (PMID: 34535458). In the revised manuscript, we have provided the dose in the main text and added the relevant reference into the Methods section.
9. We have revised all figure legends to report the statistical methods used.
10. We thank the reviewer for helping us to improve the quality of the figures. This has been corrected.
11. Since we are only users but not experts on AlphaFold in predicting protein structure, we cannot comment the reliability of this prediction. Although we performed in vitro kinase assay to demonstrate that CKB directly phosphorylates STAT5B as predicted, we still do not know the real structure of STAT5B. Unlike STAT5A, STAT5B structure has not been characterized. We used STAT5A structure combined with AlphaFold in predicting protein structure. To address the reviewer's comment, we discussed this point in the Discussion section in the revised manuscript and emphasize that this structure is remaining to be validated. We thank the reviewer for his/her understanding.
12. We apologize for these grammar errors. We have reviewed and corrected grammatical errors throughout the text.
13.
- In general, our serum sample collection is done at 4 °C in the centrifugation. However, for platelet-rich plasma and platelet collection procedures, we performed at 20 °C of the room temperatures to prevent platelet activation and aggregation induced by the temperature elevation from 4°C to room temperature (PMID: 4254206).
- Low-glucose media were used in in vitro MK culture for making serum/glucose-starved MKs to test creatine effect in MKs. We have corrected the ambiguous sentence in the Method section.
- Aspirin was dissolved in drinking water for treating mice. We have revised this sentence in the Method section.
- According to the reviewer's suggestion, we have discussed the predicted structure of STAT5B, and emphasize that this structure is remaining to be validated.

We thank the reviewer for carefully evaluating our work. We appreciate the reviewer's constructive comments, which are important to improve the quality of our work.

Reviewer: 2

Comment: I co-reviewed this manuscript with one of the reviewers who provided the listed reports. This is part of the Nature Communications initiative to facilitate training in peer review and to provide appropriate recognition for Early Career Researchers who co-review manuscripts.

Response: We thank the co-reviewer for his/her effort in improving our work. We deeply appreciate it.

Reviewer: 3

Comment: In this manuscript, Chen et al. detail a novel mechanism through which creatine supplementation impacts megakaryocytic production of active platelets and the implications that activation of this pathway could have on tumor metastasis in creatine users. Overall, the construction of this investigation is well-done, convincing, and generally impressive; however, there are several issues throughout the manuscript that need the authors' attention.

Response: We thank the reviewer for considering our study is well-done, convincing, and generally impressive. It is encouraging for us.

Comment: First, I recommend a comprehensive and scrutinizing review of the formatting, styling and language used throughout the paper. While much of these composition errors are largely cosmetic and detract from the quality of the paper superficially, there are a substantial amount of language errors that significantly obstruct the interpretability of the manuscript. Errors in clarity that inhibit the manuscript's ability to communicate its findings can be found throughout the paper in the introduction, results, discussion, methods, and figure legends, and the authors should edit or contract an editor to go through the entire manuscript line-by-line.

Response: We thank the reviewer for raising this important issue. We apologize for the language errors. To improve the clarity, we have revised our manuscript line-by-line to clearly describe our findings, including explanation of terms, clarification of statistical methods, description of experimental methods, and a clearer description of our findings. We hope the revised manuscript is significantly improved for clarity.

Comment: Additionally, I have highlighted below several points throughout the paper that need specific amendment or clarification by the authors before their findings can be published:

9.12-14, Fig 1G, 1H, S2B: “At 5 min after injection, GFP+ tumor cells accounted for approximately 6% of circulating nucleated cells.” Figures 1H and S2B have the first timepoint listed as .25 hours. In Fig 1G, the time point of 3h is depicted despite no 3h time point in analyses but 5m (or 15m) and 6h are absent.

Response: We thank the reviewer for this comment. We apologize for this mistake. As the reviewer correctly predicted, “At 5 min after injection” should be revised as “At 15 min after injection”. The diagram in Fig. 1G should be revised accordingly. We have corrected the text and Fig 1G in the revised manuscript. We thank the reviewer for

improving our work.

Comment: 11.7-9, Fig S3A: "we further investigated the RNA and protein expression of various genes associated with platelet receptors and granules in freshly isolated bone marrow MKs using a density gradient centrifugation-based method" Structure of sentence implies RNA and protein expression was measured directly by density gradient. Authors also need to state that mRNA expression was measured via qPCR, both in results and figure legend (as was done in Fig. S2A, 4, S5A). Additionally, the labels for the gates in the first two panels of Fig. S3A are inaccurate and need to be corrected.

Response: We apologize for this misleading sentence. In the revised manuscript, we have revised the Results section for clarity. We have also stated the methods in measuring mRNA expression levels. We have clarified the labels in Fig. S3A.

Comment: 13.18-20: "compared to brain tissue, which is known to store high levels of creatine and express high levels of CKB, bone marrow MKs exhibited similar levels of intracellular creatine and expressed similar levels of CKB upon creatine supplementation (Fig. 4A and B)." In the structure of figures 4A-4C, it is odd to exclude phosphocreatine levels in brain tissue, as it has been used as a control for a creatine-synthesizing tissue largely independent of exogenous creatine levels to contextualize the creatine-importing behaviors observed in MK. Was there a specific reason for excluding PCr levels in brain tissue?

Response: We thank the reviewers for this knowledgeable comment. In the previous version of the manuscript, we used brain tissue as a known creatine-rich tissue control, and showed that MK cells have the similar creatine levels to brain tissue, at least after creatine-administration. We did intentionally exclude PCr levels in brain tissue.

Thanks to the reviewer, we now realize that brain tissues can also be used as a creatine-synthesizing tissue relatively independent of exogenous creatine levels, which allows us to contextualize the creatine-importing behaviors observed in MKs. Following the reviewer's suggestion, we performed PCr measurements in brain tissue from vehicle- or creatine-treated mice. As expected, after 7 days of creatine administration, PCr levels in brain tissue from creatine-treated mice increased moderately from 0.3 to 0.6 ng/μg. In contrast, PCr levels in MKs increased by approximately 5-fold (Fig. R3). Together with the creatine level results in Fig. 4A of the manuscript, these results support that brain is a creatine-synthesizing tissue that is relatively independent of exogenous creatine levels. MKs, however, depend on

exogenous creatine to increase intracellular creatine and PCr levels. We thank the reviewer for this valuable comment that improves the quality of our work.

Fig. R3

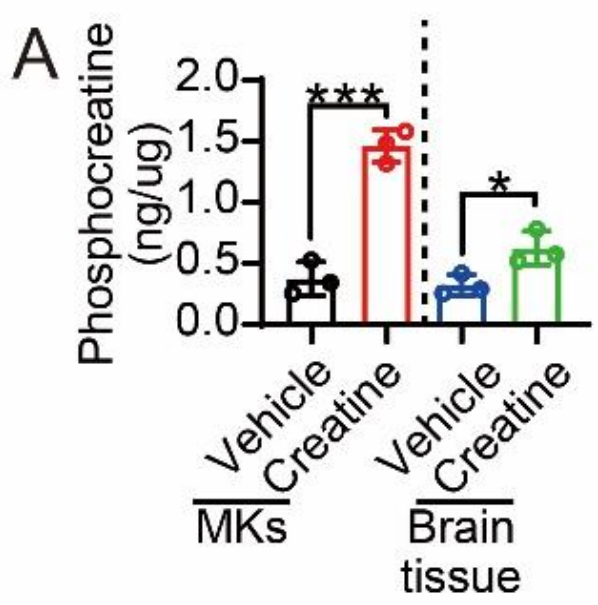

Fig. R3 (A) Intracellular phosphocreatine concentrations in freshly isolated MKs from vehicle- or creatine-treated mice (n = 3 mice per group). Brain tissues from the same group served as controls.

These results have been incorporated into the Fig. 4C in the revised manuscript.

Comment: 15.24-16.2: "phospho-mutant at site S127 (S127A), and a triple phospho-mutant at site S127 and two other canonical sites (S127A, Y699A, and S731A, designated 3A) were not detected by phosphorylation antibody in either vehicle- or creatine-treated cells (Fig. 5E and F)." Why was it decided to use the 3A mutant instead of a Y699A or "2A" (Y699A + S731A) mutant? Was this simply included to show that there was no clear additive effect surpassing the S127A IP ablation when other candidate binding sites were mutated? Because quantifications of protein expression in WB are not given, the results of Fig. 5E have an opposite effect on confidence in S127 as the site of phosphorylation, especially in the lack of single-residue mutation controls for the "2A" sites. The data is otherwise convincing and Fig. S6A helps dispel Y699 as a candidate CKB binding site, but the 3A mutant offers little rhetorically constructive benefit and could perhaps even introduce unnecessary doubt. If the original reasoning for inclusion of 3A greatly diverges from this, it should be clearly articulated in the results section.

Response: We thank the reviewer for this comment. The reviewer is completely correct. We simply included the 3A mutant to show that there was no additive effect surpassing the S127A mutant. In the revised manuscript, we added quantifications for this blot. These quantifications were analyzed from 3 independent Western blot gels. We have also clearly stated the reason for constructing the 3A mutant for clarity.

Fig. R4

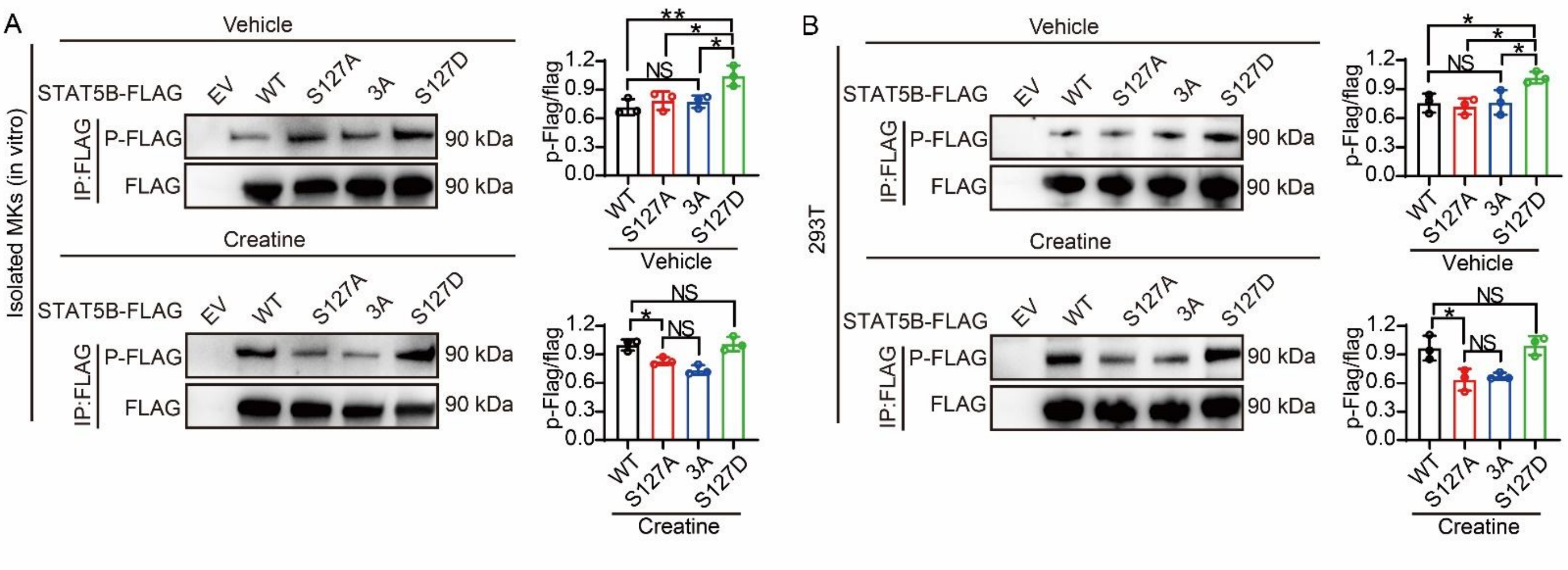

Fig. R11 (A) MKs were transfected with empty vector and STAT5B-FLAG vector with wt, S127A mutant, triple mutants, or S127D mutant. These groups were then treated with vehicle- or creatine in vitro. Western blot of phosphorylation of STAT5B-FLAG in various groups. FLAG marks the loading amount in each lane. Quantification from 3 independent blots. (B) 293T cells were transfected with empty vector and STAT5B-FLAG vector with wt, S127A mutant, triple mutants, or S127D mutant. These groups were then treated with vehicle- or creatine in vitro. Western blot of phosphorylation of STAT5B-FLAG in various groups. FLAG marks the loading amount in each lane. Quantification from 3 independent blots.

These results have been incorporated into the Fig. 5 in the revised manuscript.

Comment: 20.9-10: "Creatine has been reported to directly inhibits cancer cells, or to suppress tumor growth via upregulating cytolytic T cell activity." Citations needed. Discussing in greater detail the implications that these previously-described effects could have on the recommendation considerations of creatine for patients with cancer in concert with these new findings is the type of greater contextualization that the discussion section is meant for.

Response: Completely agree. We apologize for the missing reference and for not thoroughly discussing the current understanding of creatine-induced effects in patients with cancer. In the revised manuscript, we have added the missing reference and have discussed how our findings and the previous findings affect the recommendation of creatine in clinical settings. We have also discussed the limitations of the translational potential of our work. We thank the reviewer for improving the quality of our work.

Reviewer: 4

Comment: This manuscript by Chen et al. presents a comprehensive and well-designed series of experiments demonstrating that exogenous creatine supplementation promotes tumor metastasis through megakaryocyte-mediated platelet hyperactivity. The authors establish a novel mechanistic pathway involving SLC6A8-mediated creatine uptake, Creatine Kinase B upregulation, and non-canonical STAT5B (S127) phosphorylation, which drives expression of platelet activation genes. The work is technically rigorous, employing conditional knockout models, pharmacological inhibition, adoptive platelet transfer, and multi-cancer metastasis assays. The discovery that Creatine Kinase B has a kinase function and not just a metabolic role is particularly striking considering that creatine biology in pathological status remains poorly understood. The inclusion of a pilot human supplementation study adds translational relevance, though the small sample size and use of circulating CD41+ cells as MK surrogates warrant caution. Further, mechanisms of platelet-dependent promotion of tumor metastasis are not explored, nor is a clinical evidence of creatine assumption and cancer risk in human studies.

Response: We thank the reviewer for considering our work being well-designed and novel. We also thank the reviewer for recognizing that the pilot human supplementation study adds translational relevance. It is encouraging for us. We agree with the reviewer that our human study has limitations, to address this concern, we performed further experiments to address the MK identity issue, discussed the mechanisms of platelet-dependent promotion of tumor metastasis, and discussed the limitation of our human study.

Comment: Major:

- It is not clear whether CKB expression and activity in Megakaryocytes differ from other bone marrow leukocytes or even platelets.

Response: We thank the reviewer for this important comment. To address this comment, we examined the expression levels of megakaryocytes and other bone marrow cell types. In the Atlas of Mouse Blood Cells (http://scrna.sklehabc.com/mouse/), a single-cell RNA expression database for hematology research that includes various murine bone marrow cells such as leukocytes, erythrocytes, and megakaryocytes, we found that megakaryocytes showed the highest *Ckb* expression of all the examined bone marrow cell types (Fig. R5A). To validate this bioinformatic finding, *Ckb* expression in mouse megakaryocytes, CD19+ B cells, and CD3+ T cells was examined by qPCR. As expected, megakaryocytes exhibited higher *Ckb* expression compared with leukocytes (Fig. R5B). These results confirmed that CKB expression is higher in MKs than in other bone marrow leukocytes. We also compared the CKB expression in megakaryocytes and in platelets. As expected,

western blot revealed comparable CKB expression levels in both isolated megakaryocytes and peripheral platelets (Fig. R5C). These results confirmed that CKB expression is high in both megakaryocytes and in platelets.

Fig. R5

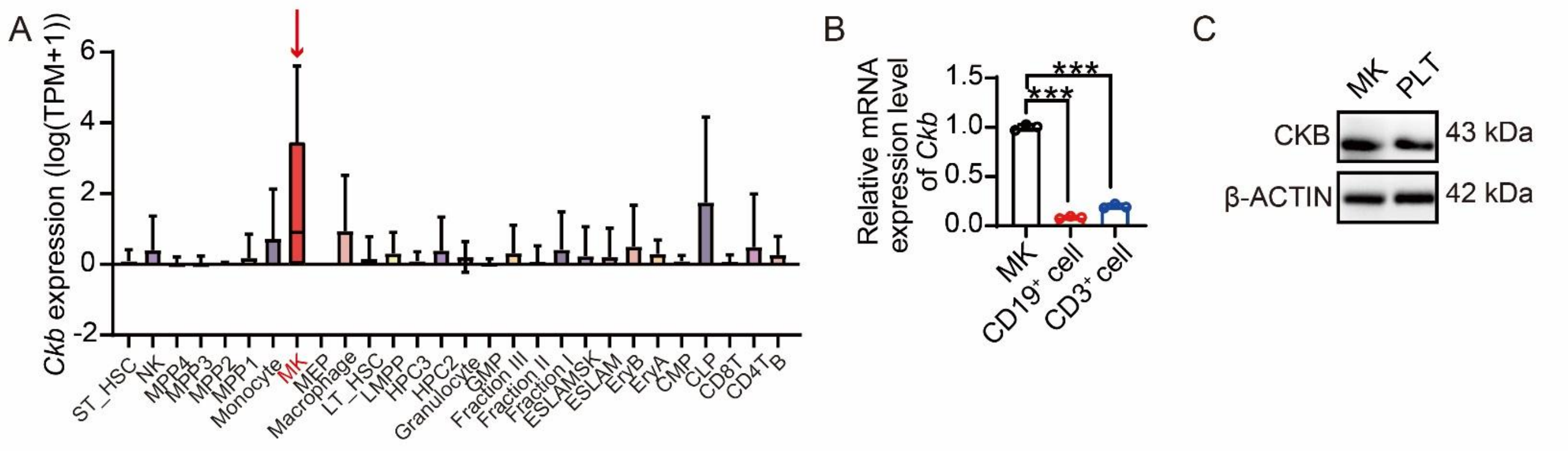

Fig. R5 (A) *Ckb* expression in various mouse bone marrow cells. Red arrow indicates MK. Data were collected from the Atlas of Mouse Blood Cells. NK, natural killer; LT-HSC, long-term hematopoietic stem cell; ESLAMSK, HSC (CD201+CD150+CD48−CD45+Sca-1+c-Kit+); ESLAM, HSC (CD150+CD48−CD45+EPCR+); ST-HSC, short-term hematopoietic stem cell; MPP, multipotent progenitor; LMPP, lymphoid-primed MPP; CLP, common lymphoid progenitor; HPC, hematopoietic progenitor cell; Ery, erythrocyte; MEP, megakaryocyte-erythrocyte progenitor; GMP, granulocyte-monocyte progenitor; CMP, common myeloid progenitor. (B) Quantitative PCR quantifications of *Ckb* expression in MKs, CD19+ cells, and CD3+ cells (n = 3 samples per group). (C) Western blot of CKB expression in MKs and platelets. β-actin indicates loading amount.

Comment: - CKB inhibitors should be tested, at least in vitro.

Response: Completely agree. We applied a CKB inhibitor BU99006, which selectively binds to the Cys283 in the enzyme’s active site and disrupts its catalytic activity. Similar to the si-*Ckb*-induced gene inhibition shown in Fig. 4J of the manuscript, the CKB inhibitor also blocked the creatine-stimulated expression of platelet activation-related genes (Fig. R6A), suggesting the expression of these genes depends on CKB catalytic activity.

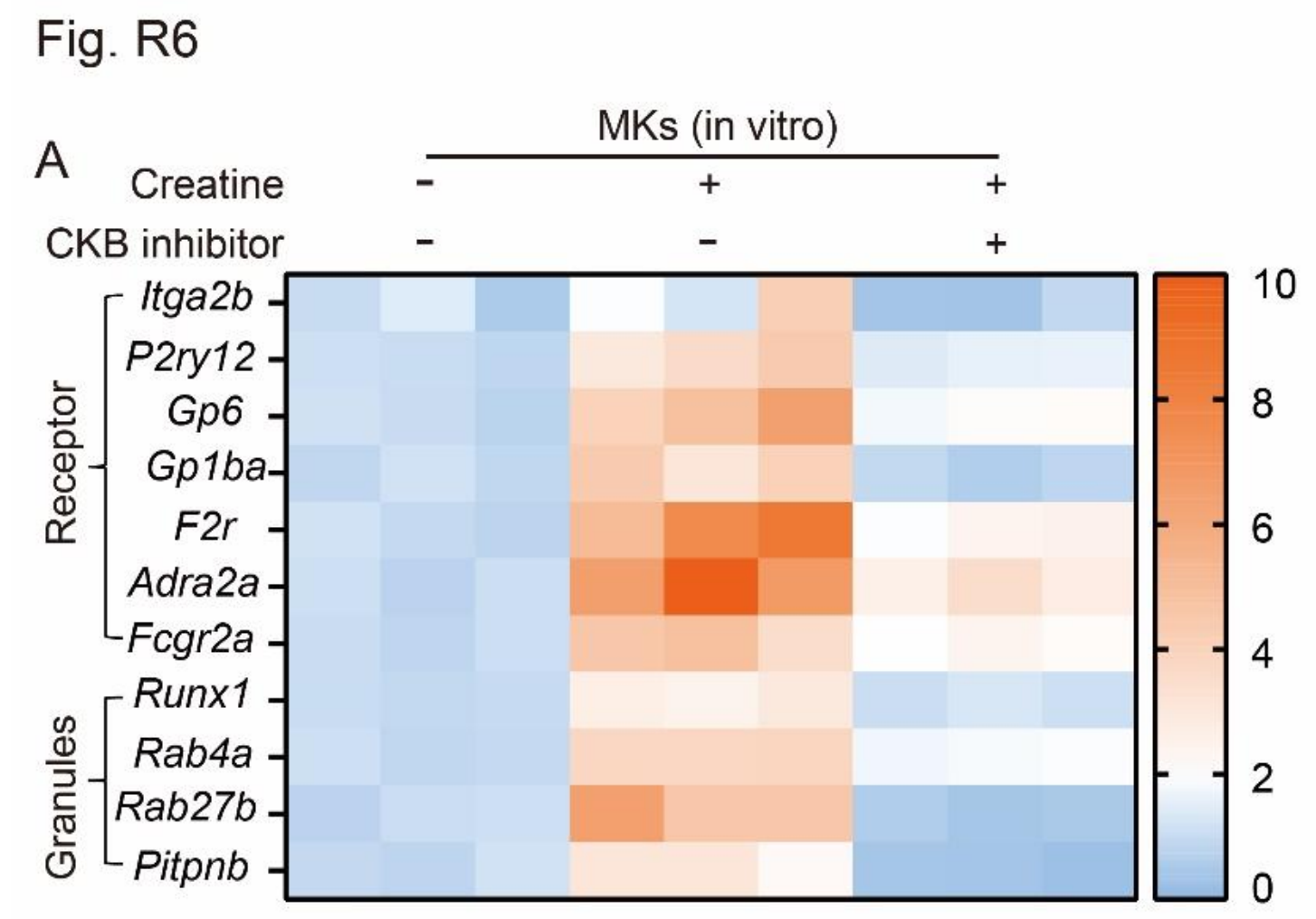

Fig. R6 (A) Heatmap of platelet receptor- and granule-associated genes in vehicle- or CKB inhibitor-treated MKs treated with or without creatine in vitro (*n* = 3 mice per group).

Comment: - I have technical concerns about megakaryocyte analyses. Characterization of bone marrow isolated megakaryocytes should be provided. DNA content analysis included in Sup. Fig. 3A is not convincing. It seems that cells display a very low ploidy level. Is this the case?

Response: We thank the reviewer for raising this important issue. We performed CD41 expression detection using an antibody recognizing both mouse and human CD41. Using MEG-01 as a positive control and isolated mouse monocytes as a negative control, we detected CD41 protein expression in our isolated megakaryocytes. As expected, isolated megakaryocytes express high levels of CD41, similar to the expression levels in MEG-01, whereas isolated monocytes express trace levels of CD41 (Fig. R7A).

In Sup. Fig. 3A, we show that the majority of isolated megakaryocytes are 16 N. 16 N is the most common ploidy level in mouse bone marrow megakaryocytes (PMID: 37055407). Although we agree that the purity of megakaryocytes isolated by density gradient centrifugation may not be as high as that obtained by flow cytometry sorting, these results suggest the purity of megakaryocytes was acceptable for subsequent analysis.

Fig. R7

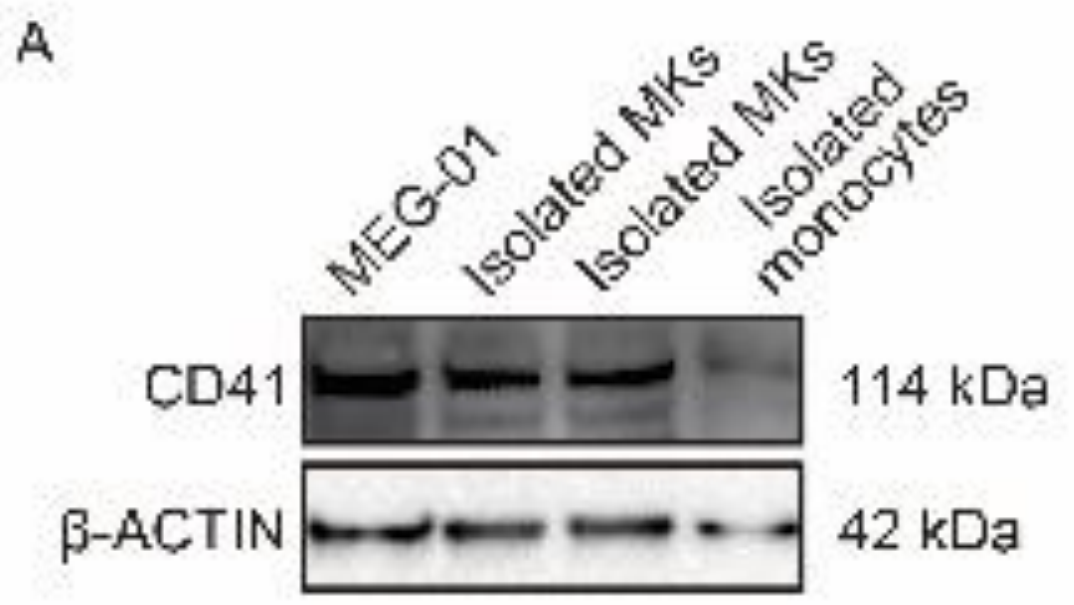

Fig. R7 (A) Western blot of CD41 expression in MEG-01 cells, isolated mouse bone marrow megakaryocytes, and isolated circulating monocytes. β-actin indicates loading amount.

Comment: - Similarly, the strategy of using CD41 cells isolated from peripheral blood of healthy subjects in human studies may not reflect megakaryocyte biology of the bone marrow. Additionally, cell identity used in this assay should be assessed. It is known that circulating platelets may attach to leukocytes, contaminating microbead separation strategies.

Response: Completely agree. In contrast to CD41+ circulating megakaryocytes, doublets of CD41+ platelets attached to nucleated WBCs should exhibit very low expression of *CD41* RNA expression. Therefore, to address the reviewer's concern, we isolated monocytes from the peripheral blood of newly recruited volunteers and performed examination of *CD41* RNA expression in these cells, as well as in previously isolated CD41+ cells. Furthermore, we applied the MEG-01 cell line, a human megakaryocytic leukemia cell line that is known to be CD41 positive, as a positive control. QPCR analysis revealed that, peripheral CD41+ cells expressed high RNA levels of *CD41* RNA (Fig. R8A), whereas *CD41* RNA expression was barely detectable in isolated human monocytes (Fig. R8A). The MEG-01 cell line served as a positive control for *CD41* RNA expression. These results suggest that our CD41+ nucleated cells are more likely to be circulating megakaryocytes than doublets of CD41+ platelets attached to nucleated WBCs.

Fig. R8

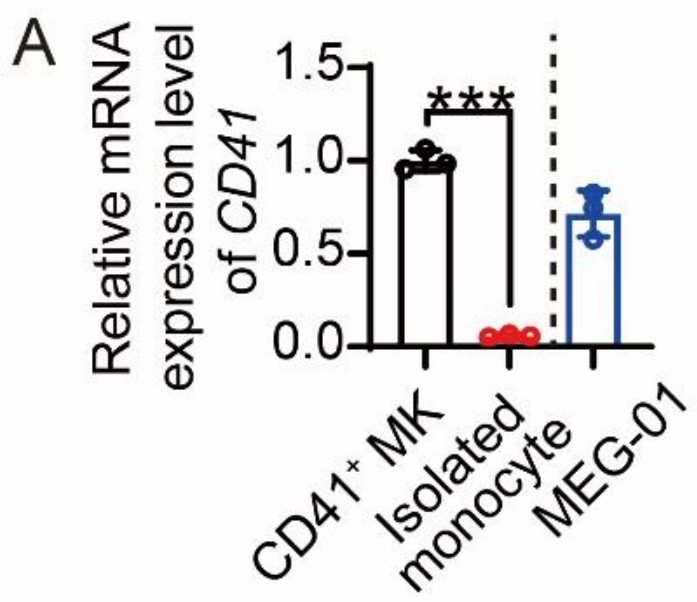

Fig. R8 (A) Quantitative PCR analysis of *CD41* RNA expression in isolated CD41+ cells from human peripheral blood, isolated monocytes from human peripheral blood, and human MEG-01 cell line (n = 3 samples per group).

These results have been incorporated into the Supplemental Figure 8C in the revised manuscript.

Comment: - What is the difference between "isolated MKs" and "isolated MKs in vitro"? Please specify.

Response: We apologize for this ambiguous label in Figures. "Isolated MKs" means freshly isolated MKs from various mouse groups. "Isolated MKs (in vitro)" means freshly isolated healthy MKs were treated with various drugs in vitro. We have revised these labels throughout all figures and supplemental figures.

Comment: - Sup. Fig. 5A shows that creatine supplementation does not affect mRNA levels of GATM and GAMT. However, the contribution of de novo creatine synthesis in these settings is not convincingly excluded.

Response: Completely agree. To exclude the contribution of de novo creatine synthesis in exogenous creatine-induced platelet activation, we used L-methionine [R,S]-sulfoximine, a GATM specific inhibitor that binds and disrupts the enzyme's catalytic activity. As expected, GATM inhibitor did not alter the exogenous creatine-induced protein expression in MKs (Fig. R9A), suggesting the de novo creatine synthesis is not critical in creatine-instigated hyperactive platelets.

Fig. R9

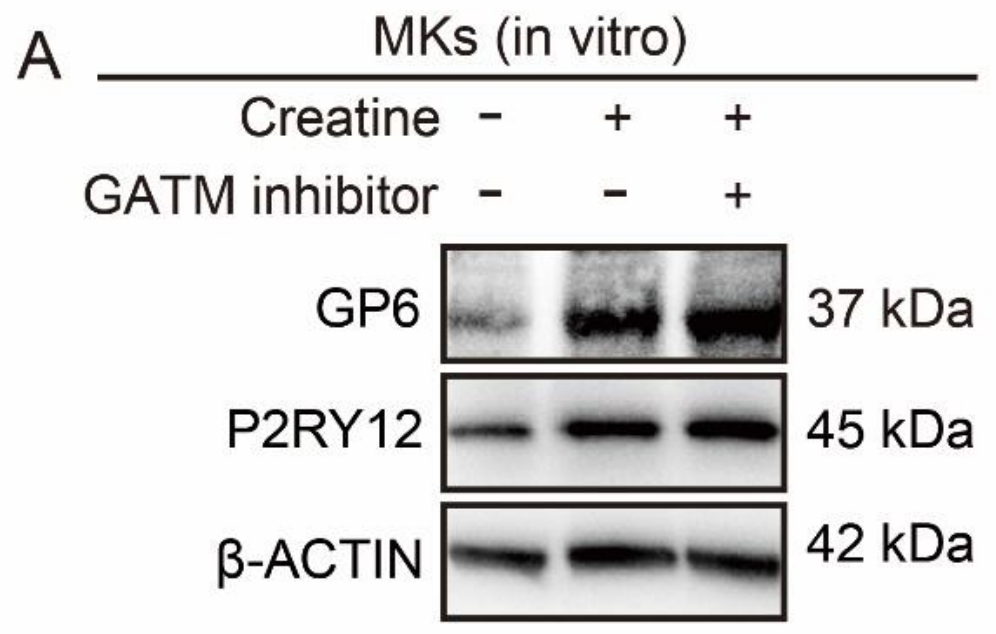

Fig. R9 (A) Western blot of GP6 and P2RY12 expression in MKs treated with creatine or GATM inhibitor. β-actin indicates loading amount.

Comment: - Platelets transfer experiments support the hypothesis of a mechanism restricted to the MK/plt lineage. I would suggest including any evidence that in vivo creatine supplementation in mice has no impact on other hematopoietic lineages.

Response: Completely agree. Following the reviewer's suggestion, we performed whole blood tests on vehicle- or creatine-treated mice. As expected, there was no change in WBC, lymphocyte, monocyte, and RBC counts upon creatine administration (Fig. R10A-D). These results are consistent with previous human studies that showed creatine supplements do not alter WBC or RBC counts (PMID: 12701816). These results suggest that short-term exogenous creatine treatment has no impact on the number of blood cells, at least in mice.

Fig. R10

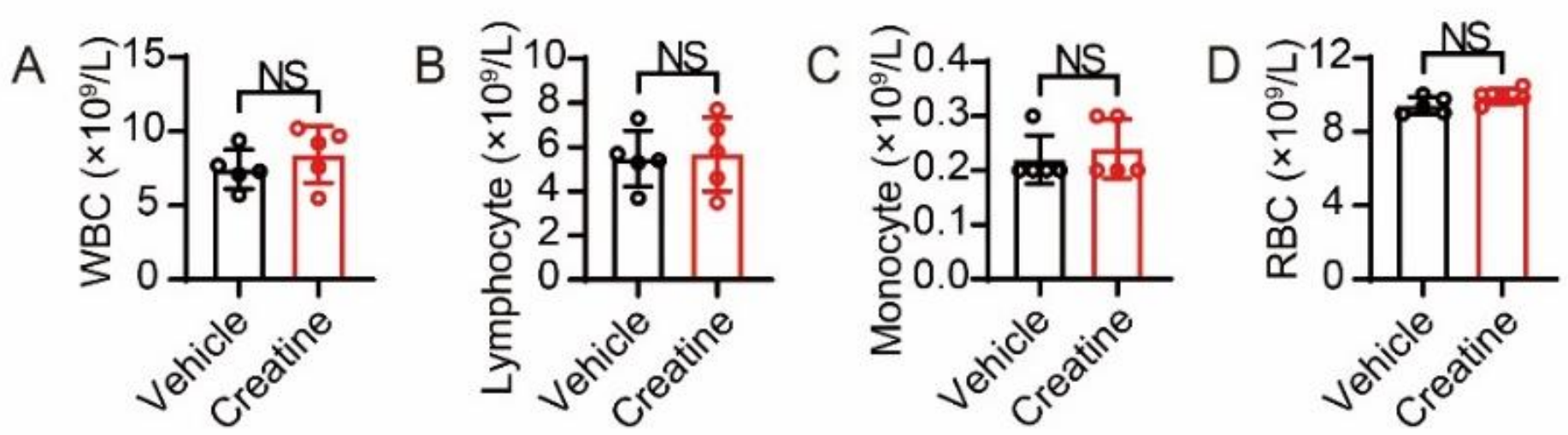

Fig. R10 (A-D) WBC, lymphocyte, monocyte, and RBC analysis in whole blood from

vehicle- or creatine-treated mice (*n* = 5 mice per group).

These results have been incorporated into the Supplemental Figure 3A in the revised manuscript.

Comment: - Figure 7. Western blot on before and after creatine supplementation platelets is missing.

Response: We thank the reviewer for this comment. We have provided western blots of GP6 and P2RY12 levels before and after creatine supplementation in peripheral platelets from 4 randomly selected volunteers (Fig. R11A). The majority of volunteers showed increased GP6 and P2RY12 levels in their platelets following creatine supplementation.

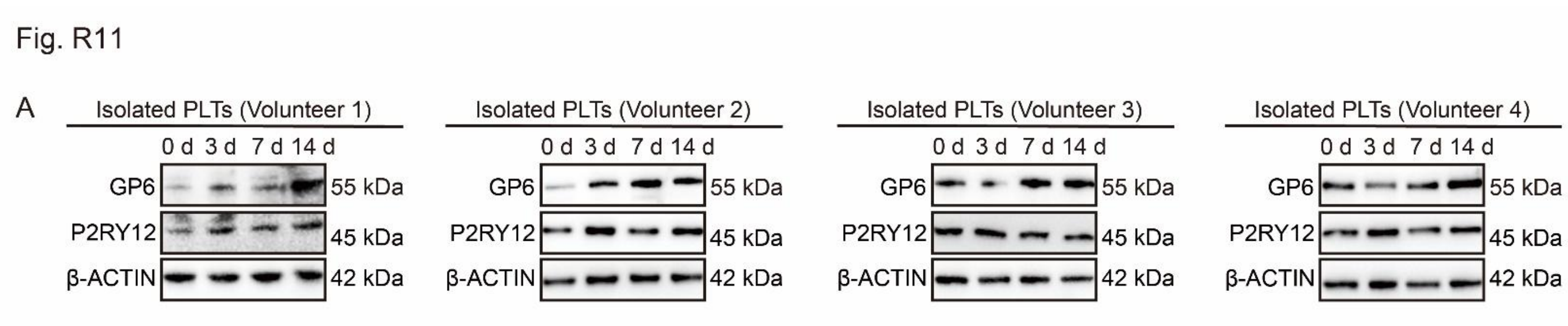

Fig. R11 (A) Western blot of GP6 and P2RY12 in isolated platelets from each volunteer at various timepoints. β-ACTIN marks the loading amount in each lane.

These results are in the Supplemental Figure 8C in the revised manuscript.

Reviewer: 5

Comment: Oral creatine at clinically relevant levels slowed clearance of intravenously injected tumor cells, increased lung metastases, and shortened survival across multiple mouse tumor models and both sexes; the effect was independent of tumor-cell SLC6A8, transferable by platelets, and attenuated by anti-platelet agents: aspirin and anagrelide. Mechanistically, creatine acts on megakaryocytes via SLC6A8 to produce hyperactive, tumor-adherent platelets—upregulating receptors (P2Y12, GPVI) and granule programs—through creatine-driven CKB upregulation that promotes non-canonical STAT5B phosphorylation, nuclear translocation, and transcription of platelet activation genes. MK-specific deletion of Slc6a8 or Stat5b, and pharmacologic STAT5 inhibition, abolishes effects. In humans, short-term creatine supplementation increases platelet activity and MK/platelet expression of P2RY12/GP6, and creatine-primed human platelets enhanced metastasis when xenotransferred into immunosuppressed mice.
This work is important, interesting, and well executed. There are some important questions that could significantly enhance the work.

Response: We thank the reviewer for correctly understanding our work and considering our work being important, interesting, and well executed. We deeply appreciate it.

Comment: 1. What happens in other metastatic models in addition to tail vein: intracardiac, or spontaneous?

Response: We thank the reviewer for this important comment. Following the reviewer's suggestion, we have performed a spontaneous metastasis model of 4T1 breast cancer in 20 BALB/c mice. We established a primary tumor and then treated the tumor-bearing mice with creatine once the primary tumor reached 0.5 cm^3. The primary tumor was removed once it reached 1.5 cm^3. Three weeks after tumor removal, mice were sacrificed for metastasis detection (Fig. R12A). As in our previous tail vein tumor cell injection model, we found that pulmonary metastasis was increased in the creatine-administrated group in the spontaneous metastasis model (Fig. R12B). Similarly, analysis of lung weight and metastatic burden confirmed that pulmonary metastasis was significantly increased in the creatine-administrated group (Fig. R12B). These results support that creatine supplementation promotes metastasis in spontaneous metastatic model.

REDACTED

Fig. R12 (A) Schematic diagram of the spontaneous metastasis model of 4T1 breast cancer in BALB/c mice. (B) Representative lung pictures and H&E histological analysis of lung metastasis. Dashed lines mark the borders between tumor (T) and lung (L) tissues. Arrowhead, visible metastatic nodules. Scale bar in upper panels, 1 cm. Scale bar in middle panels, 2 mm. Scale bar in lower panels, 50 μm. Quantifications of lung weight (*n* = 10 mice per group). Quantifications of metastatic burden and the number of metastatic foci (*n* = 10 mice per group).

These results have been incorporated into the Supplemental Figure 1J and K in the revised manuscript.

Comment: 2. Anagrelide reduces MK maturation and proplatelet formation, and its role requires clarification in this context (not platelet activation). Have the authors tried platelet depletion strategies, or conversely, antagonising acethycholine receptor to enhance platelet activation (opposite effect control) to specifically show this is activation mediated and not just thrombocytopaenia - ie via MK maturation effect?

Response: Completely agree. As the reviewer pointed out, other than inhibiting platelet activation, anagrelide may also reduce platelet formation. Following the reviewer’s suggestions, we performed additional pulmonary metastasis experiments. The negative control group involved injecting tumor cells alone without platelet transfer. Compared to transplanting tumor cell alone, co-transplanting healthy platelet did not increase the metastatic burden, although there was a trend towards an increase (Fig. R13A). These

results suggest that quiescent platelets insignificantly promote tumor metastasis, at least in our settings.

We further performed an opposite effect control. Fresh healthy platelets were isolated and treated with pancuronium bromide, a competitive AChR antagonist, at the dose of 10 nM for 15 min. Ach, similar to prostacyclin, is an endogenous platelet inhibitor. It is released by platelets themselves, and Ach-AchR signaling blockade in platelets in vitro has been proven to activate platelets (PMID: 30765424). These AChR-treated platelets were co-injected with tumor cells in our experimental metastasis model.

As a result, similar to creatine-induced hyperactive platelets, AChR antagonist-treated healthy platelets significantly increased tumor pulmonary metastasis burden (Fig. R13A), suggesting platelet activation is sufficient to promote pulmonary metastasis. We thank the reviewer for these important comments.

Fig. R13

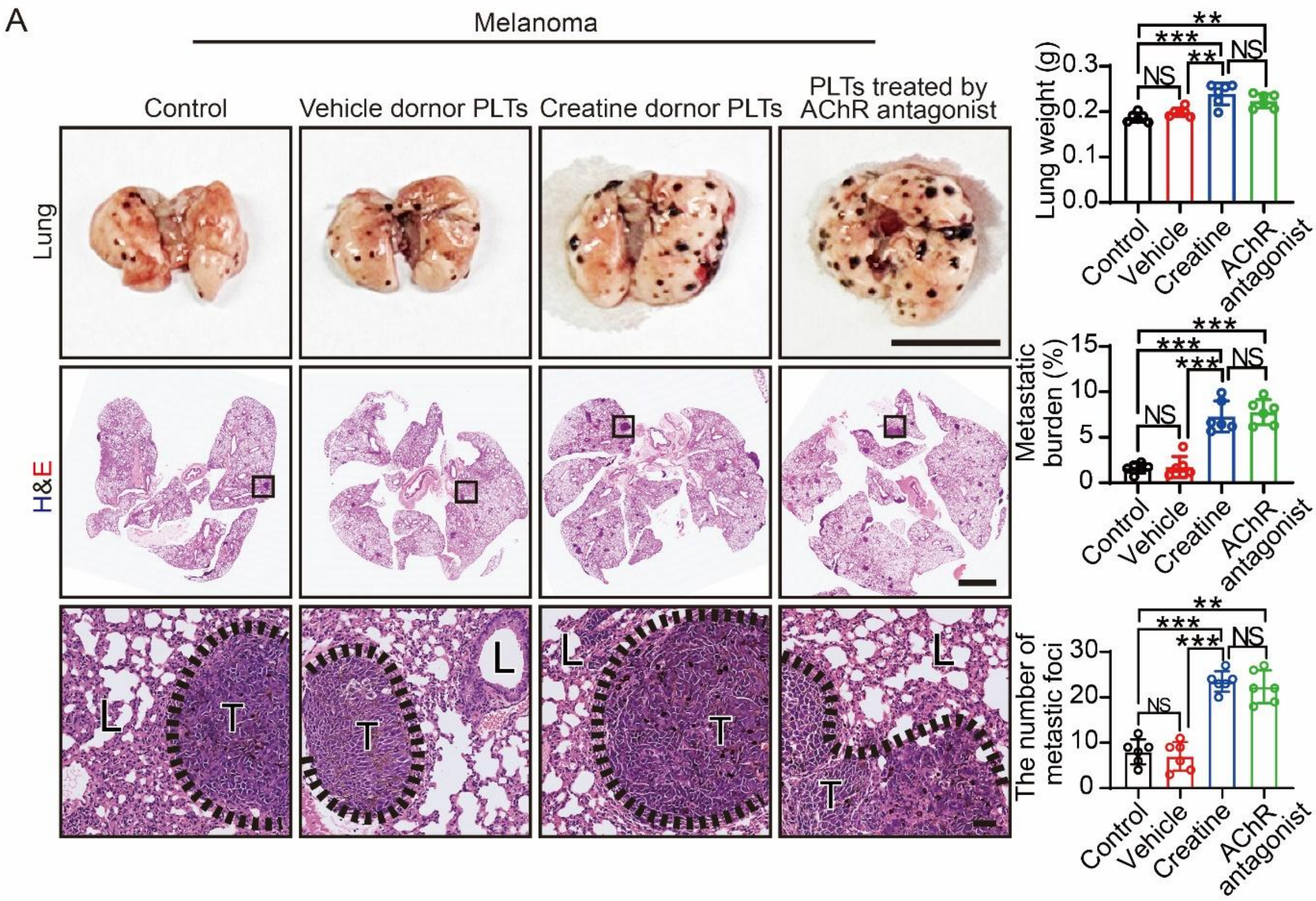

Fig. R13 (A) Platelets were isolated from vehicle- or creatine-treated donor mice, or isolated from healthy mice and treated with AChR antagonist in vitro. These platelets were co-injected with melanoma cells into healthy recipient mice. H&E histological analysis of lung metastasis. Dashed lines mark the borders between tumor (T) and lung (L) tissues. Scale bar in upper panels, 2 mm. Scale bar in lower panels, 50 μm. Quantifications of metastatic burden and the number of metastatic foci (*n* = 6 mice per group)

We thank the reviewer for this comment. We have removed anagrelide data in

Supplemental Figure 2D to avoid misunderstandings.

Comment: 3. Similarly, COX inhibition is broad range effective. Will aspirin/anagrelide given to recipient mice, not just ex vivo–treated donor platelets impact metastasis, and show impact on target TXB2 suppression in vivo.

Response: Completely agree. We administrated aspirin to recipient mice for 1 wk and then injected tumor cells. As expected, pulmonary metastasis was significantly decreased upon aspirin administration. We further tested TXB2 in this model using an TXB2 ELISA kit. TXB2 in the serum was higher in the creatine-treated group, while aspirin significantly inhibited TXB2 level. These results are in accordance with clinical observation that aspirin intake is associated with less tumor metastasis (PMID: 22440947).

Fig. R14

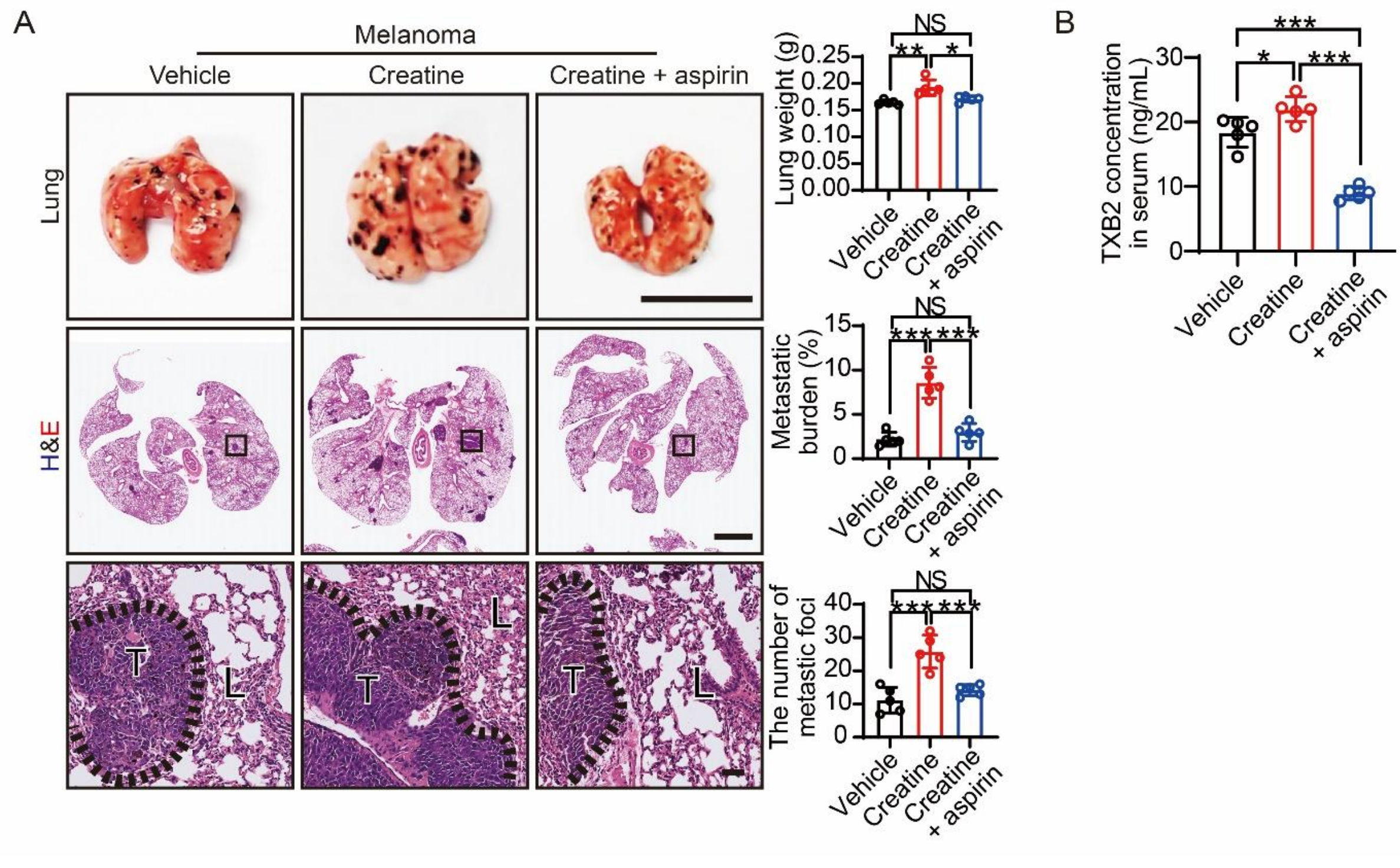

Fig. R14 (A) Melanoma cells were injected into vehicle-, creatine-, or creatine and aspirin-treated mice. Representative lung pictures with visible metastatic nodules. Quantifications of lung weight (*n* = 5 mice per group). H&E histological analysis of lung metastasis. Dashed lines mark the borders between tumor (T) and lung (L) tissues. Scale bar in upper panels, 1 cm. Scale bar in middle panels, 2 mm. Scale bar in lower panels, 50 μm. Quantifications of metastatic burden and the number of metastatic foci (*n* = 5 mice per group). (B) TBX2 concentration in serum samples from vehicle-, creatine-, or creatine and aspirin-treated mice. (*n* = 5 mice per group).

Comment: 4. could the authors show CTC aggregates with a marker for platelet activity in blood, for example p-selectin activation + GFP cells. similarly, they should rule out effects on lung fibrin by creatinine (d-dimer?), which could lead to more CTC trapping instead of platelet activation peripherally.

Response: Completely agree. According to the reviewer's suggestion, we performed FACS analysis in tumor cell-infused mice. After 24 h of tumor injection, circulating GFP+ tumor cells were gated from nucleated blood cells, and CD62P+ signals were also analyzed by FACS. As expected, the number of CD62P+ CTCs increased significantly by more than 3-fold in the creatine group compared with the vehicle group (Fig. R15A). We further tested the proportion of CD62P+ CTCs in total CTC population. Interestingly, platelet attached CD62P+ CTCs accounted for nearly 60% of the total CTCs in both groups, with a trend towards an increase in the creatine group (Fig. R15B). These results show that the majority of the remaining CTCs aggregate with platelets in the circulation in vivo, after 24 h.

Next, to rule out the possibility of CTC trapping, we detected d-dimer in the blood using a commercially available ELISA kit, as suggested by the reviewer. No significant changes were observed in plasma from vehicle- and creatine-treated mice (Fig. R15C). These results suggest that lung fibrin is not involved in the CTC trapping in creatine-treated mice.

REDACTED

Fig. R15 (A) Schematic diagram of the adoptive platelet transfer model. Platelets were isolated from vehicle- or creatine-treated donor mice and were co-injected with GFP+ melanoma cells into healthy recipient mice. Quantification of CD62P+ GFP+ cells in vehicle-donor platelet group and creatine-donor platelet group (*n* = 6 mice per group).

(B) Representative FACS image of gating strategy. Quantification of the proportion of CD62P+ GFP+ cells in total GFP+ cells in vehicle-donor platelet group and creatine-donor platelet group (*n* = 6 mice per group). (C) Quantification of D-dimer in blood samples in vehicle- and creatine-treated mice (*n* = 6 mice per group).

Comment: 5. Please present RNA data to rule out platelets are contaminant in the MK RNA and protein assays.

Response: We agree with the reviewer that CD41+ MK isolation may have contamination of CD41+ platelets attached to nucleated cells. To address this issue, we performed examination of *Cd41* RNA expression in these cells. CD41+ platelet attaching with nucleated leukocytes should have no expression of *Cd41* RNA expression. QPCR analysis revealed that, peripheral CD41+ cells expressed high RNA levels of *CD41* RNA (Fig. R8A), whereas *CD41* RNA expression was barely detectable in isolated human monocytes (Fig. R8A). The MEG-01 cell line served as a positive control for *CD41* RNA expression. These results suggest that our CD41+ nucleated cells are more likely to be circulating megakaryocytes than doublets of CD41+ platelets attached to nucleated WBCs.

Fig. R16

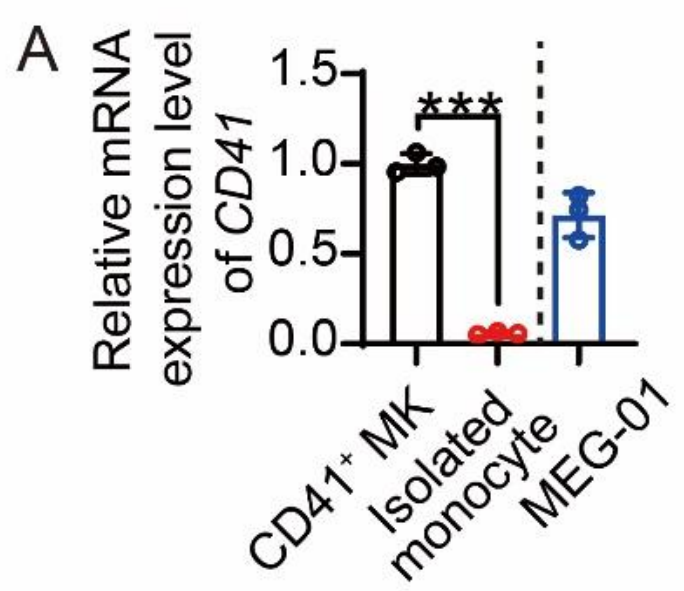

Fig. R16 (A) Quantitative PCR analysis of *CD41* RNA expression in isolated CD41+ cells from human peripheral blood, isolated monocytes from human peripheral blood, and human MEG-01 cell line (*n* = 3 samples per group).

These results have been incorporated into the Supplemental Figure 8C in the revised manuscript.

Comment: 6. does re expression of SLC6A8 (or creatine bypassing transporter) restore the phenotype?

Response: We thank the reviewer for this excellent suggestion. Following this suggestion, we adopted a transporter-bypassing strategy to perform the rescue experiment in *Slc6a8-/-* MKs. MKs were isolated from *Pf4-Cre; Slc6a8-/-* mice. These MKs were found to maintain low intracellular creatine levels despite high serum creatine levels in vivo (Fig. 3 in the previous version of manuscript).

We treated these MKs with 2 mM creatine in vitro. As expected, creatine administration did not increase the expression of platelet-associated genes. We next used liposomes to deliver creatine without using the creatine transporter. To establish the creatine@liposome, we made a 10 mg/ml solution of creatine in PBS as the aqueous phase. The creatine@liposome was made by ethanol injection method (DOI: 10.1016/j.jddst.2020.102174). Briefly, an ethanolic solution of lipids was injected into a large volume of the aqueous phase, leading to the rapid formation of vesicles. Upon injection, the unfavourable exposure of lipids to the aqueous medium results in the arrangement and precipitation of bilayer phospholipid fragments at the boundary between the ethanol and water phases. Interestingly, the administration of creatine@liposome elevated the expression of platelet-associated genes in creatine transporter-depleted MKs in vitro. These results support that creatine uptake in MKs, whether via the creatine transporter or other transmembrane mechanisms, promotes platelet activation gene expression. We thank the reviewer for this excellent suggestion to improve the quality of our work.

REDACTED

Fig. R17 (A) Schematic model of freshly isolated MK from *Pf4-Cre; Slc6a8-/-* mice receiving vehicle, creatine, or creatine@liposome. (B) Heatmap of platelet receptor- and granule-associated genes of isolated primary *Pf4-Cre; Slc6a8-/-* MKs treated with vehicle, creatine, or creatine@liposome (n = 3 samples per group)

These results have been incorporated into the Supplemental Figure 4D and E in the revised manuscript.

Comment: 7. are the human data stratified for NSAID intake.

Response: We apologize for not providing this important information in the study. All the human participants were free from NSAID intake. This information has been included in the revised manuscript in the Results section and the Supplemental Table 1.

Comment: 8. The authors need further work on the CKB/stat5b pathway, including in vitro assay KD CKB controls, of pS127 antibody. They need to show this is jak independent, that canonical y699 stat5 is not involved. could they do a crispr of other ko stat5b with wt v s127a in MKs to test s127 is necessary for genetic induction and platelet activity.

Response: We thank the reviewer for this comment. To address this concern, we examined JAK phosphorylation using a phospho-JAK Family antibody in vehicle- or creatine-treated MKs. This antibody recognizes JAK1 when phosphorylated at Tyr1034 and/or Tyr1035, JAK2 when phosphorylated at Tyr1007 and/or Tyr1008, JAK3 when phosphorylated at Tyr980 and/or Tyr981, and TYK2 when phosphorylated at Tyr1054 and/or Tyr1055. As expected, compared with positive controls, JAK was not phosphorylated in neither vehicle-treated MKs nor creatine-treated MKs (Fig. R18A), suggesting that creatine-induced STAT5B activation is JAK family-independent.

We agree with the reviewer that if we can deplete the endogenous STAT5B, and then transduce wt or S127A mutant STAT5B, we would demonstrate S127 is necessary for genetic induction of platelet activity. Therefore, we isolated MKs from PF4-Cre STAT5Bflox/flox mice then transfected wt or S127A mutant STAT5B. A similar amount of STAT5B-WT and STAT5B-127A was observed, whereas no STAT5B was observed in non-transfected PF4-Cre STAT5Bflox/flox MKs (Fig. R18B). Of note, canonical Y699 was not detectable in any of the three groups (Fig. R18C)

Next, both transfected groups were administered creatine in vitro. We already know that, compared to MK isolated from control mice, MKs from PF4-Cre STAT5Bflox/flox mice did not respond to creatine in terms of platelet-activation gene expression (Fig. 6 in main manuscript). As expected, transduction of STAT5B rescued this phenotype (Fig. R18D). In contrast, transduction of S127A mutant STAT5B blocked this phenotype (Fig. R18D). These results support our hypothesis that S127 site of STAT5B is critical for genetic induction and platelet activity. Once again, we thank the reviewer for improving the quality of our work.

Fig. R18

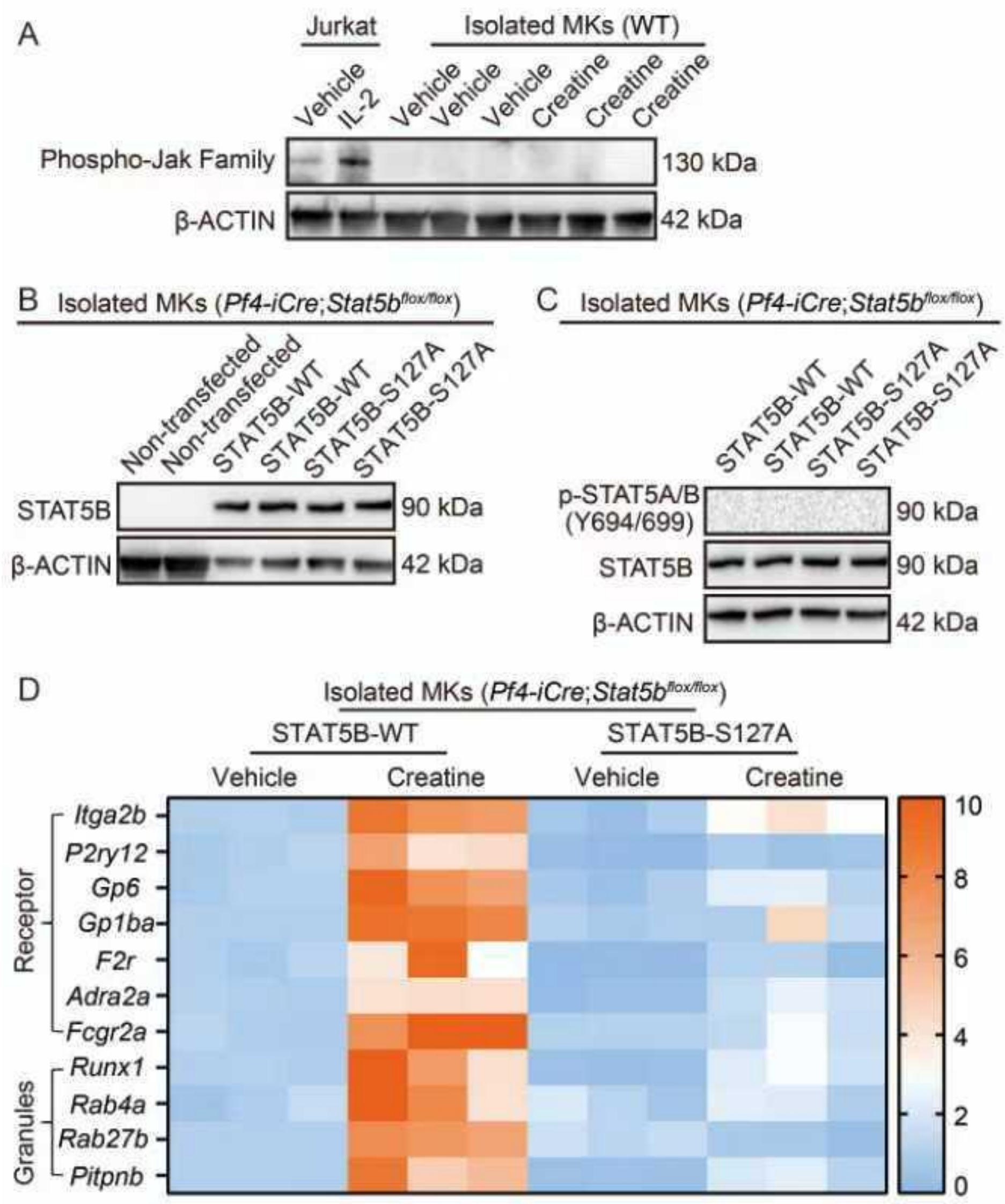

Fig. R18 (A) Western blot of phosphorylated JAK family in isolated wildtype MKs treated with vehicle or creatine. Jurkat T cells treated with or without IL-2 as positive and negative controls. β-actin served as loading controls. (B and C) Western blot of STAT5B and phosphorylated STAT5B (Y699 site) in isolated MKs from *Pf4* Cre *Stat5b*$^{\text{flox/flox}}$ mice. Non-transfected, STAT5B-WT-tranfected, or STAT5B-S127A-transfected MKs were shown. β-actin served as loading controls. (D) Heatmap of platelet receptor- and granule-associated genes in STAT5B-WT-tranfected, or STAT5B-S127A-transfected MKs (*Pf4* Cre *Stat5b*$^{\text{flox/flox}}$) treated with or without creatine in vitro (n = 3 mice per group).

Our point-by-point responses to the reviewers' comments are as follows:

Reviewer: 1
Comment: The authors have addressed the points I made to the previous version of the manuscript, and I have no further comments. In my view, the article is ready for publication.

Response: We thank the reviewer for improving the quality of our work.

Reviewer: 2
Comment: I co-reviewed this manuscript with one of the reviewers who provided the listed reports. This is part of the Nature Communications initiative to facilitate training in peer review and to provide appropriate recognition for Early Career Researchers who co-review manuscripts.

Response: We thank the co-reviewer for his/her effort in improving our work.

Reviewer: 3
Comment: The revised manuscript appears to satisfactorily address the majority of the requests by myself and the other reviewers. With respect to my suggestions, the authors cover the gaps and update the preciseness of their language to better convey their results, as well as address and revise the inconsistencies in their experimental design or presentation.

Response: We thank the reviewer for improving the quality of our work.

Reviewer: 6
Comment: Chen et al. show that exogenous creatinine supplementation promotes tumor metastasis via a previously unrecognized megakarocyte-mediated pathway leading to platelet hyperactivity. There studies are highly mechanistic and include a range of complementary strategies including in vitro and in vivo studies that leverage both pharmacological and genetic approaches. A small pilot study in humans extends the scope and relevance of their findings. The investigators have responded thoroughly

to the prior critique of their manuscript and have provided appropriate clarifications and verifications of their initial report.

Response: We thank the reviewer for acknowledging that we have responded thoroughly to the prior critique. We deeply appreciate it.
